# Functionally Graded Surfaces and Materials: From Fabrication to Biomedical Applications

**DOI:** 10.1021/acs.chemrev.5c00732

**Published:** 2026-01-29

**Authors:** Min Hao, Yidan Chen, Yuxuan Meng, Emily Yan, Jichuan Qiu, Younan Xia

**Affiliations:** † The Wallace H. Coulter Department of Biomedical Engineering, 1372 Georgia Institute of Technology and Emory University, Atlanta, Georgia 30332, United States; § School of Materials Science and Engineering, Georgia Institute of Technology, Atlanta, Georgia 30332, United States; ‡ School of Chemistry and Biochemistry, Georgia Institute of Technology, Atlanta, Georgia 30332, United States; ∥ State Key Laboratory of Crystal Materials, 12589 Shandong University, Jinan 250100, China

## Abstract

Functionally graded surfaces and materials, featuring spatial variations in terms of composition, structure, and other properties across distance, have emerged as powerful platforms for mimicking native tissue architectures and enabling a wide range of biomedical applications. This review aims to provide a comprehensive overview of their fabrication methods and biomedical applications. We begin by introducing the concept of gradients and their inherent biological relevance in nature. With a distinct focus on either surfaces or materials, we then discuss the fabrication methods and characterization techniques capable of controlling the graded profiles. Importantly, representative examples are provided to highlight how engineered gradients regulate specific cellular responses and functionalities in biomedical contexts. Despite significant progress, challenges remain in translating laboratory-scale fabrication to clinical use, such as ensuring good reproducibility and scalability. At the end, we discuss how computational modeling and artificial intelligence offer new opportunities to address these challenges. We hope this review provides a framework for advancing the development of next-generation functionally graded surfaces and materials toward diverse biomedical applications.

## Graphical abstract

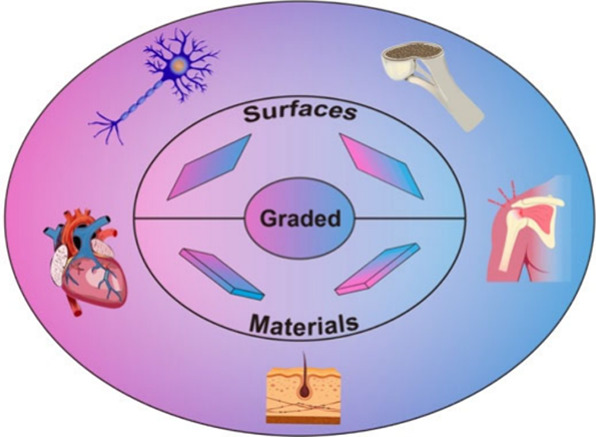

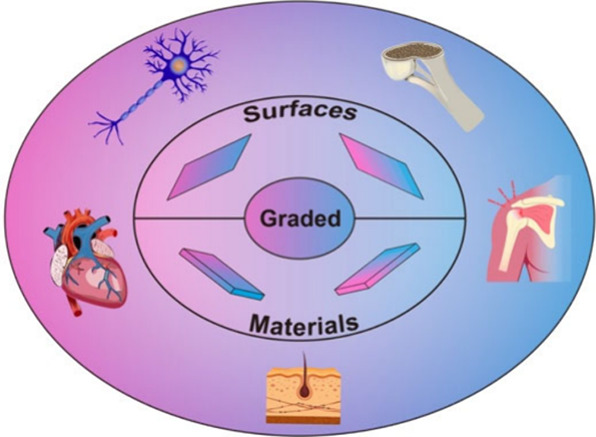

## 1 Introduction

Fabrication of surfaces and materials with tailored properties is essential to biomedical engineering, a field that constantly draws inspiration from nature. One of the most intriguing design principles used by nature is gradation, which involves gradients (either continuous or stepwise changes) in terms of material composition, structure, and other properties across a surface or throughout the bulk. Gradation plays a vital role in integrating different types of materials, mimicking the complex biochemical and/or architectural environments of native tissues, and creating spatially resolved properties with advanced functionalities that homogeneous surfaces or materials fail to provide. In this review, we define materials with gradients at or near the external surface as functionally graded surfaces, whereas those featuring engineered gradients throughout the bulk are referred to as functionally graded materials. Gradients can be presented linearly along a single direction (1D), radially out from a central point (2D), or multidirectionally, such that the property varies along every direction within three-dimensional (3D) space, as exemplified by the intricate gradient-index structure of the eye. Figure 1A shows a summary of the different types of gradients. It is important to note that the dimensionality of a gradient does not determine the classification as a surface or bulk phenomenon. A 1D gradient can exist either on a surface as a functionally graded surface or in the bulk as a functionally graded material (Figure 1B). Based on the exact profile, gradients can be categorized as either continuous or stepwise (Figure 1C). Continuous gradients exhibit a smooth and uninterrupted transition in terms of material composition or properties, with no clear boundaries between regions of different characteristics. In contrast, stepwise gradients consist of distinct, segmented regions with uniform properties within each segment.

**Figure 1:**
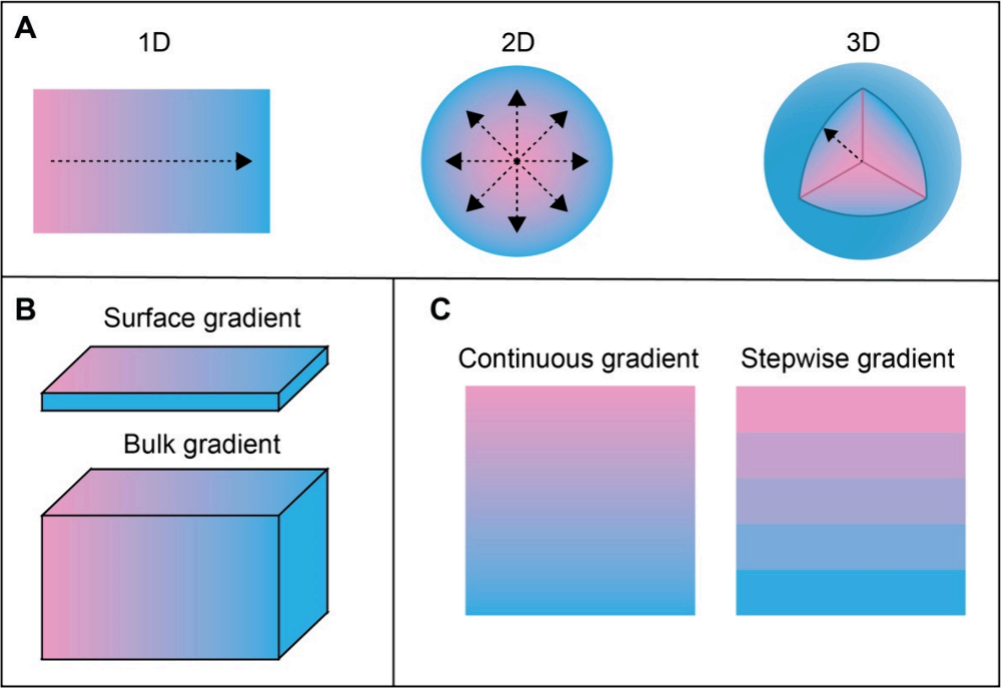
Illustration of the concepts related to functionally graded surfaces and materials. (A) 1D, 2D, and 3D gradients; (B) surface vs. bulk gradient; and (C) continuous vs. stepwise gradient.

A variety of physical and chemical gradients can be fabricated in a rational and controllable manner (Figure 2). The gradients can be categorized based on their physical nature, which dictates the properties or functions of the surface or material. Gradients in composition represent spatial variations in elemental distribution (Figure 2A) and are fundamental to many functionally graded surfaces or materials. The gradients in composition often lead to additional types of gradations. For example, transitioning from a pure polymer to a polymer composite with inorganic fillers creates a gradient in mechanical properties.^1^ Structural gradients encompass a broad spectrum of variations in material architecture, including particle density, porosity, fiber density, or grain orientation (Figure 2B). Both compositional and structural gradients can be designed to enhance the overall performance of a surface or material (Figure 2C). For example, a gradient in surface chemistry can create a gradation in hydrophilicity to help control protein adsorption and cellular adhesion.^2^ Varying the degree of polymer cross-linking spatially creates a gradient in network density, leading to controlled swelling characteristics and drug release kinetics.^3^ Gradients in mechanical properties, such as Young’s modulus and hardness, can be leveraged to reduce stress concentrations at interfaces and guide spatially controlled cell differentiation.^1^

**Figure 2:**
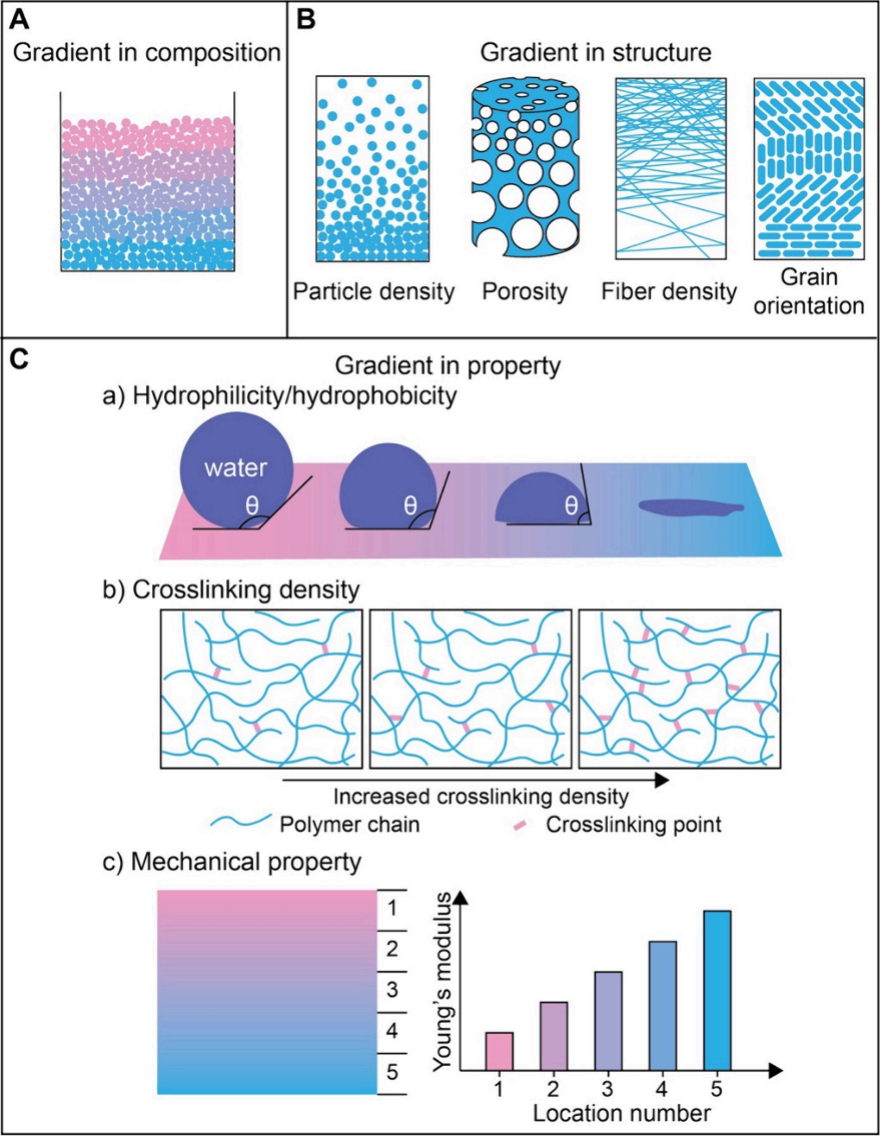
Illustrations of different types of gradients: (A) Composition, (B) structural features, including particle density, porosity, fiber density, and grain orientation, and (C) properties, for example, hydrophilicity/hydrophobicity, cross-linking density, and mechanical property.

Nature offers numerous examples of functionally graded structures that are often refined through the evolutionary process to achieve optimal performance. For instance, the remarkable resistance of bamboo culms to external loads is due to their 2D-graded structure (Figure 3A). The structure consists of stiff vascular bundles of fibers (with an elastic modulus of 22.8 ± 2.8 GPa) embedded in soft, porous parenchyma cells (with an elastic modulus of 3.7 ± 0.4 GPa).^4^ The density of the reinforcing fiber bundles in bamboo culms decreases from the circumference toward the center, resulting in a distinctive graded architecture. This design provides high strength and toughness, enabling the structure to absorb substantial energy and thus resist crack propagation when subjected to bending loads from the wind and/or its weight.^5^ Similarly, the formidable spines of a cactus serve as both protective features and specialized, modified leaves with an efficient mechanism for collecting water from fog in arid environments.^6^ Cactus spines feature a conical structure with graded microgrooves, whose width increases from ca. 4.3 μm at the tip to 6.8 μm at the base (Figure 3B). This geometry creates two synergistic gradients that facilitate the directional movement of collected water droplets along the spine. The first gradient is in Laplace pressure that arises from the conical shape of the spine. The second gradient is in surface energy created by the varying surface roughness on the grooves. The two gradients work synergistically, contributing to the efficient transport of water from the tip to the base of the spine.

**Figure 3:**
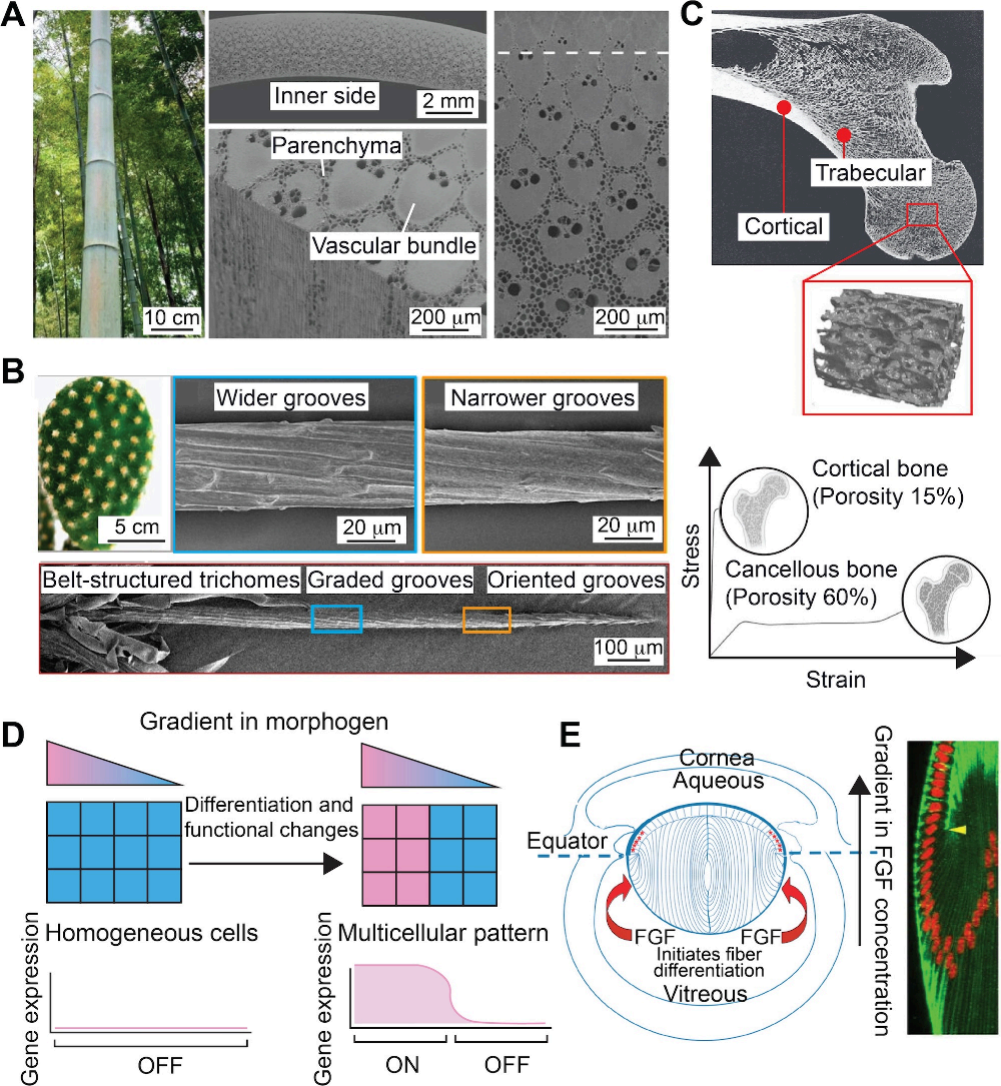
Gradients in biological systems. (A) Optical micrographs of bamboo and cross-sectional scanning electron microscopy (SEM) images of the bamboo culm, which have gradients in both vascular fiber bundles and porous parenchyma cells. (B) Optical micrographs of a cactus with clusters of spines and SEM images of a single spine showing the conical structure and graded grooves. (C) Cross-sectional image of a human femur with a graded porosity and the stress/strain curves of the cortical and trabecular bone. (D) Schematic illustration of a gradient in morphogen that directs cell differentiation. (E) Schematic illustration of a rodent eye showing the polarity of the lens determined by a gradient in FGF. (A) Reproduced with permission from ref 21. Copyright 2023 Wiley-VCH. (B) Reproduced with permission from ref 6. Copyright 2012 Springer Nature. (C) Reproduced with permission from ref 22 and 23. Copyright 2023 The Author(s) (CC BY 3.0). Copyright 2013 Springer-Verlag Berlin Heidelberg. (D) Reproduced with permission from ref 24. Copyright 2023 The Author(s). (E) Reproduced with permission from ref 17 and 25. Copyright 2011 The Royal Society. Copyright 2024 The Company of Biologists.

The human body also showcases various examples of functionally graded materials. Bone, for instance, exhibits a well-defined structural gradient, transitioning from dense cortical bone on the exterior to porous, spongy cancellous bone in the interior (Figure 3C). While the cortical exterior provides high mechanical strength,^7^ the porous interior distributes stress throughout the bone epiphysis, enhancing its ability to withstand both bending and compressive loads.^8,9^ Chemical gradients also play a crucial role in the biological organization beyond the static structures. In developmental biology, a gradient in morphogen, characterized by varying concentrations of signaling molecules, is vital during embryogenesis.^10,11^ The gradients control the fate of cells and dictate their development into tissues and organs (Figure 3D). The key morphogen families include fibroblast growth factor (FGF), Wnt, Hedgehog, and the transforming growth factor (TGF) superfamily that encompasses bone morphogenetic protein (BMP).^12^ In the context of FGF, a gradient in concentration is often established in the target tissue through diffusion. The resulting gradient has a major impact on the expression of specific genes and the subsequent specification of cells in a dose-dependent manner. For instance, in the developing chick embryo, a gradient in FGF generates varying levels of Hox-c gene expression, which is instrumental in controlling the positional identity of motor neurons.^13–15^

The human eye also relies on a complex, gradient-based design to govern both development and function. In the cornea, fibroblast populations play a crucial role in maintaining optical clarity and resilience.^16^ Additionally, a gradient in FGF regulates the polarity and differentiation of lens epithelial cells *in situ*.^17^ The cells have a dose-dependent response: low FGF concentrations induce proliferation, whereas higher concentrations promote migration and differentiation into lens fiber cells (Figure 3E).^18^ The developmental process is essential to the formation of the key functional feature of the lens: a gradient in refractive index. Specifically, the concentration of Crystallin proteins is the highest at the core of the lens. It gradually decreases toward the periphery,^19^ resulting in a corresponding gradient in refractive index from the center to the outer surface.^20^ Such a gradient enables the lens to tightly focus light onto the retina with minimal spherical aberration, a feature difficult to replicate in artificial lenses.

Among the most illustrative and studied natural functionally graded materials is probably the tendon-to-bone insertion, known as the enthesis. Such a specialized transitional tissue connects and transmits load between two mechanically dissimilar tissues: the compliant tendon (with a tensile modulus of ca. 200 MPa),^26^ and the stiff bone (with a modulus as high as 20 GPa).^27^ The enthesis mitigates stress concentration that tends to occur at such an abrupt interface by employing a seamless gradient in the biochemical composition, structure, mechanical properties, and cellular makeup. Typically, a fibrocartilaginous enthesis can be divided into four distinct yet continuous zones: unmineralized tendon, unmineralized fibrocartilage, mineralized fibrocartilage, and mineralized bone (Figure 4).^28,29^ There is a progressive increase in mineral content that is accompanied by a gradual reduction in tissue organization and a shift in collagen composition from type I to types II and X. Structurally, the enthesis features an interdigitated arrangement of mineralized and unmineralized tissues, a design critical for balancing strength and toughness to alleviate stress at the interface.^30^ Notably, cell phenotypes also gradually vary across the enthesis, from tenocytes in the tendon, unmineralized and mineralized (hypertrophic) chondrocytes residing in the unmineralized and mineralized fibrocartilage, and osteocytes occupying the bone. Gradients in biological and mechanical factors play a crucial role in establishing and maintaining the structural complexity of the enthesis.^31,32^ Despite advancements in surgical techniques, injuries to the enthesis remain challenging to repair, with clinical efforts often hampered by high postoperative failure rates. The native functionally graded structure is hardly regenerated; instead, mechanically inferior scar tissue forms at the tendon-to-bone attachment. As a result, critical features, such as a spatially graded composition and structure of the extracellular matrix (ECM) and a unique population of cells with a phenotypic gradient, are not recreated, leading to poor healing outcomes.^33^ The enthesis not only serves as a paradigm for naturally graded materials but also highlights the critical importance of biomimetics in biomedical engineering.

**Figure 4:**
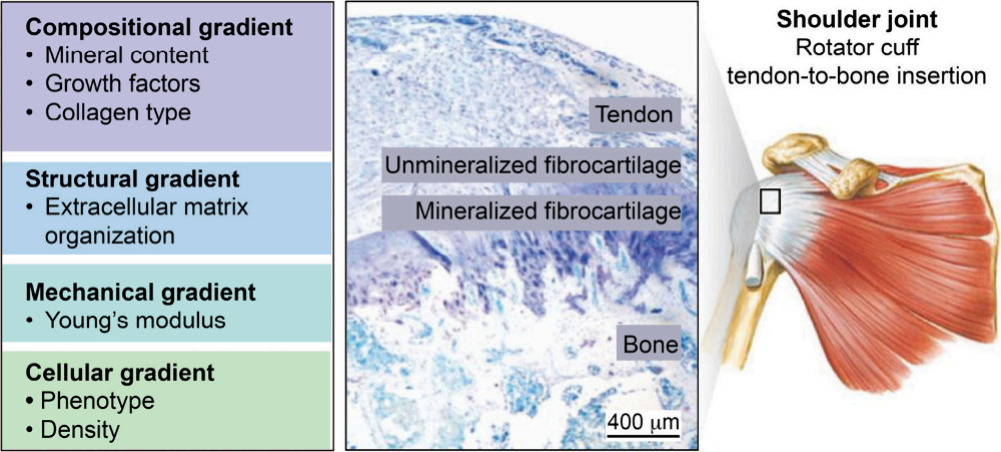
Natural functionally graded architecture of the tendon-to-bone enthesis. The interface displays inherent biochemical, structural, mechanical, and cellular gradients that ensure efficient load transfer from compliant tendon to stiff bone. Reproduced with permission from ref 34. Copyright 2021 Wiley-VCH.

Biological systems with compositional and structural gradients have inspired the fabrication of functionally graded surfaces and materials, where properties like stiffness, density, porosity, chemical composition, or even biological activity vary spatially in a controlled manner. In this review, we systematically survey the diverse fabrication methodologies developed for creating functionally graded surfaces and materials, encompassing both established techniques and emerging approaches. We then highlight the rapidly expanding applications of the functionally graded surfaces and materials in biomedicine, including but not limited to advanced scaffolds for tissue engineering, platforms for neural regeneration and wound healing, and integrated diagnostic systems. A comprehensive understanding of the design principles that govern the formation of gradients, together with mastery of an evolving fabrication toolkit, is critical for unlocking the full potential of functionally graded surfaces and materials to address the persisting and emerging challenges in biomedical field.

## 2 Fabrication of Functionally Graded Surfaces

Functionally graded surfaces feature gradations in chemical, physical, and/or biological properties across the surface of a substrate. Here we focus on four fabrication strategies, including progressive immersion, mask-assisted, field-induced, and microfluidic-enabled deposition. Built on distinct principles, they have different capabilities and limitations in creating functionally graded surfaces.

### 2.1 Progressive Immersion

Progressive immersion involves the gradual insertion of a flat or curved substrate into a reaction mixture that contains the functional components, such as reagents, cross-linking agents, nanoparticles, and living cells, among others.^35–38^ This approach offers an easy and programmable control throughout the reaction and/or deposition, leading to the formation of gradients in terms of composition, reaction extent, and/or material properties. Depending on the substrate geometry and immersion configuration, the resulting gradient can take a 1D (Figure 5A–D) or 2D (Figure 5E) pattern. In the following sections, we discuss the general strategies, together with examples to demonstrate their promise in creating surface gradients in terms of biomolecular, mineral, or cellular components.

**Figure 5:**
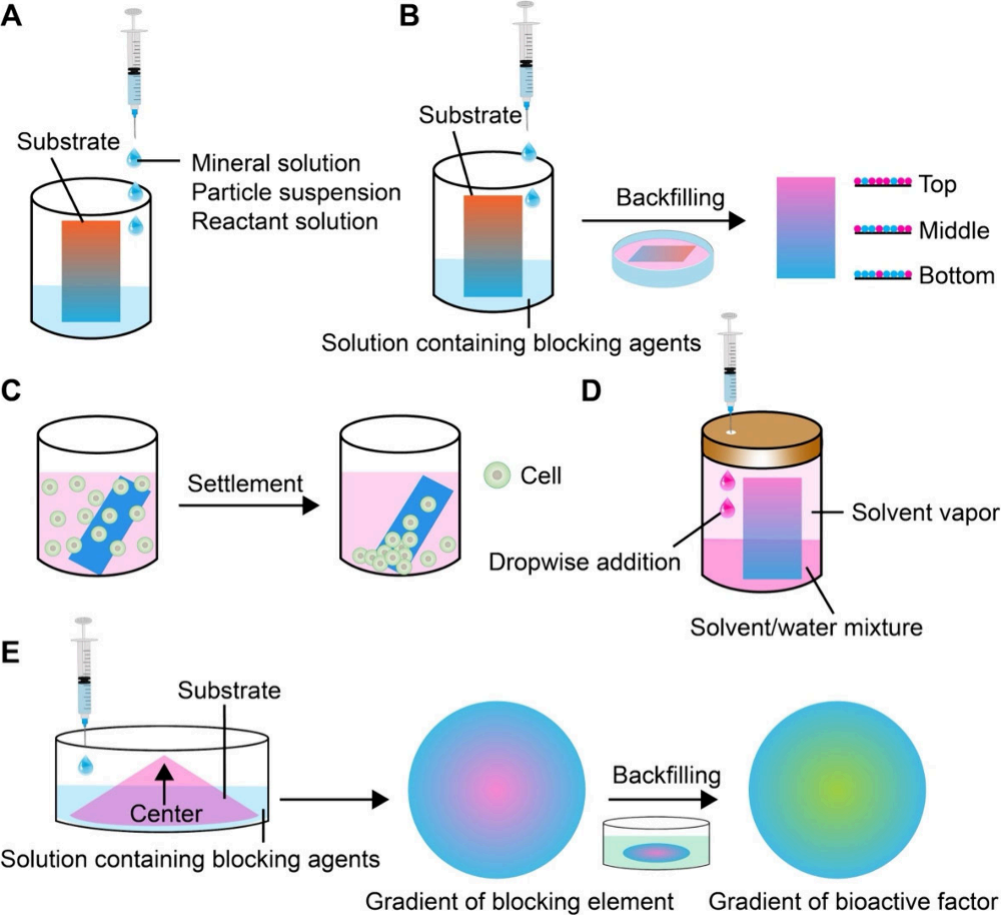
Schematics showing the preparation of surface gradients via progressive immersion. (A, B) Fabrication of a surface gradient by gradually immersing a substrate into a reaction solution or through backfilling. (C) Generation of surface gradient in cell density. (D) Gradient in fiber alignment and/or porosity achieved by controlling the exposure time of a nanofiber mat to the vapor of a solvent. (E) Creation of 2D gradients in bioactive proteins with the use of a blocking agent. Reproduced with permission from ref 45. Copyright 2024 The Authors (CC BY 4.0).

#### 2.1.1 Flat Substrates

As shown by the schematic in Figure 5A, a 1D gradient can be readily fabricated by controlling the introduction of a solution or suspension into a container that holds a vertically oriented or tilted flat substrate.^39^ To enable time-dependent immersion, the solution or suspension is typically added using a flow-regulated device such as a buret, syringe pump, or high-performance liquid chromatography pump.^40^ As the liquid level gradually rises, each horizontal segment of the substrate experiences a different immersion time, with the bottom having the longest contact. As a result, a 1D gradient of an insoluble substance or nanoparticles is formed along the direction of immersion. In general, the surface gradient is determined by the duration of interaction between the surface and the insoluble substance or nanoparticles in the liquid medium, including physical adsorption (e.g., hydrogen bonding), electrostatic attraction, and chemical reaction(s).^39,41^

One notable example involves electrostatic attraction via the gradual introduction of anionic Au nanoparticles, preconjugated with a protein of interest such as bovine serum albumin (BSA), ephrin-A5, or ephrin-B1, into a container holding a substrate precoated with cationic molecules.^40^ Due to the gradual introduction, the bottom portion of the substrate remained in contact with the suspension for the longest duration, leading to the highest density of Au nanoparticles in that region. Therefore, the Au nanoparticles were distributed in a graded manner along the vertical direction of the substrate. In the case of a chemical reaction, Kilbey and co-workers employed a substrate coated with a poly­(2-vinyl-4,4-dimethylazlactone) brush and gradually immersed it into a solution of a primary amine (e.g., hexylamine) using a syringe pump.^42^ The azlactone-amine reaction occurred in a time-dependent manner, resulting in a gradient in hydrophobicity across the surface. In both examples, parameters such as the concentration of the insoluble substance or nanoparticles, the injection rate, and the tilting angle of the substrate can all be adjusted to tune the gradient.

An alternative strategy for generating 1D surface gradients involves backfilling of the remaining regions on the substrate (Figure 5B). The first step is to form a primary gradient using a blocking agent such as BSA.^43^ Subsequently, a second bioactive molecule is introduced to occupy the blank regions, creating a complementary or reverse surface gradient. The two-step approach enables the integration of distinct bioactive factors within a single system, facilitating the coordination of cell adhesion, migration, and differentiation. In one demonstration, a dual gradient was created on the surface of an electrospun polycaprolactone (PCL) nanofiber mat using the backfilling strategy.^43^ Specifically, a gradient in BSA density was generated first by vertically immersing the mat in a beaker while slowly introducing a BSA solution at a constant rate. Due to time-dependent adsorption, the density of BSA decreased from the bottom to the top of the mat. The mat was then completely immersed in a solution containing the nerve growth factor (NGF), which adsorbed primarily onto the blank regions spared by BSA, resulting in the formation of a reverse gradient in NGF density. This method helps conserve expensive proteins and enables the fabrication of substrates or scaffolds featuring spatially orchestrated biochemical signals.

Besides generating gradients in substances or nanoparticles, progressive immersion can also be adapted to fabricate gradients in cell density or cell phenotype (Figure 5C). In a typical process, a substrate such as a glass slide or nanofiber mat is immersed at an inclined angle in a homogeneous suspension of cells.^38^ Driven by gravitational sedimentation, cells settle and adhere to the surface of the substrate in a graded manner owing to the different volumes of suspension present above each segment of the tilted substrate. The slope and extent of the resulting gradient in cell density can be controlled by adjusting the tilting angle, immersion time, and/or cell concentration in the suspension. The method can also be used to produce reverse or bidirectional gradients by sequentially immersing the substrate in suspensions of two different types of cells from opposite ends. For example, preosteoblasts and fibroblasts could be deposited in reverse gradients to form a transition zone that mimics the native interfacial tissues.^38^ Overall, this technique is simple, versatile, and compatible with both smooth substrates and fibrous mats. The ability to preserve high cell viability and generate complex patterns of cells makes it well-suited for applications such as tendon-to-bone tissue regeneration, where gradual transitions in cell phenotype and density are crucial for proper integration and mechanical performance.

Beyond the solution-based techniques, vapor-induced welding provides a robust method for generating structural gradients on polymeric substrates, such as electrospun nanofiber mats (Figure 5D). This method exploits the difference in swelling and welding of polymeric nanofibers when they are exposed to the vapor of a solvent. Typically, a nonwoven mat comprised of electrospun poly­(lactic-*co*-glycolic acid) (PLGA) or PCL nanofibers is placed in a sealed vial containing an aqueous solution of ethanol.^44^ In the liquid phase, the hydrogen bonding between ethanol and water restricted the diffusion of ethanol into the nanofibers. In contrast, ethanol molecules in the vapor phase could quickly diffuse into the nanofibers. The difference in mobility and swelling capacity resulted in a continuous structural gradient, transitioning from a highly porous mat at the bottom to a dense, film-like morphology at the top. The gradient profile can be tuned by adjusting the concentration of ethanol, exposure time, and/or solvent volatility, allowing for a tight control over the sample morphology. This vapor-mediated method is simple, scalable, and compatible with various fibrous substrates, eliminating the need for complex instrumentation or patterning masks.

Recent studies also demonstrated the potential use of vapor-induced welding for engineering biomimetic scaffolds with spatially varying architectures. For example, PLGA or PCL fibrous mats with graded welding were used to guide directional cell alignment, modulate cell infiltration depth, and mimic interfacial tissues such as the tendon-to-bone insertion.^44^ The ability to modulate porosity and fiber architecture at micro- to macroscopic scales makes this technique especially promising for applications in interfacial tissue engineering and wound management.

#### 2.1.2 Nonflat Substrates

Building on the concept of progressive immersion used for generating 1D gradients on flat substrates, a similar method has also been developed to fabricate 2D gradients on nonflat substrates (Figure 5E). The approach leverages the geometry of the substrate to create position-dependent immersion time across the surface in a radial fashion, resulting in radial variations in surface composition or functionality. A typical setup involves a flexible substrate partially elevated and supported by a pillar, spacer, or wires at the center to produce a conical or dome-like shape. As the reaction mixture is gradually introduced, different regions of the substrate experience variable exposure times. The peripheral region encounters the solution first and remains immersed for the longest period, while the central area has the shortest contact duration (or vice versa, depending on the orientation of the cone-shaped substrate). The spatial variation in immersion time gives rise to a radially graded distribution of the functional component.

As with 1D gradients, the backfilling technique can also be seamlessly integrated to introduce a second bioactive component, generating a complementary graded pattern.^43,45^ For example, after establishing a 2D gradient of a blocking agent such as BSA, a second bioactive factor can be introduced to occupy the bare regions, producing a reverse gradient.^46^ This two-step method allows for the formation of dual gradients, greatly expanding the design flexibility for the creation of complex, biomimetic environments. Again, the gradient profile can be tuned by adjusting several parameters, including the height of central elevation, solution concentration, immersion duration, and/or temperature. Importantly, this method is compatible with a wide range of substrate materials and surface chemistries and does not require specialized equipment. The geometrically driven nature makes it especially suitable for applications that mimic symmetrical tissue architectures or require spatial control of biochemical and topographical cues, such as neural patterning, wound healing, or stem cell niche design.

#### 2.1.3 Examples

To illustrate the practical implementation of progressive immersion in creating surface gradients, we present two examples that involve the fabrication and characterization of 1D (Figure 6A,B) and 2D (Figure 6C,D) gradients. In the first example, a 1D mineral gradient was fabricated on an electrospun nanofiber mat to mimic the natural tendon-to-bone insertion (Figure 6A). A nonwoven mat composed of plasma-treated electrospun PLGA or PCL nanofibers was placed at an inclined angle inside a glass vial. Then, 10 times concentrated simulated body fluid was introduced at a constant injection rate.^35^ Different regions on the nonwoven mat were exposed to the mineralization solution for varying durations, resulting in a continuous gradient in the amount of calcium phosphate along the longitudinal axis. As shown by the SEM images in Figure 6B, the PLGA nanofiber mat showed a gradual transition in the amount of mineral deposition. At the basal edge (panel *i*, *d* = 0 mm), the nanofibers were densely coated with a thick layer of calcium phosphate. Moving away from the mineralized edge (panels *ii*, *iii*, and *iv*), the amount of calcium phosphate gradually decreased, leading to a transition from a mineral-rich to a polymer-dominant region. The mineral gradation resulted in a corresponding gradient in mechanical strength, making the mat potentially useful to replicate the transition in the native tendon-to-bone enthesis.

**Figure 6:**
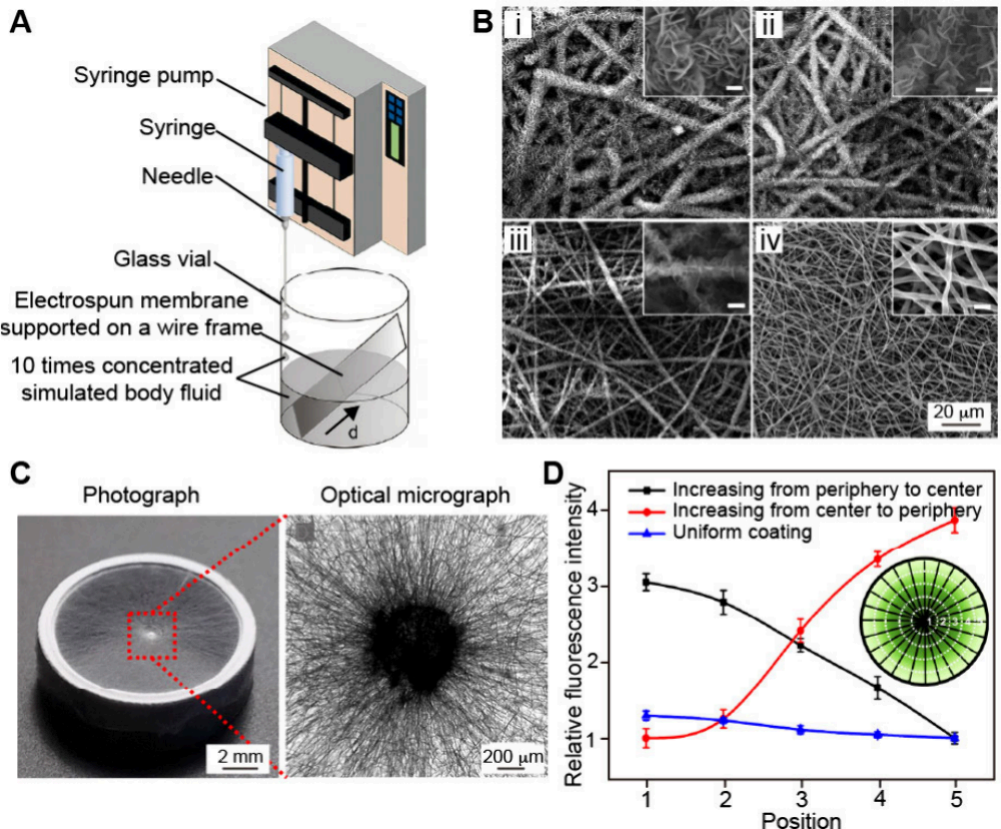
Fabrication of 1D and 2D gradients. (A) Schematic illustrating the fabrication of a graded coating of calcium phosphate on a plasma-treated nonwoven mat made of electrospun nanofibers. (B) SEM images of a nonwoven mat featuring graded coatings of calcium phosphate, captured at different distances from the bottom edge of the substrate: *i*) 0 mm, *ii*) 6 mm, *iii*) 9 mm, and *iv*) 11 mm. (C) Photograph and optical micrograph showing a mat of radially aligned PCL nanofibers. (D) Relative fluorescence intensity showing graded or uniform coating of FITC-BSA along the radially aligned nanofibers fabricated via BSA blocking. Inset: schematic showing the corresponding graded pattern. (A, B) Reproduced with permission from ref 35. Copyright 2009 American Chemical Society. (C, D) Reproduced with permission from ref 46. Copyright 2018 American Chemical Society.

In the second example, a 2D gradient of protein was constructed on a nanofiber mat using a center-elevated progressive immersion strategy combined with backfilling. As shown in Figure 6C, the electrospun PCL nanofiber mat exhibited a well-defined radial alignment, with the fibers oriented from the center toward the periphery.^46^ The nanofiber mat was plasma-treated and placed in the well of a 24-well plate with the central region elevated by 4 mm using a copper wire. A 0.1% BSA solution was then pumped into the well at a constant rate of 1 mL h^–1^. The setup yielded a 2D gradient of BSA, which was subsequently inverted by backfilling with a second bioactive protein into the bare regions. To evaluate the gradient, fluorescein isothiocyanate-labeled BSA (FITC-BSA) was employed as a model protein, enabling visualization of the pattern through fluorescence imaging. As shown in Figure 6D, fluorescence analysis indicates no significant variation in signal across the surface when the substrate was uniformly coated with FITC-BSA. However, when a gradient was created to increase from the center to the periphery (red line), the fluorescence intensity in the peripheral region was ca. 4-fold higher than that at the center. In contrast, when the gradient was reversed (black line), the fluorescence intensity decreased by ca. 3-fold from the center to the periphery. The findings demonstrated the effectiveness of the strategy in obtaining spatially graded protein distribution across the substrates. Altogether, gradient-based engineering holds promise for developing biomimetic scaffolds to guide cell migration and neurite extension in applications such as tissue regeneration.

### 2.2 Mask-Assisted Fabrication

In general, mask-assisted strategies rely on the use of a binary opaque and transparent, or grayscale, mask to spatially regulate the exposure of a substrate to the deposition of a substance or external stimuli such as ultraviolet (UV) irradiation. In this context, a mask refers to a material barrier capable of selectively blocking or allowing the passage of the substance or stimulus to the specific regions of a substrate. Depending on the type of external input, mask-assisted strategies can be broadly classified into two categories (Figure 7): *i*) electrospray-assisted deposition of particles, in which an opaque mask or aperture is used to control the deposition of micro- or nanoparticles onto the surface of a collector and *ii*) selective UV irradiation of a photosensitive substrate, where either a stationary grayscale mask or movable opaque mask is used to control the duration of UV exposure, thereby inducing graded chemical or structural transitions across the substrate. By tightly controlling the local exposure in terms of duration, intensity, or area, the mask-assisted strategy can be utilized to fabricate well-defined 1D and 2D surface gradients.

**Figure 7:**
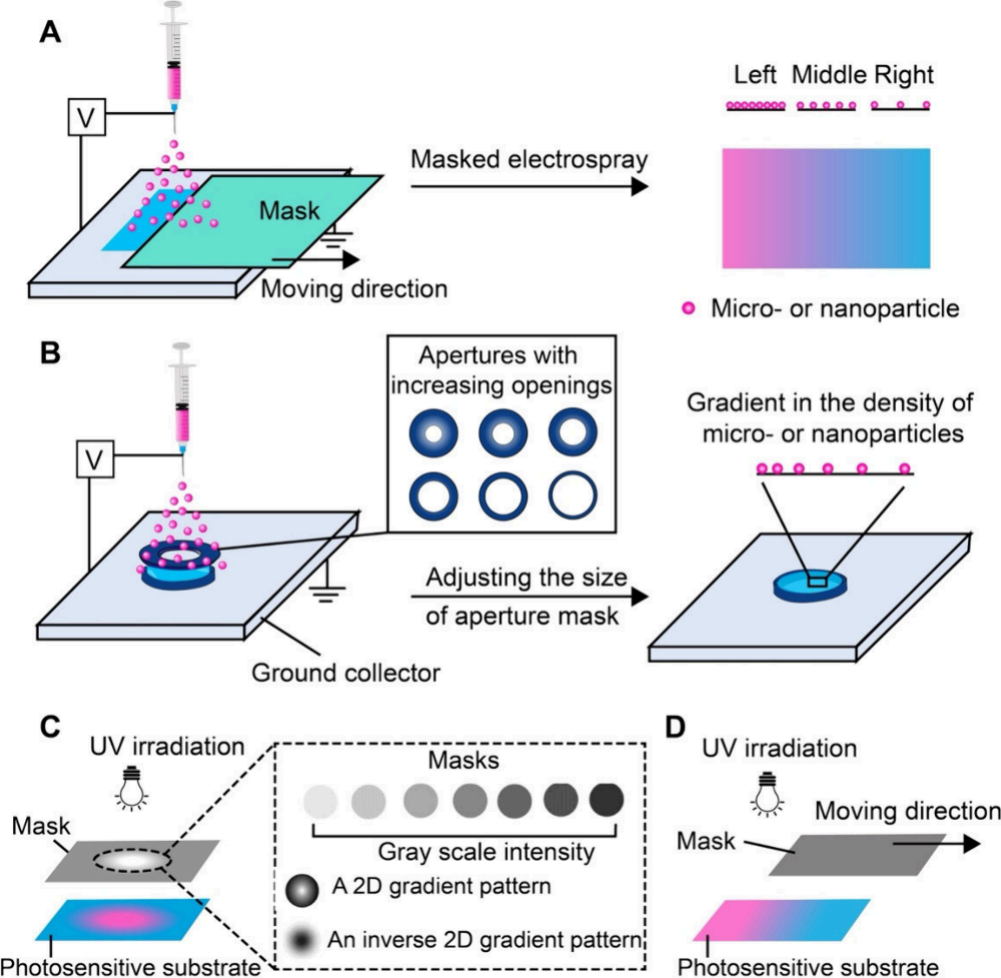
Schematics illustrating the fabrication of surface gradients using masks. (A, B) Fabrication of a surface gradient in the density of micro- or nanoparticles on a substrate by employing (A) a moving opaque mask or (B) a mask made of an opening-tunable aperture during electrospray. (C, D) Creation of a gradient on the surface of a photosensitive material through (C) the use of a grayscale mask or (D) by moving an opaque mask during the UV irradiation. (A, B) Reproduced with permission from ref 45. Copyright 2024 The Authors (CC BY 4.0). (C) Reproduced with permission from ref 53. Copyright 2003 American Chemical Society.

#### 2.2.1 Mask-Assisted Electrospray

Electrospray is an electrohydrodynamic atomization technique widely used for producing micro- or nanoparticles from a liquid solution containing polymers, biomacromolecules, and/or small-molecule drugs.^47^ A typical setup consists of a high-voltage power supply, a syringe pump, a metallic nozzle, and a grounded conductive collector. Upon applying a high voltage between the nozzle and the collector, a strong electric field is established, deforming the liquid at the nozzle tip into a funnel-shaped structure known as a Taylor cone.^48^ When the electrostatic force is strong enough to overcome surface tension, a charged liquid jet is ejected and then broken into fine, highly charged droplets due to Rayleigh instability. As the solvent evaporates during the jetting process, the droplets shrink and solidify into micro- or nanoparticles, which are then deposited on the collector.

A movable mask can be positioned between the nozzle and the collector to modulate the local deposition of particles, thereby creating a surface gradient, as illustrated in Figure 7A. As the mask moves linearly, different regions of the substrate undergo variations in deposition time, resulting in a 1D surface gradient in particle density. By varying the speed and pattern of moving, as well as the geometric shape of the mask, the profiles of gradient, such as continuous or stepwise, can be controlled accordingly. In contrast to the progressive immersion approach discussed in Section 2.1, mask-assisted electrospray offers superior spatial resolution and better control over the composition. The method also enables the deposition of particles on challenging substrates, such as those with nonwettable properties or complex topographies.^37^ Furthermore, it also allows the controlled delivery of multiple functional components, providing a versatile platform to construct well-defined surface gradients for various biomedical applications. In one study, biodegradable PLGA microparticles were deposited on a glass slide in a time-dependent manner using the electrospray system.^49^ With the assistance of a movable paper mask, the region of the substrate shielded for a longer period received fewer particles, whereas the more exposed region received a higher density of particles. By altering the direction and/or moving pattern of the mask, one could obtain reverse and bidirectional surface gradients of particles with controlled compositions.

In a somewhat different approach, an aperture mask with a tunable opening size was used to regulate the spatial deposition of particles (Figure 7B). Again, the aperture was positioned over the collector to block the deposition of particles in certain regions. The aperture was closed at the beginning to null any deposition of particles. As the aperture was gradually opened at a constant speed, particles were able to reach the surface of the collector. Since the center of the collector was exposed to the electrosprayed particles for the longest time, one expected a gradual decrease in particle density from the center toward the periphery. In one report, collagen nanoparticles were electrosprayed through a tunable aperture onto a mat of radially aligned electrospun PCL nanofibers, generating a 2D gradient in nanoparticle density.^50^ The graded surface embraced a combination of topographical cues and haptotactic cues to promote the migration of fibroblasts. Specifically, the graded distribution of collagen nanoparticles generated a radially decreasing adhesive signal, guiding cell adhesion and migration along the axis of the established polarity. The surface, which integrates both topographical and biochemical cues, could promote coordinated centripetal cell movement, holding promise for applications such as wound closure.

Electrosprayed particles can be further functionalized or preloaded with bioactive molecules, including growth factors, cytokines, and chemokines. Such modifications allow for the fabrication of multifunctional gradients that synergistically combine physical guidance cues with spatially defined biochemical signals.^51^ The resulting graded surfaces are anticipated to exhibit enhanced efficacy in modulating critical cellular behaviors, including adhesion, migration, and lineage-specific differentiation for diverse applications spanning from nerve regeneration to tendon-to-bone interface repair.

#### 2.2.2 Masked Irradiation of UV Light

Graded UV irradiation provides a versatile and straightforward approach for fabricating surface gradients through localized photochemical reactions.^52^ As shown in Figure 7C,D, this method relies on the ability to modulate the local UV dose across a surface, where variations in light intensity and/or exposure time are directly translated into spatially resolved photoreactions such as cross-linking, cleavage, or deprotection. The well-controlled reactions can produce spatial gradients in a range of surface properties, including cross-linking density, mechanical stiffness, surface hydrophilicity, and distribution of bioactive ligands such as adhesion peptides or proteins. Typically, graded UV irradiation can be achieved using two main approaches. The first involves the use of a stationary grayscale mask (Figure 7C), where light intensity varies across the mask due to changes in optical transparency.^53^ This approach allows for the creation of 1D, 2D, or complex gradient patterns, depending on the pattern of the mask. The second approach utilizes a moving opaque mask to mediate the exposure time across the surface of a substrate (Figure 7D). Both methods are effective in generating physicochemical gradients that can dictate diverse cellular processes in a spatially controlled manner.

In one study, Pham and co-workers fabricated polyacrylamide (PA) hydrogels with 2D gradients in stiffness via photopolymerization through a stationary grayscale mask.^53^ The mask was printed on transparency films with well-defined grayscale transitions from the center to the periphery, resulting in spatially graded UV doses across the substrate during gel cross-linking. As a result, the central region receiving the highest dose of UV irradiation became the most cross-linked and stiffest, whereas the peripheral areas remained the softest. When vascular smooth muscle cells were cultured on the hydrogel, they exhibited directional migration toward the center with the highest stiffness. In a different approach, Anseth and co-workers developed a 1D gradient in stiffness within a film of photodegradable poly­(ethylene glycol) (PEG)-based hydrogel using a moving opaque mask.^36^ The spatially controlled UV irradiation induced position-dependent photodegradation, yielding a 1D gradient in stiffness across the surface along the moving direction of the mask. The stiffness-graded surface was subsequently employed to investigate how valvular interstitial cells transform into myofibroblasts, a process crucial to the treatment of valve disease.

In general, the merits of a UV irradiation-induced method highly depend on the mask-related parameters, including the grayscale pattern and aperture geometry. For the technique involving a moving mask, the moving speed and direction of the mask also need to be tightly controlled.^36^ Both factors can directly influence the spatial distribution of UV dose, thereby influencing the uniformity and reproducibility of the resulting gradient. As a major advantage, UV-based methods do not require physical contact between the mask and the substrate, making them highly compatible with soft and delicate biomaterials used for guiding cell migration, alignment, or differentiation.

#### 2.2.3 Examples

To evaluate the quality and functionality of the surface gradients fabricated using mask-assisted methods, appropriate characterization methods are essential. Figure 8 shows two representative cases, one based on electrospray-based particle deposition (Figure 8A-C) and the other on selective UV-controlled hydrogel cross-linking (Figure 8D,E), for demonstrating the structural and functional features of the resultant gradients.

**Figure 8:**
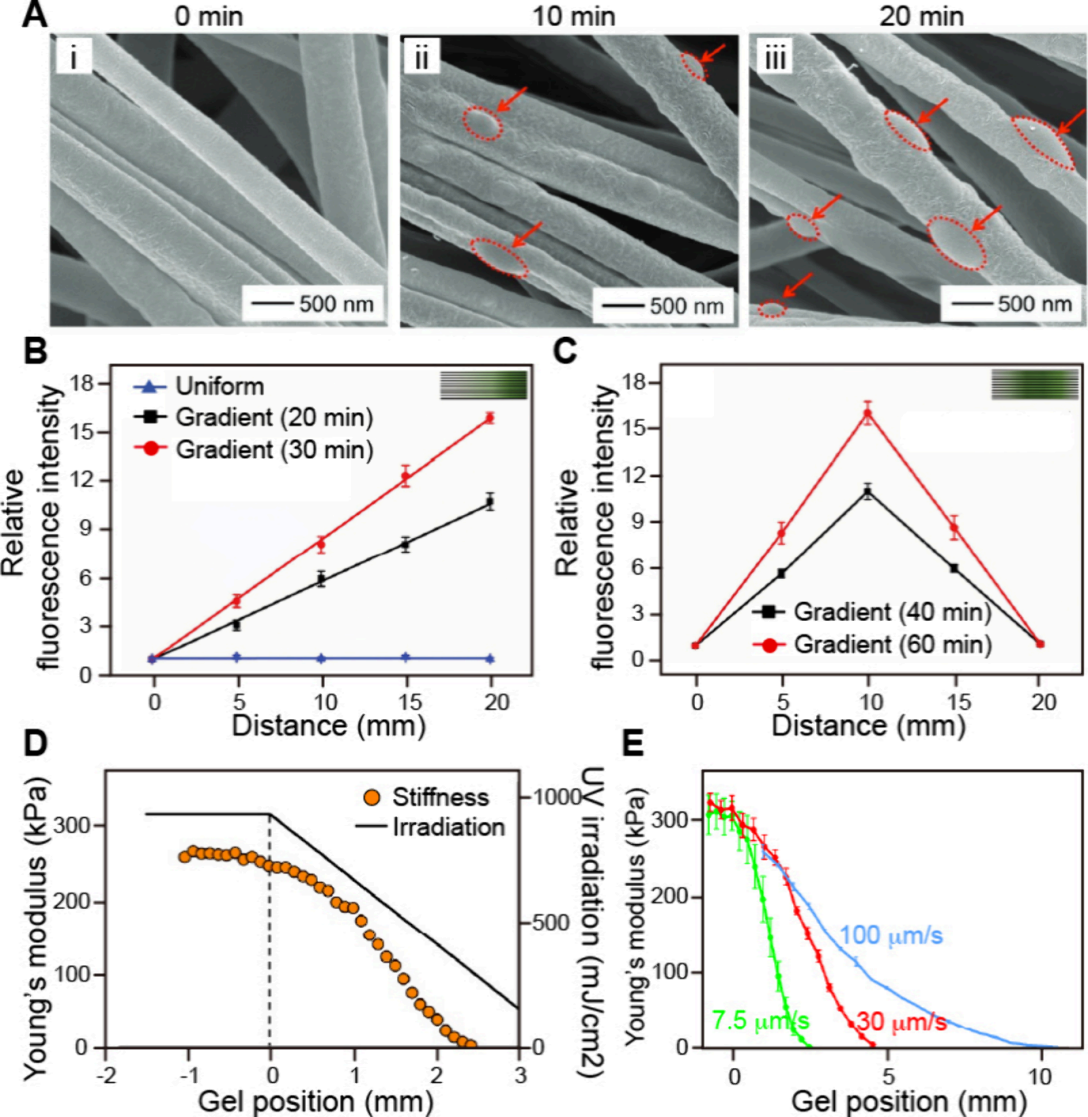
Examples of surface gradients fabricated through the assistance of a mask. (A) SEM images of uniaxially aligned PCL nanofibers whose surfaces were functionalized with collagen particles (marked by red dotted ellipses and arrows) at positions that correspond to collection for 0, 10, and 20 min, respectively. (B, C) Plots of relative fluorescence intensities of FITC-BSA-loaded collagen particles deposited on the aligned nanofibers. Insets in (B) and (C) show the corresponding gradient profiles. (D, E) Variations in hydrogel stiffness resulting from (D) a graded irradiation profile and (E) varying speed for the moving mask. At the beginning of fabrication, an opaque mask was positioned at *x* = 0 mm. For *x* < 0 in (D), the hydrogel received constant irradiation throughout the process. The moving speed of the mask in (D) was set at 15 μm s^–1^. (A–C) Reproduced with permission from ref 37. Copyright 2020 Wiley-VCH. (D, E) Reproduced with permission from ref 54. Copyright 2012 the Author(s) (CC BY 4.0).

In the electrospray-based system, collagen particles loaded with FITC-BSA were deposited on a mat of uniaxially aligned electrospun PCL nanofibers using a moving paper mask. Translation of the mask across the substrate established a 1D gradient in particle density along the fiber alignment.^37^ Figure 8A shows SEM images of the aligned nanofibers after being deposited with collagen particles exhibiting a gradient in density. Progressive translation of the mask at a speed of 0.1 cm min^–1^ resulted in a gradual decrease in deposition time, which in turn led to graded deposition of collagen particles along the nanofibers. At the site with the longest deposition (*iii*), one obtained the highest density of collagen particles (the red dotted ellipses). Since the particles contained some residual solvent, they became partially fused with the nanofiber surface without involving additional post-treatment. This work demonstrated the successful fabrication of a 1D gradient in particle density along the aligned fibers. Fluorescence microscopy was used to visualize the spatial distribution of FITC-BSA-labeled collagen particles, and the fluorescence intensity was subsequently analyzed to quantify the gradient profile across the nanofiber mat. Figure 8B shows the relative fluorescence intensity along the aligned fibers for the samples prepared with uniform or graded deposition time. A slower moving speed of 0.067 cm min^–1^ led to a steeper gradient, as reflected by a greater difference in intensity between the two ends of the mat (red line). This result demonstrates that the steepness of the gradient could be tuned by adjusting the moving speed of the mask. Figure 8C shows a bidirectional gradient, with fluorescence intensity increasing from both edges toward the center along the aligned fibers. In addition to continuous patterns, stepwise gradients can also be fabricated by controlling discrete shifts in the mask movement, highlighting the versatility of this method in tailoring the spatially graded distribution profile. The gradients in particle density were able to affect cell behaviors such as stem cell migration, fibroblast centripetal movement, and neurite extension from dorsal root ganglion (DRG) bodies along the aligned nanofibers.

For the UV-based hydrogel system, a well-defined gradient in stiffness was established by photopolymerizing the acrylamide/bis-acrylamide precursor solution under spatially modulated UV irradiation.^54^ The process was achieved by moving an opaque mask across the substrate at a constant speed, producing a 1D gradient in terms of UV exposure time. As a result, the hydrogel experienced position-dependent cross-linking, creating a 1D gradient in stiffness along the moving direction of the mask. To characterize the gradient in stiffness, atomic force microscopy (AFM) analysis was conducted along the moving direction of the mask (Figure 8D). The mask initially covered the region from *x* = 0 (marked by a dashed line) to *x* = 3.0 mm. In comparison, the area left to *x* = 0 mm was fully exposed to UV irradiation, leading to uniform cross-linking and constant Young’s modulus. When the mask was moved at a speed of 15 μm s^–1^, the substrate over the region from *x* = 0 to 2.4 mm showed a progressive decrease in cross-linking density, leading to a corresponding gradient in Young’s modulus. By adjusting the moving speed of the mask (Figure 8E), the slope of the gradient profile could be controlled while maintaining a constant range of stiffness. When cells were incubated on the resulting graded hydrogel, their spreading exhibited a linear correlation with the stiffness of the hydrogel, demonstrating the controlled mechanical modulation of cellular behavior.

Taken together, the above studies demonstrate that mask-assisted strategies can be used to create surface gradients in terms of both structural and mechanical properties. Such gradients can be engineered to regulate a range of cell behaviors, advancing their applicability in tissue engineering and regenerative medicine.

### 2.3 Field-Induced Fabrication

Field-induced methods offer a versatile strategy for fabricating functionally graded surfaces by controlling the spatial distribution of particles, molecules, or bioactive agents using external physical stimuli. Unlike conventional approaches that rely on chemical patterning or mechanical structuring, these methods utilize an external field, such as a magnetic, electric, or thermal field. The techniques exploit the responsiveness of materials to external forces, enabling tight controls over the position, density, and organization of surface features. Moreover, field-induced methods allow dynamic, noncontact manipulation of materials, making them attractive for constructing gradients in a flexible and scalable manner. In this section, we focus on such methods that utilize magnetic (Figure 9A), electric (Figure 9B), and temperature fields (Figure 9C), respectively.

**Figure 9:**
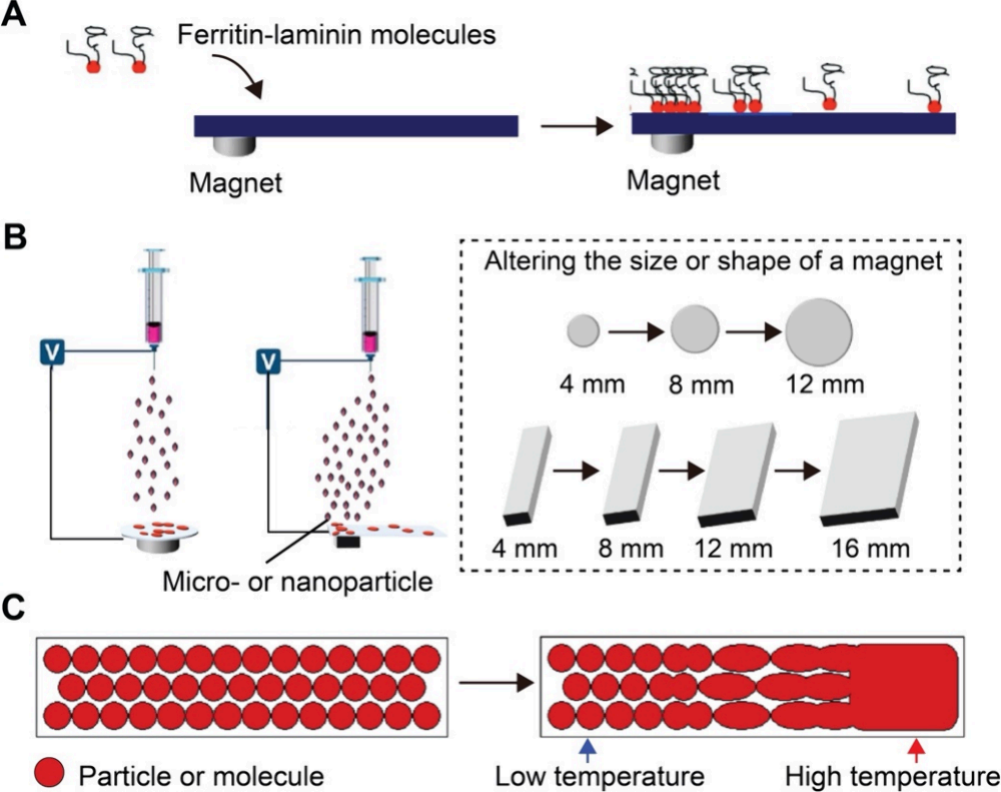
Preparation of surface gradients induced by an external field. (A) Ferritin-conjugated laminin forms a graded distribution on a substrate exposed to a magnet. (B) Creation of 1D or 2D gradients of bioactive proteins, as well as micro- or nanoparticles, increasing from the periphery to the center, was achieved using a magnet to modulate the distribution of the electrostatic field during electrospray. (C) A surface coated with uniform PS microspheres is subjected to a lateral temperature gradient, which induces progressive deformation and fusion of the particles toward the heated region as a result of thermally activated structural changes. (A) Reproduced with permission from ref 57. Copyright 2021 Wiley-VCH. (B) Reproduced with permission ref 69. Copyright 2021 Elsevier. (C) Reproduced with permission ref 73. Copyright 2005 American Chemical Society.

#### 2.3.1 Magnetic Field

Magnetic field-induced surface gradients are formed by leveraging the responsiveness of magnetic or magnetically functionalized particles to an external magnetic field.^55^ When exposed to a nonuniform magnetic field, the particles are driven by magnetic force to migrate toward regions of higher field intensity to minimize their magnetic potential energy.^56^ This method allows for noncontact and controllable construction of surface gradients.

In a typical process, a permanent magnet is placed beneath the substrate during deposition. A spatially varying magnetic field beneath the substrate causes the particles to preferentially accumulate in regions of higher field intensity, generating a surface gradient.^57^ By adjusting the size, shape, or position of the magnet, the steepness and spatial extent of the resulting gradient can be tuned. This strategy can be applied to both colloidal particles and biological samples. Figure 9A shows an example where laminin, an ECM protein, was conjugated to ferritin to generate a magnetically active protein complex.^57,58^ When deposited on a surface in the presence of a magnetic field, the laminin-ferritin complexes migrated to the site with the greatest magnetic field strength, creating a 2D gradient in laminin concentration. This work demonstrates that magnetic fields can be directly used to control the spatial organization of bioactive molecules in a controlled manner.

Magnetic fields have also been used to guide the spatial positioning of cells. Levy and co-workers demonstrated a magnetically guided method to create a gradient in cell density on the surface of a vascular stent using magnetically labeled endothelial cells.^59^ Specifically, bovine aortic endothelial cells were preloaded with superparamagnetic iron oxide nanoparticles (SPIONs) and then introduced into a culture system containing 304-grade stainless-steel stents. Applying a magnetic field across the stents would induce high local magnetic field gradients along the metallic stent struts. The gradient was formed because the metallic mesh structure of the stent acted to concentrate magnetic flux lines, especially near the narrow edges and curved surface, effectively creating a 2D gradient in the field around the cylindrical stent. As a result, magnetically responsive endothelial cells were selectively attracted to and immobilized on specific regions along the stent. This approach allowed for the direct modulation of cell localization, offering a promising strategy for engineering biofunctional vascular implants capable of promoting re-endothelialization while minimizing restenosis. Similarly, Chen and co-workers developed a system where a stable gradient of endothelial cells labeled with SPIONs was formed in fibronectin-coated plate wells.^60^ By placing a neodymium magnet underneath the culture plate, the SPION-labeled cells were directed to migrate toward the area with a higher magnetic field intensity. This approach combines the adhesive properties of fibronectin matrix with the noninvasive, real-time control of cell localization through the assistance of an external magnetic field.

Overall, the magnetic field offers a straightforward and effective means for controlling the spatial distribution of colloidal particles, bioactive molecules, and cells.^61,62^ The approach allows precise manipulation without physical contact, reducing contamination while preserving material integrity. By adjusting the parameters of the magnetic field and the properties of magnetically responsive components, various types of gradients can be fabricated on a variety of surfaces. The techniques extend beyond simple material deposition to enable the creation of graded biological patterns, offering strategies for tissue engineering, surface biofunctionalization, and related applications.

#### 2.3.2 Electric Field

Similar spatial control can also be achieved through the application of an electric field. In particular, electrostatic forces can act on materials with inherent or induced charges, directing their motion toward the region of opposite polarity.^63,64^ The migration can be exploited to concentrate materials in specific areas of a collector, with the final gradient determined by the electric field pattern and the duration of application.

An electric field has been used to direct the deposition of charged particles or droplets during electrospray.^65^ When a high-voltage electric field is applied between the nozzle and collector, charged species are propelled along the field lines.^66^ The key to generating spatial gradients lies in manipulating the distribution of the electric field to control where particles accumulate and thus their surface density. Several methods exist for tuning this distribution, including modifying the collector geometry, adjusting the placement and orientation of the collector, and incorporating magnetic components to alter the local field intensity.^67–69^ In particular, magnetic elements can be strategically placed beneath the substrate not to attract particles directly but to distort the electric field locally, thereby concentrating their deposition in targeted regions.

As illustrated in Figure 9B, Wu and co-workers demonstrated this principle using a magnet-modulated electrospray setup, where circular magnets of increasing diameter were sequentially placed under the substrate to enhance the electric field strength across a broader region.^69^ The manipulation of the field resulted in a 2D gradient of fibronectin from the outer edge toward the center of the substrate. Similarly, 1D gradients of laminin were achieved by arranging the electric field laterally across a glass slide using elongated magnets as electric field modulators. The magnet-modulated electrospray technique was demonstrated with both proteins and polymer microparticles, achieving continuous gradients in particle density and thus graded distributions of bioactive protein density. Notably, the resulting gradients were able to influence cell migration: increasing fibronectin or laminin density toward one end of the gradient could direct and enhance cell movement.

The versatility of electric-field-mediated deposition, coupled with field-modulating elements like magnets, offers a powerful strategy for fabricating graded biointerfaces for tissue engineering and regenerative medicine.

#### 2.3.3 Temperature Gradient

Gradients can also be generated by applying a temperature gradient across the surface of a substrate during or after the deposition of functional molecules or particles. Many polymers, proteins, and colloidal particles exhibit temperature-dependent behavior, such as changes in solubility, mobility, and/or adhesion.^70–72^ By subjecting one end of a coated substrate to an elevated temperature while keeping the other end at a lower temperature, the thermally responsive components will be directed to create a gradient. This approach is commonly used to generate a gradual change in surface morphology by applying a temperature gradient along a polymer substrate to induce variation in polymer chain mobility. As shown in Figure 9C, Han and co-workers demonstrated the concept using a film of polystyrene (PS) microspheres spin-coated on a silicon wafer.^73^ A temperature gradient was created by heating one end of the substrate to 130 °C while keeping the opposite end at room temperature under a nitrogen atmosphere. Over a period of 48 h, the temperature gradient induced a graded transformation in the film’s microstructure. The root of the transformation lies in the behavior of PS chains relative to the glass transition temperature (Tg). On the unheated side, where the temperature was well below Tg, the polymer chains remained frozen and rigid, preserving the original microsphere geometry. As the local temperature surpassed Tg toward the heated end, the chains gained limited mobility, causing partial deformation and fusion between adjacent beads, resulting in a semiflattened structure. At the heated end, where the temperature was well above Tg, the chains became fully mobile, allowing the microspheres to coalesce and form a smooth, continuous film.

AFM images (Figure 10A) revealed a progressive change in topography from a rough surface on the unheated side to a smooth surface on the heated side. Quantitative analysis of surface roughness (Figure 10B) showed that the root-mean-square (RMS) roughness decreased from 46.3 nm at the cold end to 14.6 nm at the hot end. The topographical change directly impacted surface wettability: the water contact angle decreased from 148.1° to 88.7° along the gradient (Figure 10C). The method is particularly advantageous for modifying a thermoplastic surface, allowing for the formation of spatial gradients in roughness and interfacial energy without requiring chemical modification or complex patterning tools. It is applicable in contexts such as droplet transport, modulation of cell adhesion, and tuning of surface energy for microfluidic or diagnostic devices.

**Figure 10:**
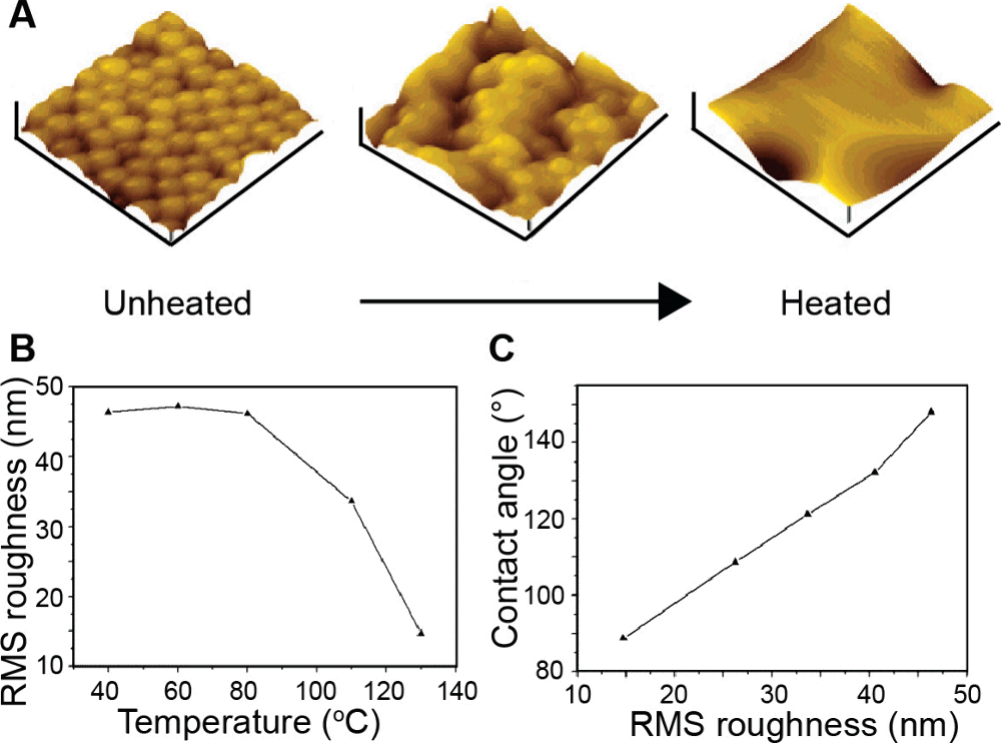
Creation of a topographical gradient by subjecting a film of PS microspheres to a temperature gradient. (A) AFM images captured at various locations across the film, spanning from the unheated end to the heated end. (B) Relationship between RMS roughness and temperature. (C) Relationship between water contact angle and RMS roughness. Reproduced with permission from ref 73. Copyright 2005 American Chemical Society.

### 2.4 Fabrication Enabled by Microfluidics

Microfluidic technology allows the tight control of fluids at the micrometer scale and has become a versatile tool for fabricating chemical and material gradients.^74^ The microfluidic devices leverage deterministic laminar flow and controlled molecular diffusion to create gradients with high spatial and temporal resolutions.^75^ Compared to conventional methods such as manual pipetting or gel-based diffusion, microfluidic techniques offer good reproducibility, minimal reagent consumption, and the ability to integrate multiple functions within a compact chip platform.^76–78^

Gradients are formed by introducing multiple fluid streams with distinct solute concentrations into a shared microchannel. Under laminar flow, the fluid streams do not mix but remain adjacent instead, allowing the solutes to diffuse at the interfaces to create a continuous gradient.^79^ The gradient profile, whether linear, sigmoidal, or asymmetric, is determined by channel geometry, relative flow rates, solute diffusion coefficients, and channel length.^80^ A standard design is the Y-junction gradient generator, where two inlets carrying different solutions merge into a single channel. The configuration allows the formation of stable lateral gradients across the channel width through interstream diffusion.^81,82^ In one example, a Y-shaped microfluidic device was used to generate a 1D gradient of a chemoattractant across a microchannel for quantifying bacterial chemotaxis.^83^ Significantly, the gradient profile can be modulated by adjusting the relative inlet flow rates: equal flows yield a symmetric gradient, whereas imbalanced flows skew the profile toward one side. Such devices have been actively explored for applications like chemotaxis assays, cell migration studies, and controlled material deposition, offering a robust and tunable platform for generating surface-bound or volumetric gradients in a reproducible and scalable manner.^84–86^

Serpentine channel designs significantly affect gradient formation by enhancing transverse mixing between the parallel, laminar streams. The repeated bends in the channel generate Dean vortices—secondary flows that arise due to centrifugal forces acting on the fluid, leading to increased interfacial contact between solute streams.^87^ As a result, solute diffusion across adjacent streams is accelerated, enabling the formation of a steeper and more uniform gradient over a shorter flow distance.^88,89^ The improved mixing is directly related to faster gradient formation, particularly beneficial for creating sharper or more complex gradient profiles within compact microfluidic devices. The enhanced mixing dynamics also allow for fine-tuned control over gradient steepness and spatial resolution, making serpentine mixers especially useful in high-throughput screening and dynamic cell signaling studies.^90^

Beyond serpentine channels, more sophisticated gradient generators utilizing cascaded branching structures, often referred to as “Christmas tree” designs, were also developed to produce surface gradients. Such “Christmas tree” generators progressively mix solutes through sequential splitting and recombination, enabling a tight control over the gradient profile. As illustrated in Figure 11A, two inlet solutions are merged and then progressively diluted through multiple bifurcating stages.^91^ At each stage, partially mixed streams are recombined with fresh input, producing a series of intermediate concentrations. The streams are then delivered in parallel to a downstream surface or chamber, creating a continuous gradient across the substrate. The architecture allows for exceptional precision and reproducibility in the gradient profile. In one study, Whitesides and co-workers utilized a tree-like microfluidic network to generate a 1D gradient across a large chamber for cell culture.^92^ Moreover, as shown in Figure 11B, expanding the number of input channels enables more complex surface gradients. For example, introducing a third input stream yields peaked or asymmetric profiles. Altering the concentration in one inlet or the ratio of flow rates alters the resulting gradient profile by shifting its midpoint or changing its slope.

**Figure 11:**
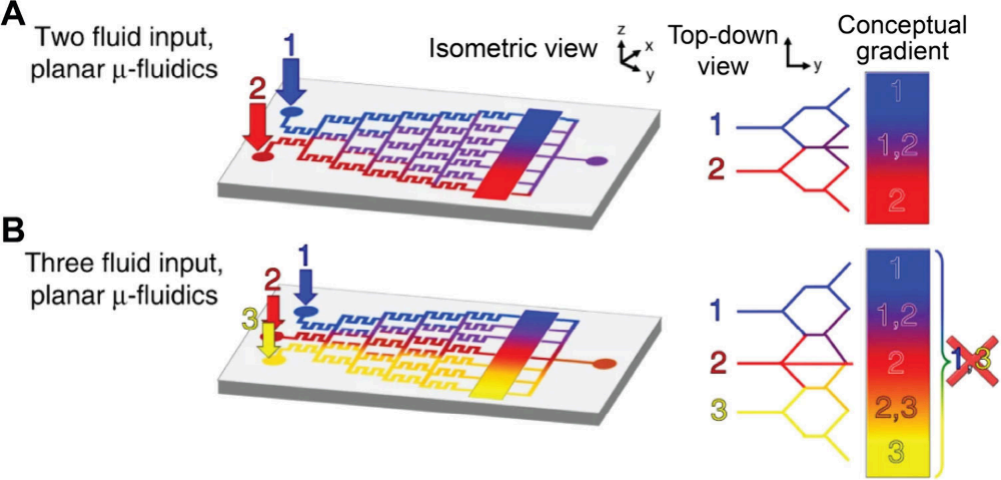
Creation of a gradient using a microfluidic device. (A, B) Schematic of the (A) two-input and (B) three-input planar devices with “Christmas tree” designs, for producing 1D gradients. Reproduced with permission from ref 91. Copyright 2020 the Author(s) (CC BY 4.0).

In addition to solution-phase gradients, microfluidic methods have also been combined with materials processing techniques to fabricate a functionally graded surface. Qin and co-workers utilized a two-inlet microfluidic device coupled with an electrospinning process to create nanofiber mats with a graded surface.^93^ As shown in Figure 12A, gelatin and PLGA solutions were fed at controlled rates into a microfluidic Y-junction that merged into a single outlet. A compositional gradient was formed in the resulting blended solution stream by the time it reached the outlet, which was connected to a spinneret for electrospinning. The jet with a different mixing ratio between the two polymer solutions was then deposited on a moving collector to form a nonwoven fibrous mat. Using this method, compositional gradients were formed in the nanofibers by varying the ratio of gelatin to PLGA. As shown in Figure 12B, SEM images showed significant differences in fiber morphology depending on the polymer ratio, transitioning from thick, membrane-bridged, gelatin-rich fibers to thinner, PLGA-dominated fibers. Elemental analysis (Figure 12C) was used to determine the carbon-to-oxygen ratio in the fibers. Beyond composition, the platform was also employed to create gradients in the concentration of bioactive molecules. Fluorescent dyes of vastly different molecular weights, including Rhodamine B (479 Da) and human IgG-FITC (160 000 Da), were successfully coelectrospun with the fibers, creating a 1D gradient in fluorescence intensity (Figure 12D). The authors also demonstrated that mesenchymal stem cells (MSCs) cultured on the fiber scaffold showed specific differentiation corresponding to the underlying biomolecular gradient.

**Figure 12:**
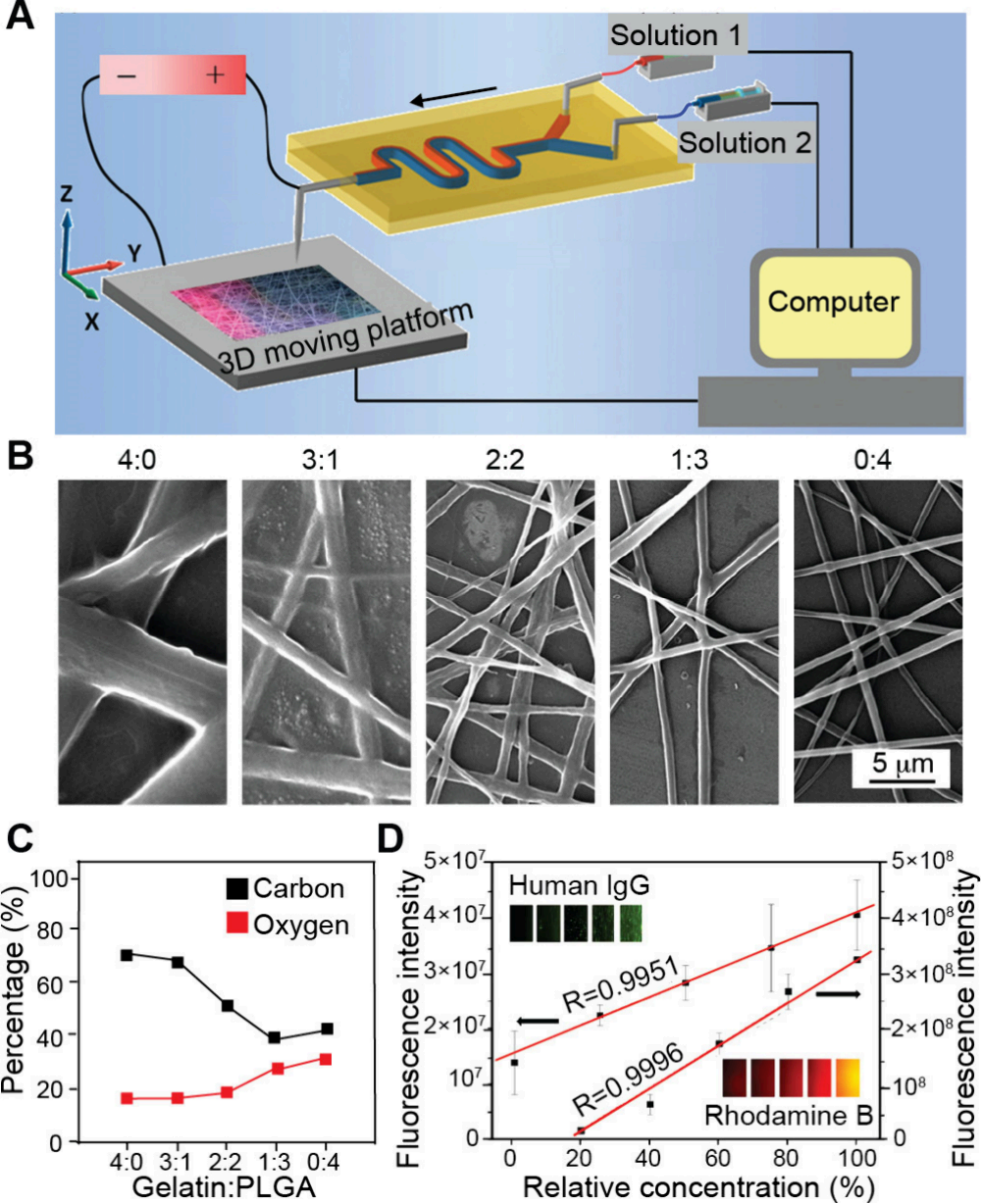
Fabrication of graded nanofiber mats using a microfluidic device. (A) Schematic of the microfluidic setup for generating nanofiber mats with tunable gradient profiles using two inlets to supply gelatin and PLGA solutions at controlled ratios. (B, C) SEM images of nanofiber mats fabricated with different gelatin-to-PLGA ratios, alongside elemental analysis showing the carbon-to-oxygen ratio in the fiber mats. (D) Characterization of the biochemical gradient in the nanofiber mats using Rhodamine B and Human IgG-FITC. Fluorescence intensity of the nanofiber mats as a function of the relative concentrations of Rhodamine B and Human IgG, illustrating their graded distribution within the fibers. Reproduced with permission from ref 93. Copyright 2012 American Chemical Society.

However, major challenges still hinder the direct integration of cells with microfluidic assays, including high operational costs, lengthy experimental procedures, and the reliance on continuous pumping systems to maintain stable chemical gradients and sustain cell viability over time. The dependence on external equipment complicates the practicality of conventional microfluidic platforms for biological applications. As such, Javanmard and co-workers reported a stand-alone microfluidic gradient chip for cancer biology studies that did not require continuous pumping.^94^ The device could produce linear or polynomial gradients in a cell culture chamber by diffusion through hydrogel barriers, and it was used to investigate cancer cell invasion under the gradient of chemokine. By eliminating external pumps and directly utilizing a reusable microfluidic insert on a culture dish, one enhances the ease of use while maintaining a fine control over the gradient profile, advancing the integration of microfluidic technology with biomedical assays.

In summary, microfluidic devices for creating gradients have evolved from simple dual-stream mixers that produce basic 1D gradients to sophisticated platforms that offer tunable profiles, higher complexity (e.g., multiple inputs, nonlinear gradients), and integration with standard cell culture workflows. The ability to precisely engineer surface gradients has created opportunities in biomedicine by enabling tight controls over cell migration and alignment, the design of graded scaffolds for regenerative therapies, and the recreation of pathological gradients for *in vitro* disease models. Ongoing improvements in microfluidic design, such as 3D-printed gradient networks and dynamic gradient control, are expected to expand the toolkit for developing functionally graded surfaces and expanding their biomedical applications.

Taken together, a detailed summary of these surface-based strategies, such as their control strategy, achievable gradient, resolution, scalability, and cost implications, is provided in Table 1.

**Table 1:** Overview of Fabrication Methods for Functionally-Graded Surfaces^a^

Technique	Control Strategy	Achievable Gradient	Gradient Control	Setup Complexity	Res./Cost/Rep./Scal.	Major Biomedical Applications	Ref.
Progressive immersion	Physical adsorption, electrostatic attraction, chemical reaction	Composition, cell density, mechanical properties	**Moderate**: Solution or suspension concentration; injection rate; substrate tilt angle; temperature.	**Good**: Compatible with a wide range of materials and does not require specialized equipment.	2/5/2/4	Tissue engineering scaffolds; guiding cell adhesion, migration, and differentiation.	^35^,^38^,^40^,^42^,^43^,^46^
	Vapor-induced welding	Structure (porosity, fiber architecture)	**Moderate**: Solvent concentration; exposure time; solvent volatility.	**Good**: Simple method that eliminates the need for complex instrumentation or patterning masks.	2/5/2/4	Tissue engineering scaffolds; guiding cell migration and alignment.	^44^
Mask-assisted fabrication	Electrospray	Composition, particle density	**Good**: Moving speed and pattern of the mask; geometric shape of the mask or aperture.	**Moderate**: Requires a specialized electrospray setup.	2/3/3/3	Guiding cell migration and neurite extension; wound healing; interfacial tissue repair.	^37^,^49^
	Masked UV light irradiation	Composition, cross-linking density, mechanical properties, hydrophilicity	**Good**: Grayscale pattern and aperture geometry of a stationary mask or the moving speed and direction of an opaque mask.	**Moderate**: Requires a UV light source and the fabrication of grayscale or opaque masks.	3/2/4/3	Guiding cell migration; investigating mechanotransduction and cell differentiation.	^36^,^53^,^54^
Field-induced fabrication	Magnetic field	Composition, cell density	**Good**: Size, shape, position, and intensity of the magnet; the properties of the magnetically responsive components.	**Moderate**: Requires the use of permanent magnets or electromagnets.	3/4/3/3	Engineering biofunctional vascular implants; surface biofunctionalization.	^58–62^
	Electric field	Composition	**Good**: Electric field intensity and duration; collector geometry and placement; field-modulating elements.	**Moderate**: Requires a high-voltage power supply and electrodes.	3/3/3/2	Guiding cell migration; interfacial tissue repair.	^69^
	Temperature field	Surface morphology, roughness, interfacial energy	**Moderate**: Temperature differential across the substrate.	**Good**: Can be achieved with a simple heat source and sink.	2/5/1/4	Modulating cell adhesion; tuning surface energy for microfluidic or diagnostic devices.	^73^
Microfluidics-enabled fabrication	Stand-alone microfluidic system	Composition	**Good**: Channel geometry (e.g., Y-junction, serpentine); relative flow rates; solute diffusion coefficients; channel length.	**Low**: Requires specialized equipment and expertise for microfluidic chip design and fabrication.	5/1/5/1	Chemotaxis assays; cell migration studies; controlled material deposition.	^90–93^

a Res. (Resolution): 5 = Excellent (e.g., < 10 μm); 1 = Poor (e.g., > 1 mm). Cost: 5 = Very Low; 1 = Very High. Rep. (Reproducibility): 5 = Excellent; 1 = Poor. Scal. (Scalability): 5 = Excellent; 1 = Poor

## 3 Fabrication of Functionally Graded Materials

Functionally graded materials exhibit gradients in the bulk rather than on the surface. Achieving spatial gradients in the bulk of a material presents major challenges due to the complexity in establishing internal gradients throughout the 3D volume. For example, one needs to overcome volumetric mass transport/diffusion limitations and maintain structural/chemical stability in the entire bulk during fabrication. In this section, we focus on three different strategies used for the fabrication of functionally graded materials: diffusion, force-driven movement, and layer-by-layer fabrication.

### 3.1 Diffusion

Diffusion provides an effective means for fabricating functionally graded materials by harnessing spontaneous molecular redistribution at an interface along the thermodynamic driving force, creating gradients in composition and properties. Diffusion naturally leads to a gradual change in concentration, as governed by either Fickian diffusion (driven by a gradient in concentration) or a non-Fickian process (e.g., solvent–polymer interactions),^95^ offering the advantages of a predictable spatiotemporal evolution of gradients. By controlling variable parameters such as diffusion time, temperature, boundary conditions, and matrix properties (e.g., polymer mesh size), one can obtain gradients from the nanoscale to the macroscopic scale. Diffusion creates gradients through natural physicochemical equilibria, circumventing disruptive mechanical or thermal interventions. The property, combined with its scalability and ambient processing compatibility, ensures both material integrity and functionally optimized transitions, making it industrially viable, biologically congruent, and uniquely suited for biomedical applications.

#### 3.1.1 Diffusion of Molecules or Nanoparticles

Controlling molecular diffusion in a matrix to establish spatiotemporal gradients is an appealing method for fabricating graded biomaterials. The principle operates based on Fick’s laws of diffusion, where the net molecular flux from regions of high concentration to regions of low concentration drives the formation of a gradient over time and space.^96^ In one demonstration, Choi and co-workers fabricated polyacrylamide-acrylamide (PAA) hydrogels with graded stiffness by controlling the diffusion of unreacted cross-linker and monomer into a prepolymerized hydrogel sink.^97^ Briefly, acrylamide monomers and N,N′-methylenebis­(acrylamide) cross-linkers were poured into a glass mold and covered with a glass coverslip with an angled ramp of 3° in the vertical plane. In this case, the polymerization of the precursor resulted in the formation of a PA hydrogel with a wedge-shaped structure. Then, a second solution containing acrylamide precursor at different concentrations was incubated with the first PA gel, during which the precursor gradually diffused into the hydrogel. Because the thinnest areas of the second hydrogel component suffered the greatest proportional loss of monomers/cross-linker, a gradient in stiffness was formed. After polymerization of the second component, the PAA gel composed of two sequentially polymerized, inversely oriented, ramp-shaped components was obtained (Figure 13A,B). The Young’s modulus of the gel parallel to the ramp axis measured by AFM confirmed the formation of a gradient in stiffness (Figure 13C). The mechanism responsible for creating the gradient also suggested an accompanying pore-size gradient along the hydrogel surface, as higher degrees of cross-linking would result in smaller pore sizes in the stiffer regions (Figure 13D). Cryo-SEM images demonstrated a gradient in pore size across the surface of the PAA gel (Figure 13E), with pore sizes gradually decreasing from 11 ± 5.2 μm in low-stiffness regions to 4.1 ± 0.4 μm in the high-stiffness areas. The gradient of stiffness in the hydrogel led to distinct cell responses, including variations in cell morphology, migration, and differentiation.

**Figure 13:**
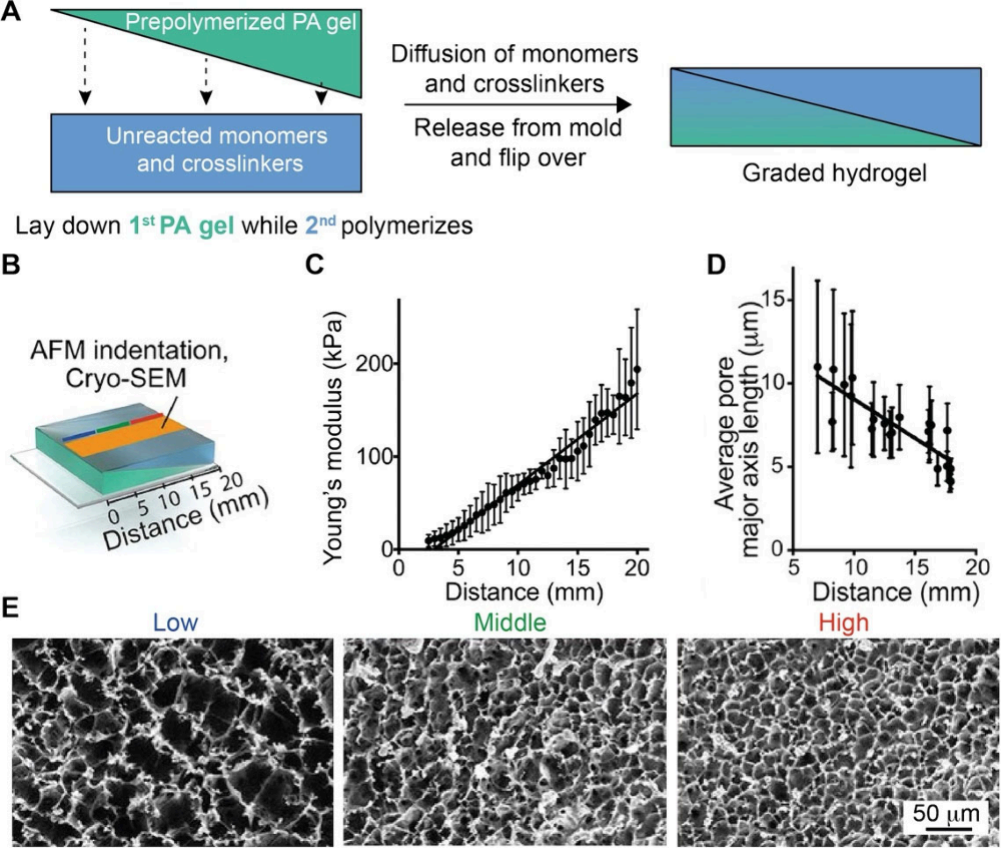
Fabrication of a hydrogel with gradients in porosity and stiffness based on molecular diffusion. (A) Schematic illustration of the double polymerization process for the fabrication of a graded hydrogel. (B) Schematic of the graded hydrogel, with three zones of different porosities marked out by blue, green, and red along the direction parallel to the ramp. (C) Young’s modulus and (D) pore size across the surface of the hydrogel. (E) Cryo-SEM images of a graded hydrogel at low (blue), middle (green), and high (red) stiffness ranges. Reproduced with permission from ref 97. Copyright 2025 National Academy of Sciences.

Nanoparticles also undergo diffusion in the matrix from regions of high concentrations to low concentrations, creating a graded distribution.^98,99^ In one study, Xia and co-workers fabricated a graded hydroxyapatite/PCL (HAp/PCL) scaffold with a gradient in HAp content by leveraging both the diffusion of HAp nanoparticles and PCL polymer chains in a swollen matrix (Figure 14A).^100^ The process started with the preparation of a HAp/PCL (with the mass ratio of HAp to PCL fixed at 1:1) composite film by solution casting. A PCL solution in 1,4-dioxane was then introduced onto the top of the film to swell the HAp/PCL composite, followed by evaporation overnight to remove the solvent. During the process of swelling, some of the PCL polymer chains diffused into the HAp/PCL composite with the solvent, and the HAp nanoparticles in the swollen HAp/PCL composite moved to the interface, creating a gradient in mineral content at the interface between the composite film and the polymer solution. Upon removal of the solvent, the PCL remaining in the solution was deposited as a thin layer made of pure PCL on top of the composite layer. The final sample exhibited a sandwich structure comprised of a PCL region on the top, a HAp-graded zone in the middle, and a HAp/PCL composite layer with a fixed HAp content at the bottom. Laser micromachining was then used to create an array of funnel-shaped channels, with an opening ca. 200 μm in diameter at the top and a center-to-center separation of ca.100 μm, to facilitate the seeding and migration of cells (Figure 14B).

**Figure 14:**
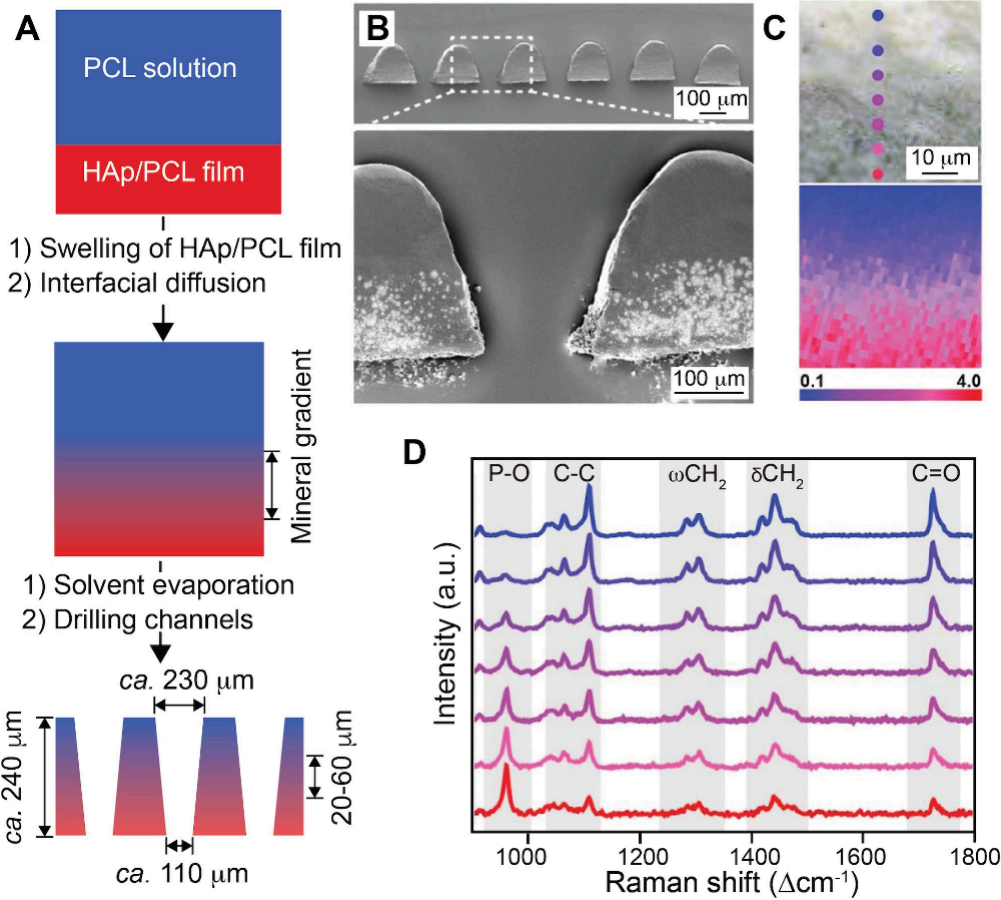
Fabrication and characterizations of a polymer scaffold with a continuous gradient in mineral content. (A) Schematic showing the fabrication process, where the gradient in HAp content is formed as a result of swelling-induced interfacial diffusion. (B) Cross-sectional SEM images of a HAp-graded scaffold with the channels being drilled using a CO_2_ laser. (C) Raman mapping of the HAp distribution in the graded region. The color correlates to the intensity ratio between the peaks at 960 cm^–1^ (P–O stretch) and 1724 cm^–1^ (C=O stretch), which correspond to HAp and PCL, respectively. (D) Representative Raman spectra recorded from different sites are indicated in (C). Reproduced with permission from ref 100. Copyright 2021 Wiley-VCH.

The distribution of HAp nanoparticles along the thickness direction of the film was characterized using Raman microscopy. The relative HAp content in each region was correlated to the ratio of the Raman shift intensity at 960 cm^–1^ to that at 1724 cm^–1^, corresponding to the P–O stretching of HAp and the C = O stretching of PCL, respectively. The Raman spectra recorded from the regions of interest, as indicated in Figure 14C by color dots, are displayed in Figure 14D.^100^ In the transition zone, the Raman shift intensity at 960 cm^–1^ (P–O stretching of HAp) gradually increased from the upper part to the lower part along the vertical direction, while the Raman shift intensities at 1031–1109 cm^–1^ (C–C stretch), 1281–1306 cm^–1^ (CH_2_ bend), 1418–1474 cm^–1^ (CH_2_ twist), and 1724 cm^–1^ (C = O stretch) for PCL gradually decreased, demonstrating the graded distribution of HAp. Since the formation of a gradient in HAp content in the transition zone was caused by swelling-induced diffusion, the length scale of the gradient was directly related to the extent of swelling for the HAp/PCL composite film. By varying the extent of swelling for the composite film, the length scale of the mineral gradient could be tuned from 5.8 to 55 μm.

#### 3.1.2 Diffusion of Thermal Energy

In addition to the diffusion of substances such as molecules and nanoparticles, the diffusion of thermal energy can also be leveraged for the fabrication of functionally graded materials. Thermal energy diffusion occurs when a temperature gradient exists between two materials in direct contact, moving from the hotter region to the colder one. The presence of a heat source and a sink quickly establishes a temperature gradient in the material involved. A graded gel can be fabricated when a temperature gradient is applied to a system containing a thermosensitive sol–gel precursor. Analogous to how Fick’s Law models mass diffusion, heat conduction (thermal energy diffusion) can be modeled using Fourier’s Law, predicting how heat distributes and temperature changes over space and time, and potentially guiding the design of functionally graded materials.

A notable example where energy diffusion is utilized to fabricate graded materials is the creation of a poly­(dimethylsiloxane) (PDMS) gel with a gradient in stiffness. As a biocompatible thermoset polymer, PDMS has been widely used in biomedical applications, including gene delivery,^101^ implant coating,^102^ and *ex vivo* and *in vivo* scaffolds.^103,104^ Voelcker and co-workers fabricated PDMS with a controlled gradient in cross-linking density and stiffness using the commercially available Sylgard 184 kit, aiming to modulate the osteogenesis of MSCs.^104^ The cross-linking relied on a platinum-catalyzed hydrosilylation reaction between the vinyl-terminated dimethylsiloxane base material and the Si–H bond-containing curing agent.^105,106^ Given the established influence of curing temperature on cross-linking density and mechanical strength,^107–110^ the authors utilized a hot plate held at 120 °C to generate a temperature gradient. The approach produced a continuous gradient in stiffness, ranging from 190 kPa to 3.1 MPa across a distance of 12 mm, which reduced the number of samples required for investigating the mechanotransduction of mammalian cells and enabled high-throughput *in vitro* experiments.

The fabrication method based on thermal diffusion is also applicable to polymers capable of physical cross-linking through noncovalent interactions, such as hydrogen bonds, ionic interactions, or hydrophobic interactions.^111^ Poly­(vinyl alcohol) (PVA), for instance, can be physically cross-linked without involving chemical cross-linkers through the formation of hydrogen bonds and chain entanglement. A notable method for achieving physical cross-linking is freeze–thawing, which is particularly effective for fabricating functionally graded materials. In one study, Lee and co-workers prepared PVA-based cylindrical hydrogels with a gradient in stiffness using a liquid nitrogen (LN_2_)-contacting gradual freeze–thawing method.^112,113^ During freezing, water in the PVA polymer solution crystallized and expelled the polymer chains from the ice lattice, increasing the local concentration of PVA in the nonfrozen region. The high concentration of polymer facilitated the formation of intermolecular hydrogen bonds, creating PVA crystallites that served as physical cross-linking points to stabilize the network. The final hydrogel structure directly depended on the freezing temperature and cooling rates. In the fabrication (Figure 15A), LN_2_ was placed at the bottom of a cylindrical mold containing a mixer of PVA and hyaluronic acid (HA). The pool of LN_2_ served as a freezing source to generate a temperature gradient along the vertical direction. The thermal energy diffusion resulted in a crystallinity gradient from the bottom to the top (Figure 15B), which in turn yielded a gradient in compress modulus (or stiffness) decreasing with the distance from the freezing source (Figure 15C).

**Figure 15:**
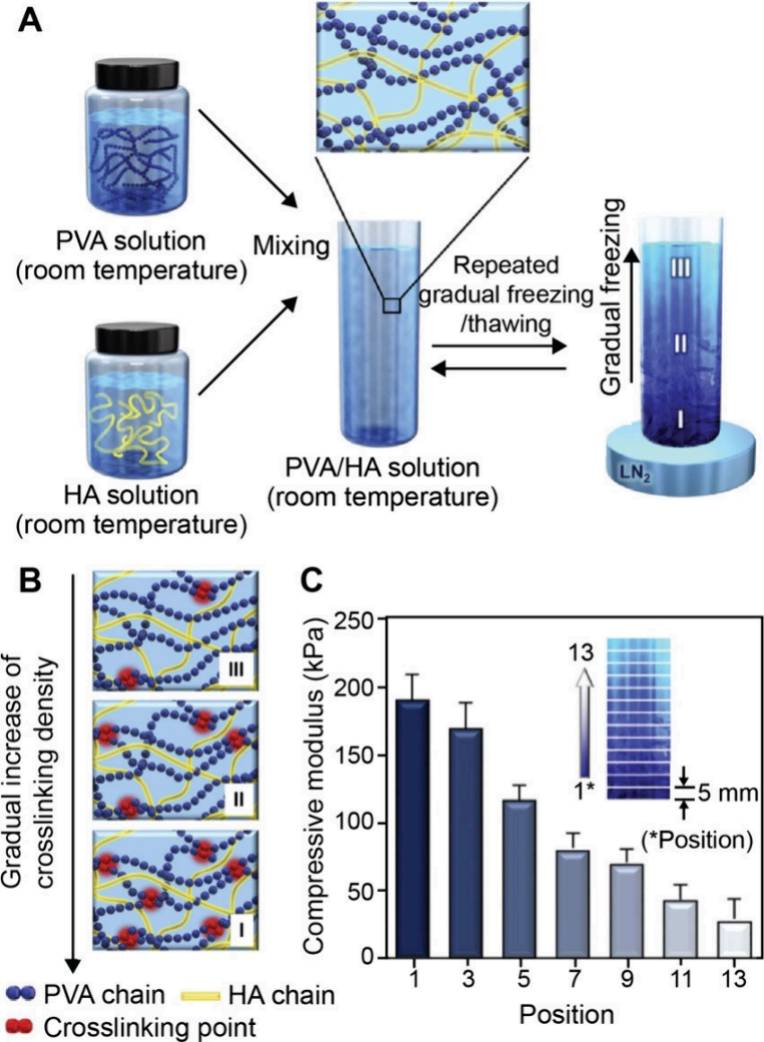
Fabrication of materials with a gradient in cross-linking density by leveraging the diffusion of thermal energy. (A) Schematic showing the gradual increase in cross-linking density of a PVA/HA mixture through repeated freeze–thawing cycles with LN_2_ at the bottom. (B) Mechanism of the formation of graded PVA/HA hydrogel with a gradual increase in cross-linking density from the top to the bottom. (C) Gradient in mechanical property (compressive modulus) along the longitudinal direction (*n* = 3) as a function of the distance from the LN_2_-contacted side. Reproduced with permission from ref 113. Copyright 2016 Elsevier.

Beyond freeze–thawing, PVA can also be cross-linked through heat treatment. Although this approach has not yet been experimentally demonstrated for creating graded materials, it may offer a theoretically feasible method for fabricating hydrogels with graded properties. Heating a PVA solution disrupts the hydrogen bonds between water and the hydroxyl groups of the polymer, thereby freeing the groups to interact with each other and form PVA crystallites that stabilize the 3D hydrogel network.^114,115^ The resulting PVA hydrogels exhibit mechanical properties dependent on the cross-linking density. They are also expected to show enhanced stability in an aqueous environment and improved selective permeability due to reduced water interactions.^116^ As a result, supplying a heat source to generate a temperature gradient within a PVA solution could produce PVA structures with graded selective permeability, opening avenues for biomedical applications. In principle, the method can be extended to other polymers exhibiting temperature-dependent curing or sintering properties. For instance, methylcellulose (MC) undergoes a process like that of PVA at elevated temperatures. High temperatures can reduce the interactions between MC chains and water, exposing −CH_3_ groups and driving strong hydrophobic interactions among these groups, thereby facilitating the self-assembly of an interconnected gel network.^117^ It is reasonable to hypothesize that the diffusion of thermal energy can be leveraged to produce graded MC hydrogels. More broadly, a deeper understanding of the mechanisms governing temperature-dependent sol–gel transitions would significantly advance efforts in fabricating functionally graded materials.^118^

### 3.2 Force-Driven Movement

In addition to diffusion, the movement of molecules and nanoparticles under an external force can also be used to create gradients in bulk materials. The technique utilizes an externally applied force (e.g., gravitational, centrifugal, magnetic, or electrostatic) to induce differential migration of molecules or nanoparticles within a matrix based on their intrinsic properties (e.g., size, shape, density, magnetic susceptibility, and charge density, among others). This approach leverages the external energy input to break diffusion constraints, offering unparalleled speed, precision, and design flexibility for creating continuous gradients. By tightly manipulating the magnitude, direction, and/or duration of the applied force(s), as well as the physicochemical properties of the matrix, nanoparticles are driven to segregate spatially in the matrix. The controlled fractionation process generates compositional gradients, resulting in regions exhibiting distinct physicochemical properties, such as density,^119^ refractive index,^120^ conductivity, and/or mechanical modulus.^33,121^ Particularly, a multitude of theoretical models have been developed through the years to simulate the gradient before the fabrication process, allowing for prediction and control of the extent and directionality of a gradient, offering a high degree of customization in the development of functionally graded materials. The fabrication strategy provides new opportunities for engineering complex material systems that mimic the multifunctionality and adaptability of natural tissues, thereby advancing the frontiers of biomaterials science.

#### 3.2.1 Gravitational Force and Buoyancy

Earth exerts a gravitational force on any object with mass. Such a force can induce a gradient in concentration by causing differential settling of objects based on the mass or diffusion within a matrix. In one example, Li and co-workers fabricated a conductive liquid metal-PVA hydrogel containing a gradient in liquid metal content by leveraging the sedimentation of liquid metal microdroplets of different sizes within the hydrogel.^122^ First, liquid metal microdroplets with diameters in the range 1–25 μm were obtained through sonication in ethanol. The liquid metal microdroplets were dispersed in a PVA solution and then poured into a dog-bone-shaped polytetrafluoroethylene mold. The relatively larger liquid metal microdroplets would settle down faster than smaller ones and assemble at the bottom of the PVA matrix due to gravity (Figure 16A). Subsequently, the resulting solution was subjected to freeze–thawing cycles to form a PVA hydrogel with a gradient in liquid metal content (Figure 16B). As shown in the optical and SEM images of the lower and upper surfaces of the liquid metal-PVA hydrogel (Figure 16C), the silvery, reflective appearance observed on the lower surface indicated gravitational accumulation of liquid metal microdroplets. The bottom-enrichment, driven by the self-gravity of the droplets, imparts the hydrogel with ultrahigh conductivity while preserving the integrity of the PVA matrix.

**Figure 16:**
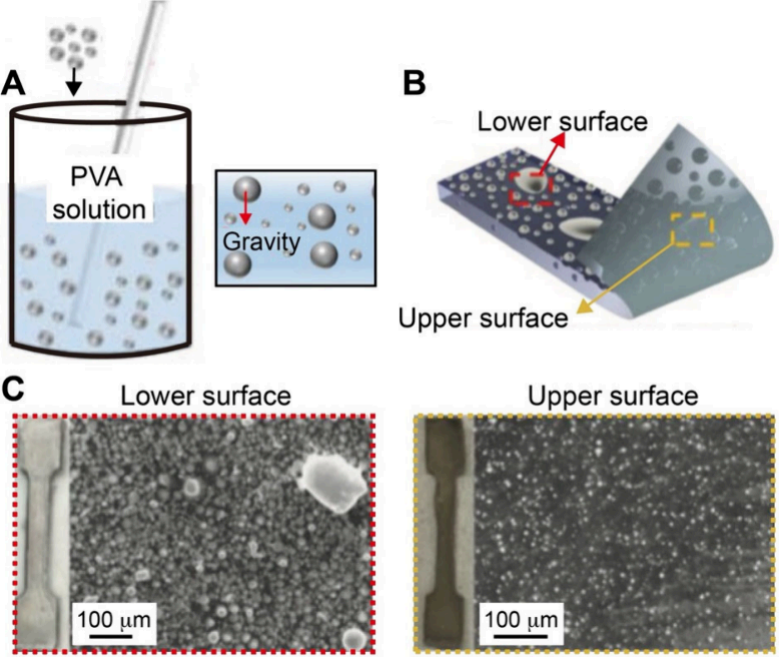
Fabrication of a graded material through size-dependent, gravitational-force-driven sedimentation. (A) Schematic showing the fabrication of liquid metal-PVA hydrogel by leveraging the gravity-induced sedimentation of liquid metal microdroplets. (B) Schematic showing the as-obtained liquid metal-PVA hydrogel. (C) Optical and SEM images of the lower and upper surfaces of the liquid metal-PVA hydrogel. Reproduced with permission from ref 122. Copyright 2023 the Author(s) (CC BY 4.0).

Tightly controlling the movement of nanoparticles under gravitational force, such as limiting their movement in a matrix, also enables the formation of graded materials. In one demonstration, a gradient was created by controlling the gravitational-force-driven movement of HAp nanoparticles in the pores of an inverse opal scaffold (Figure 17A).^1^ Inverse opal scaffolds are characterized by a well-defined, highly ordered array of uniform and interconnected pores. With uniform pores, interconnecting windows, and excellent batch-to-batch reproducibility in physical properties, inverse opal scaffolds serve as a versatile and biomimetic platform for tissue repair, including bone, cartilage, and osteochondral regeneration. In a typical process, uniform gelatin beads with an average diameter of ca. 200 μm were fabricated using a microfluidic device and then used as a template for generating an inverse opal scaffold. The gelatin beads were assembled into a cubic close-packed lattice in a centrifuge tube, followed by preheating at 80 °C to induce necking (partial fusion) between adjacent beads. After heating for 15 min, a methanol suspension of HAp nanoparticles was added to the centrifuge tube. Driven by gravitational force, the HAp nanoparticles settled onto the top surface of the lattice and entered the void spaces among the gelatin beads. Since the beads were soft and sticky at this temperature, the HAp nanoparticles would stick to the surface of the gelatin beads. As the annealing time increased, the pores at the top surface of the lattice gradually closed, thereby limiting the movement of nanoparticles and facilitating the formation of a mineral gradient. Afterward, the lattice was infiltrated with a PLGA solution to fix the HAp nanoparticles trapped in the void spaces. After removing the gelatin template, an inverse opal scaffold with a gradient in mineral content was obtained. Figure 17B shows a representative microcomputed tomography (micro-CT) image of the graded inverse opal scaffold, clearly indicating a gradual change in mineral content along the vertical direction through the scaffold. Mean pixel intensity (MPI) analysis of the micro-CT images in Figure 17C further confirmed the graded HAp content along the vertical direction of the scaffold.

**Figure 17:**
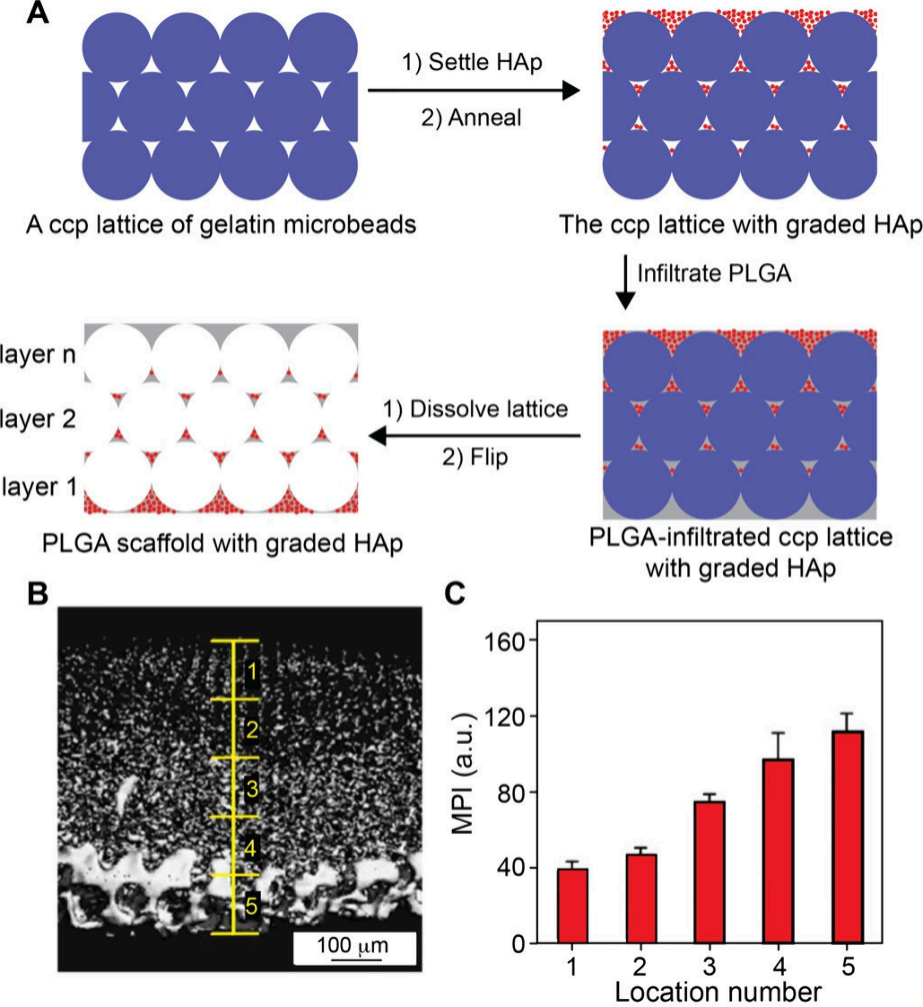
Fabrication of graded materials through gravity-driven limited sedimentation. (A) Schematic showing the preparation of a PLGA inverse opal scaffold with a mineral gradient. (B) Micro-CT image of the graded PLGA inverse opal scaffold and (C) MPI analysis of the graded scaffold. The yellow lines in (B) indicate the boundaries of the analyzed areas. Reproduced with permission from ref 1. Copyright 2017 Wiley-VCH.

Buoyancy, a net upward force exerted on a substance immersed in a fluid, arises from the gravity-induced pressure gradient within that fluid. Archimedes’ principle dictates the upward or downward movement of objects based on their density relative to that of the surrounding fluid. Greater density differences lead to faster sedimentation, while increased base material viscosity slows the movement. Therefore, the buoyancy-induced movement of nanoparticles can also be used to fabricate graded materials. By fine-tuning the density and viscosity of the fluid, the sedimentation of the substance and thus the resultant gradient can be tuned. For example, Shukla and co-workers fabricated a cenosphere/polyester resin composite exhibiting a graded distribution of Cenospheres by employing buoyancy-driven diffusion.^123^ Utilizing the difference in density between the two substances and the good gelation capability of the resin, the authors achieved a continuous gradient in Cenospheres over a distance of 250 mm. The material showed gradients in mechanical properties, including dynamic modulus, compressive strength, and fracture toughness, which are dependent on the concentration of Cenospheres. In another report, Zhang and co-workers prepared PVA/bacterial cellulose (PVA/BC) scaffolds with a gradient in HAp content using the buoyancy-driven method, with BC acting as a viscosity modifier for PVA to control the movement of HAp.^124^ The HAp-graded scaffolds showed good osteoinductivity when cultured with preosteoblast cells, holding promise for bone regeneration.

To generalize the buoyancy-driven approach, Stevens and co-workers developed a controlled two-component mixing system capable of producing graded distributions of various cargo species through a simple injection of one fluid material into another, followed by polymerization or gelation to preserve the gradient (Figure 18A).^119^ Both stepwise and continuous gradients can be created by varying the injection rate and/or the density differences between the two phases (Figure 18B). As suggested by the authors, the only requirement was two miscible and curable liquid phases with a sufficient difference in density. In cases where the density difference is too small to provide enough driving force, a density modifier can be included to facilitate the formation of a gradient. This method has broad applicability across a range of cargoes, encompassing inorganic (Au nanoparticles), organic (liposomes), and biological (dextran and avidin) nanomaterials and a variety of base materials such as gelatin methacryloyl, gellan gum, agarose, and acrylate polymers (Figure 18C). As expected, the tissue engineering constructs featuring a gradient in BMP-2 could induce graded osteogenesis.

**Figure 18:**
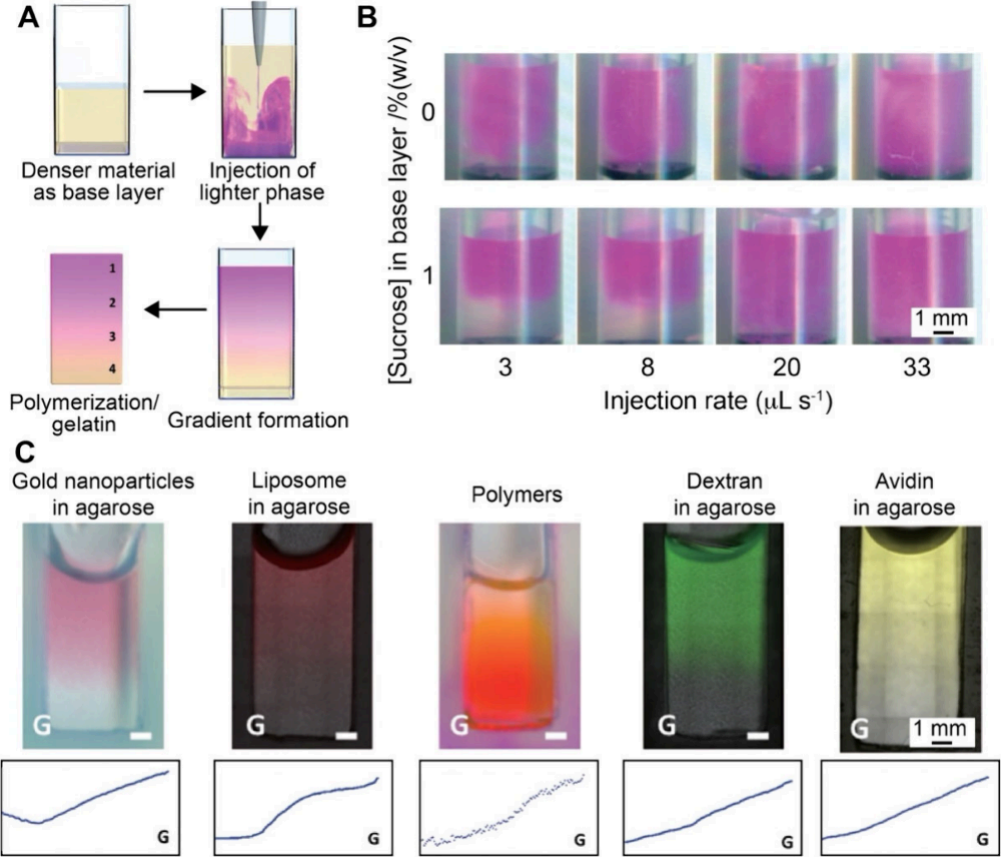
Buoyancy-driven formation of a graded material. (A) Schematic showing the formation of a graded material by buoyance-induced equilibration between two types of solutions or suspensions with different densities. (B) Photographs showing that the graded pattern can be controlled by adjusting the injection rate and the sucrose concentration in the base layer. (C) Photographs of the as-obtained graded materials and intensity profiles along the longitudinal direction for each structure. Reproduced with permission from ref 119. Copyright 2019 the Author(s) (CC BY 4.0).

#### 3.2.2 Centrifugal Force

While gravitational force and buoyancy provide a simple means for generating gradients, the resulting structures are inherently limited by the density and size of the particles, restricting the scope of applications. In contrast, utilizing more controllable forces, such as centrifugal, electrostatic, and magnetic forces, to control the movement of nanoparticles offers significant advantages for gradient formation. The advantages include enhanced control over the gradient profile, increased material versatility, accelerated processing, and the capability to generate gradients in materials with complex geometries. Moreover, the use of these forces helps minimize environmental disturbance while ensuring greater consistency and precision in gradient formation. As a good example, centrifugation-driven sedimentation has been actively explored to fabricate functionally graded materials. When applied to a homogeneous particle suspension in a viscous fluid, centrifugal force generates a graded distribution of the dispersed phase. Governed by well-established theoretical models, the process enables rapid and controlled fabrication of graded materials. Stoke’s law can be used to describe the movement of spherical particles:^125–128^

$$v=\frac{\left|\rho_{p}-\rho_{f}\right|\frac{F_{c}}{F_{g}}gD_{p}^{2}}{18\eta }$$

where *v*, ρ_*p*_, ρ_*f*_, *F*_*c*_, *F*_*g*_, *g*, *D*_*p*_, and η represent particle velocity, density of the particle, density of the fluid, centrifugal force, gravity force, gravitational acceleration, diameter of the particle, and viscosity of the fluid, respectively.^129^ In applying centrifugation-driven sedimentation to fabricate functionally graded materials, theoretical predictions of the movement of the dispersed phase should be correlated with key centrifugation parameters, including rotational speed, slope angle, distance from the center of the rotor, size of the centrifuge container (diffusion distance), and duration of centrifugation.^130^ For instance, as demonstrated by Cölfen and co-workers, high rotational speeds yield a sigmoidal gradient of particle distribution due to the dominance of sedimentation over diffusion. In contrast, low rotational speeds typically generate an exponential gradient.^131^

The centrifugation-driven sedimentation method can be used to generate gradients in pore size, porosity, and composition. For example, Lee and co-workers fabricated a PCL scaffold with gradients in pore size and porosity by centrifuging PCL fibrils, followed by heat treatment to weld the fibrils and thus preserve the gradients.^132^ In a follow-up study, leveraging the gradient in surface area associated with the porosity difference, a graded distribution of growth factor was achieved by immobilizing them on the PCL surface. The resultant scaffold could be used to guide cell migration, nerve repair, and angiogenesis.^133^ Guided by a theoretical model, Scaglione and co-workers successfully fabricated PCL- and collagen-based scaffolds with gradients in porosity and HAp content using a combination of centrifugation and freeze-drying to mimic the structure of bone.^130^ The fabricated scaffolds matched theoretical predictions, demonstrating their potential for customizable design.

#### 3.2.3 Electrostatic Force

An electric field can also drive the formation of a gradient when the precursor consists of charged species. In a typical process, a precursor solution or suspension is positioned between two electrodes of opposing charges. The applied electric field can induce electrophoretic migration, resulting in a graded distribution of charged species, which is then preserved by curing the matrix. The velocity of the moving charged species, also known as electrophoretic mobility (*v*_*ep*_), depends on the charge (*q*), size (*r*), viscosity of the medium (η), and intensity of the electric field (*E*):^134^

$$v_{ep}=\frac{q}{6\pi \eta r}E$$

The equation provides a valuable framework for estimating and predicting the resulting gradient, despite potential variations in viscosity during the sol–gel transition. Manipulating the electric field intensity, gelation parameters, and the charge and size of the charged species offers practical handles to control the gradient profile.^135^

The electrostatic force-based method has enabled the creation of gradients in composition, structure, and/or mechanical properties. To generate a compositional gradient, the active component needs to be intrinsically charged or chemically modified before being exposed to the electric field. Similarly, using charged polymers or precursors, such as silk fibroin,^121,135,136^ as the matrix enables the creation of structural or mechanical gradients. Multiple gradients can also be simultaneously incorporated into the bulk of a material.^137^ In one example, Han and co-workers prepared shape-morphing hydrogels with a gradient in silk fibroin network that could conformally interface with biological tissues having complex morphologies or large curvatures.^136^ Specifically, a mixture of silk fibroin, horseradish peroxidase (HRP), and hydrogen peroxide (H_2_O_2_) was placed between two electrodes. Catalyzed by HRP, silk fibroin macromolecules were cross-linked into a homogeneous hydrogel in the presence of H_2_O_2_. The application of an electric field across the electrodes resulted in the accumulation of H^+^ near the anode, attracting the negatively charged silk fibroins and generating a gradient in cross-linking density across the hydrogel (Figure 19A,B). The internal morphology was preserved via freeze-drying and examined using 3D X-ray microscopy (Figure 19C) and SEM (Figure 19D). Both methods consistently gave a denser network closer to the anode, and the gradient was extended throughout the hydrogel. The gradient in cross-linking density naturally translated to a gradient in mechanical property. In particular, the cross-linking density of the hydrogel could be tuned by varying the initial concentration of H_2_O_2_, rendering a wide range of modulus values to match the properties of diverse target tissues for biomedical applications. In another example, Liu and co-workers also developed a silk fibroin-based hydrogel with dual gradients in mechanical strength and TGF-β1.^121^ Under the electric field, negatively charged silk fibroins migrated toward the anode, increasing cross-linking density and thus mechanical strength near the anode. Concurrently, the growth factor, encapsulated in polymer nanocapsules with a tunable positive surface charge, was attracted to the cathode. The dual-graded system synergistically guided stem cell differentiation, facilitating the regeneration of cartilage and subchondral bone in a rabbit injury model.

**Figure 19:**
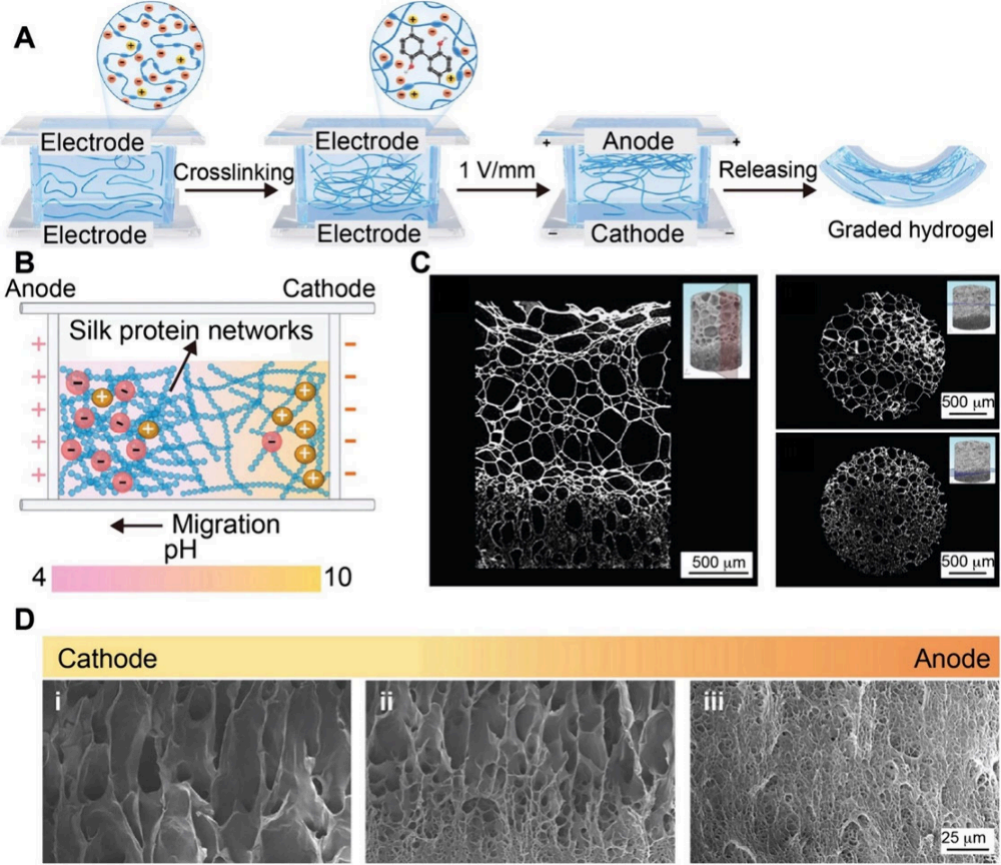
Fabrication of graded materials using electrostatic force or electric field. (A) Schematic illustration of the fabrication of a silk fibroin hydrogel with a structural gradient. The chemically cross-linked silk fibroin network migrates toward the anode under an electric field, resulting in a graded hydrogel after being released from the electrodes. (B) Schematic showing the migration of the silk fibroin network under an electric field, which also results in a pH gradient. (C) Cross-sectional X-ray scan images of the graded silk fibroin hydrogel, with the cutting plane shown in the corresponding inset. (D) Cross-sectional SEM images of the graded silk fibroin hydrogel, showing the decrease in pore size from the cathode (*i*) to the anode (*iii*). Reproduced with permission from ref 136. Copyright 2023 the Author(s) (CC BY-NC-ND 4.0).

In the case where the polymers that construct the matrix are electrically neutral, charged cross-linkers can be employed to facilitate the formation of a graded material under an electric field. In a series of reports, for example, Xu and co-workers demonstrated that Laponite, a negatively charged synthetic clay, could serve as a good physical cross-linker for poly­(*N*-isopropylacrylamide) (PNIPAM) and be leveraged to fabricate graded PNIPAM hydrogels.^137–140^ When subjected to an electric field, Laponite particles migrated toward the positively charged anode to generate a gradient in concentration that decreased from the anode to the cathode. As a result, the cross-linking density of the PNIPAM hydrogel decreased when approaching the cathode, rendering a material with a gradient in stiffness. Similarly, Chang and co-workers used negatively charged, naturally derived tunicate cellulose nanocrystals as both the cross-linker and reinforcement filler for a PNIPAM-based nanocomposite hydrogel to improve its mechanical properties.^141^ In another study, Yan and co-workers fabricated a gel with a gradient in Young’s modulus to mimic the human finger skin.^142^ A cationic cross-linker that accumulated around the cathode was used to generate the gradient. Following photo-cross-linking and solvent displacement, an ionic liquid-based gel was obtained (Figure 20A). In general, the intensity of the electric field needs to be optimized, as excessively high voltages can cause gel decomposition or hinder sol–gel transition due to the depletion of cross-linker near the anode. As shown by the cross-sectional SEM images in Figure 20B, the pore size increased from cathode to anode, correlating with a decrease in cross-linking density. The structural gradient led to a corresponding gradient in Young’s modulus (Figure 20C), which decreased by more than 4-fold from the cathode to the anode. Mimicking human skin, the graded gel showed enhanced sensitivity to low pressures and greater tolerance to high pressures compared to the homogeneous counterpart. Compressive stress–strain simulations showed that the material detectably displaced at 1 kPa and was still compressible at a pressure as high as 1 MPa (Figure 20D).

**Figure 20:**
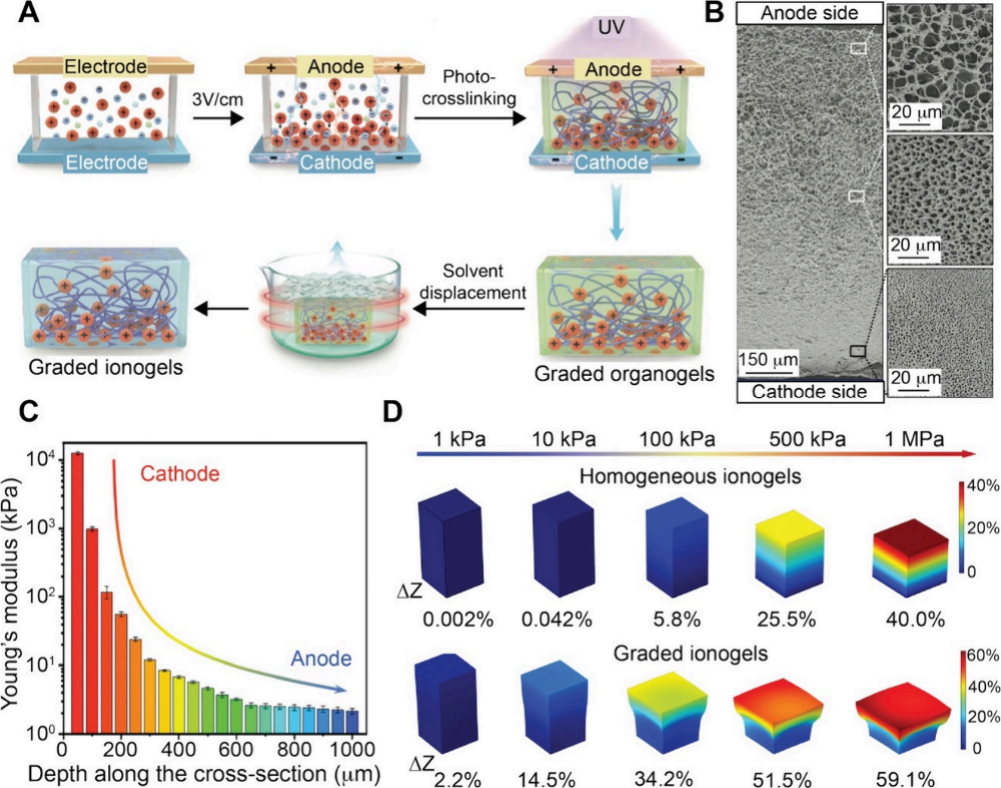
Fabrication of a graded ionic liquid-based gel under an electric field. (A) Schematic showing the fabrication process that employs an electric field to prepare a graded ionic liquid-based gel. (B) Cross-sectional SEM images of the entire structure (left side) and a partially enlarged view (right side) of the freeze-dried ionic liquid-based gel. (C) Gradient in Young’s modulus from the cathode to the anode. (D) Simulations comparing the pressure-response of homogeneous and graded ionic liquid-based gel. Reproduced with permission from ref 142. Copyright 2021 Wiley-VCH.

#### 3.2.4 Magnetic Force

As one of the four fundamental forces of nature, the magnetic force arises from the motion of charges in an electromagnetic field. Magnetic force is exerted when a magnetically responsive component is placed in a magnetic field. For example, when SPIONs are dispersed in a matrix and a magnetic field is applied, the magnetic force drives their migration within the fluid.^143^ The process is also referred to as magnetophoresis, which moves the nanoparticles along the magnetic field lines,^144,145^ generating a gradient in concentration of the magnetically responsive component. Magnetophoresis can be used to fabricate composites with graded properties. In one demonstration, a graded composite material was fabricated by leveraging the magnetophoresis capability of SPIONs in a resin matrix (Figure 21A).^146^ A partially cured resin composite containing Fe_3_O_4_@SiO_2_ nanoparticles was spin-coated onto a layer of partially cured resin film. Subsequently, a magnetic field was applied along the vertical direction using a permanent magnet to move the nanoparticles. After completely curing the resin by exposure to UV light, a composite with a graded distribution of Fe_3_O_4_@SiO_2_ nanoparticles was obtained. Depending on the size and fraction of the particles, the matrix viscosity, and the magnetic field distribution, the length scale of the gradient could be controlled in the range of 10 μm to a few millimeters (Figure 21B).^147^

**Figure 21:**
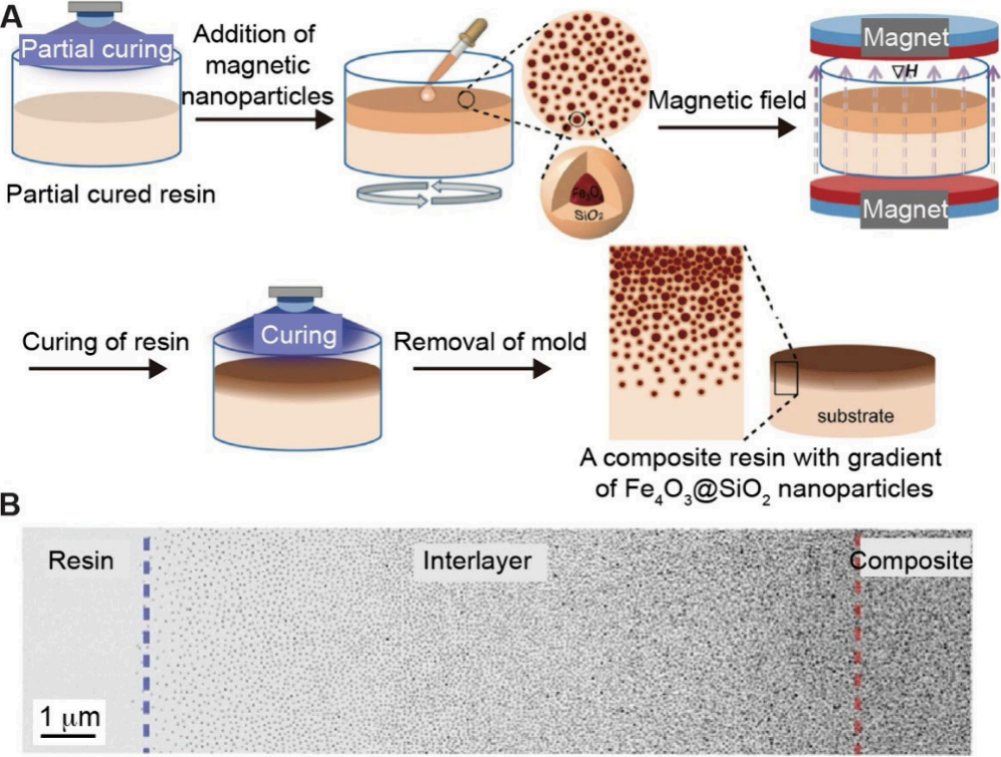
Fabrication of graded materials with compositional gradient in a magnetic field. (A) Schematic illustration of the steps for the creation of graded composite materials comprised of a resin and Fe_3_O_4_@SiO_2_ nanoparticles. (B) Transmission electron microscopy image of a slice of the graded composite material to show the spatial distributions of Fe_3_O_4_@SiO_2_ nanoparticles. (A) Reproduced with permission from ref 146. Copyright 2018 Wiley-VCH. (B) Reproduced with permission from ref 147. Copyright 2017 The Royal Society of Chemistry.

Magnetic force can also be used to control the microstructural architecture in graded materials. For example, during magnetically assisted slip casting, an external magnetic field was applied to dynamically align magnetically responsive particles, such as alumina platelets coated with SPIONs, to achieve graded composites with locally varying texture.^148^ Specifically, as shown in Figure 22A, a slurry of the platelets was poured into a porous, disc-shaped gypsum mold. Capillary forces pulled the liquid into the pores of the mold, leaving behind densely packed magnetic platelets, during which an external rotating magnetic field would determine the orientation of the platelets layer-by-layer as they were solidified (Figure 22B). By rotating or reorienting the magnetic field in a time-programmed manner, the alignment of the anisotropic particles could be tuned to create graded composites with locally varying textures. The magnetic field’s directional control enables the formation of periodic reinforcement patterns (e.g., alternating plywood-like layers) or site-specific gradients (e.g., dentin-enamel junctions in synthetic teeth) by fixing the orientation of magnetic particles through sintering or infusing with polymers/metals (Figure 22C).

**Figure 22:**
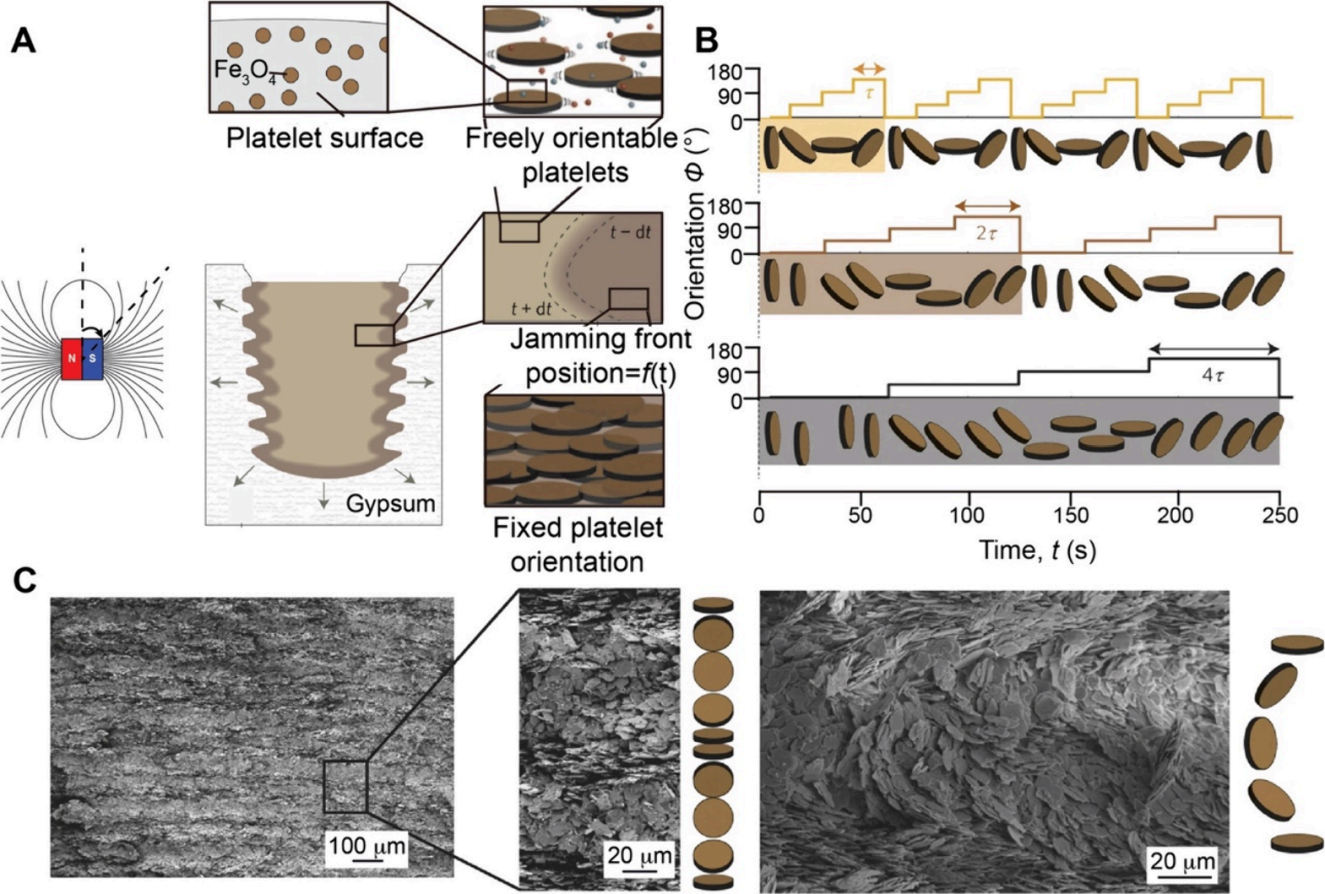
Fabrication of a material with a graded texture using a magnetic field. (A) Schematic illustration of the fabrication process. A porous, disc-shaped gypsum mold is filled with a suspension of alumina platelets coated with SPIONs, whose orientation could be tuned using an external magnetic field. Since the dimensions of the pores of the mold are smaller than the platelets, wetting the pores generates capillary forces that continuously remove the liquid phase from the suspension to build a layer of jammed particles next to the mold wall while fixing their orientations. (B) Gradient in platelet orientation can be tuned by programming the orientation of the magnetic field. (C) SEM images showing the periodic platelet orientation patterns. Reproduced with permission from ref 148. Copyright 2015 Springer Nature.

#### 3.2.5 Shear Force

While the aforementioned methods create graded distributions primarily by exploiting the differences in density or other properties of the components, an alternative strategy manipulates material flow to obtain a gradient in fiber alignment using the differences in shear force. As demonstrated by Nazhat and co-workers, a negative pressure arising from a change in the diameter of the flow channels could be used to align collagen fibrils.^149^ Following a similar principle, Abhyankar and co-workers fabricated a 3D collagen matrix with a gradient in fiber alignment to mimic the ECM of a tumor. A pattern of positive, zero, and negative extensional flow could be achieved by expanding and constricting the microfluidic channel (Figure 23A).^150^ The shear force exerted by positive extensional flow aligned the collagen fibrils, while the absence of that in the negative flow region resulted in random fiber orientation. A continuous gradient in fiber alignment was achieved across the interface, as shown by the reflectance confocal microscopy images in Figure 23B. This technique enabled the fabrication of complex fiber architectures, providing new routes for *in vitro* replication of the ECM.

**Figure 23:**
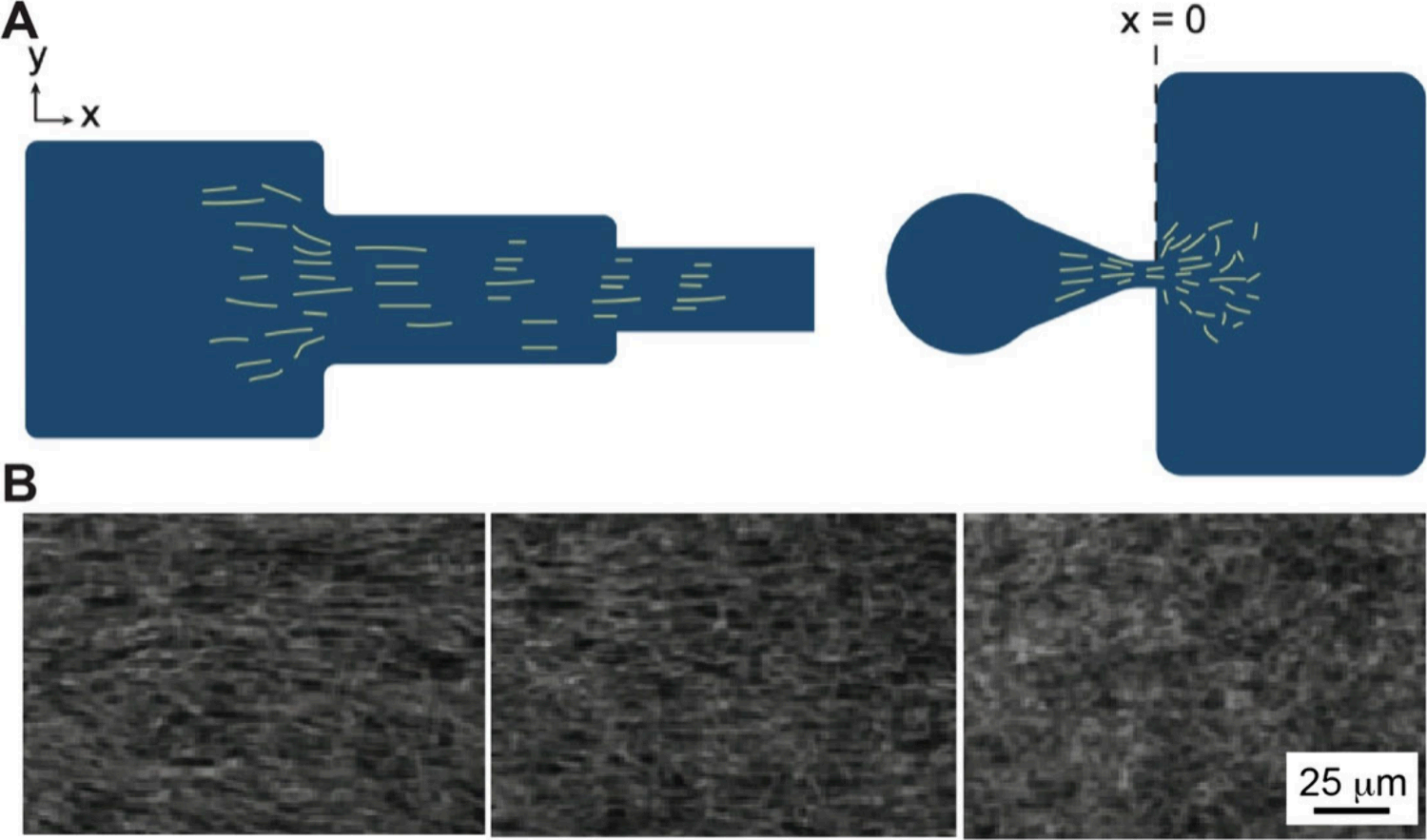
Fabrication of 3D collagen gel with continuous, graded fiber alignment by adjusting the input flow rate, which results in different shear forces. (A) Schematic showing the structural gradient obtained by controlling the flow pattern and thus changing the shear force. Constricting the flow leads to alignment, while expanding the flow results in random orientation. (B) Reflectance confocal microscopy images showing the gradient in alignment after the formation of the collagen gel. Reproduced with permission from ref 150. Copyright 2023 the Author(s) (CC BY-NC 4.0).

### 3.3 Layer-by-Layer Fabrication

Layer-by-layer fabrication is a straightforward and versatile method for generating bulk gradients through iterative, sequential deposition or adsorption of various components, such as polymers, nanoparticles, or biomolecules, onto a substrate or within a mold. The gradient is achieved by deliberately modulating the concentration, composition, and/or other parameters during each cycle of deposition. The method enables a tight control over composition, thickness, and functionality across the depth of a material in each layer. Electrospinning or 3D printing-based layer-by-layer fabrication also allows for the creation of gradients with complex architectures. Although layer-by-layer fabrication often leads to the formation of stepwise gradients, it is still possible to create continuous gradients by involving other techniques, such as microfluidic devices.

#### 3.3.1 Layer-by-Layer Casting

A simple method for fabricating graded materials involves layer-by-layer casting of solutions or suspensions with varying concentrations on a substrate (Figure 24A). A subsequent sintering or molding process can integrate the different layers into a graded material comprised of two, three, or multiple phases. In one study, Lu and co-workers fabricated a triphasic scaffold through layer-by-layer casting for musculoskeletal interface tissue engineering (Figure 24B).^151^ Briefly, Phase A, corresponding to the soft tissue, was formed by sintering the segments of a polyglactin polymer mesh in a cylindrical mold at 150 °C for 20 h. Phase B, corresponding to the fibrocartilage region, was constructed by sintering the PLGA microspheres at 55 °C for 5 h. Phase C, corresponding to the bone, was fabricated by sintering a mixture of PLGA and bioactive glass microspheres. Subsequently, Phases A and B were joined and sintered on top of Phase C by heating, creating a graded triphasic material. Due to the layer-by-layer approach, a stepwise gradient rather than a continuous one was formed. After implantation at the anterior cruciate ligament-to-bone interface, the phase-specific matrix heterogeneity was formed in the triphasic scaffold (Figure 24C,D).

**Figure 24:**
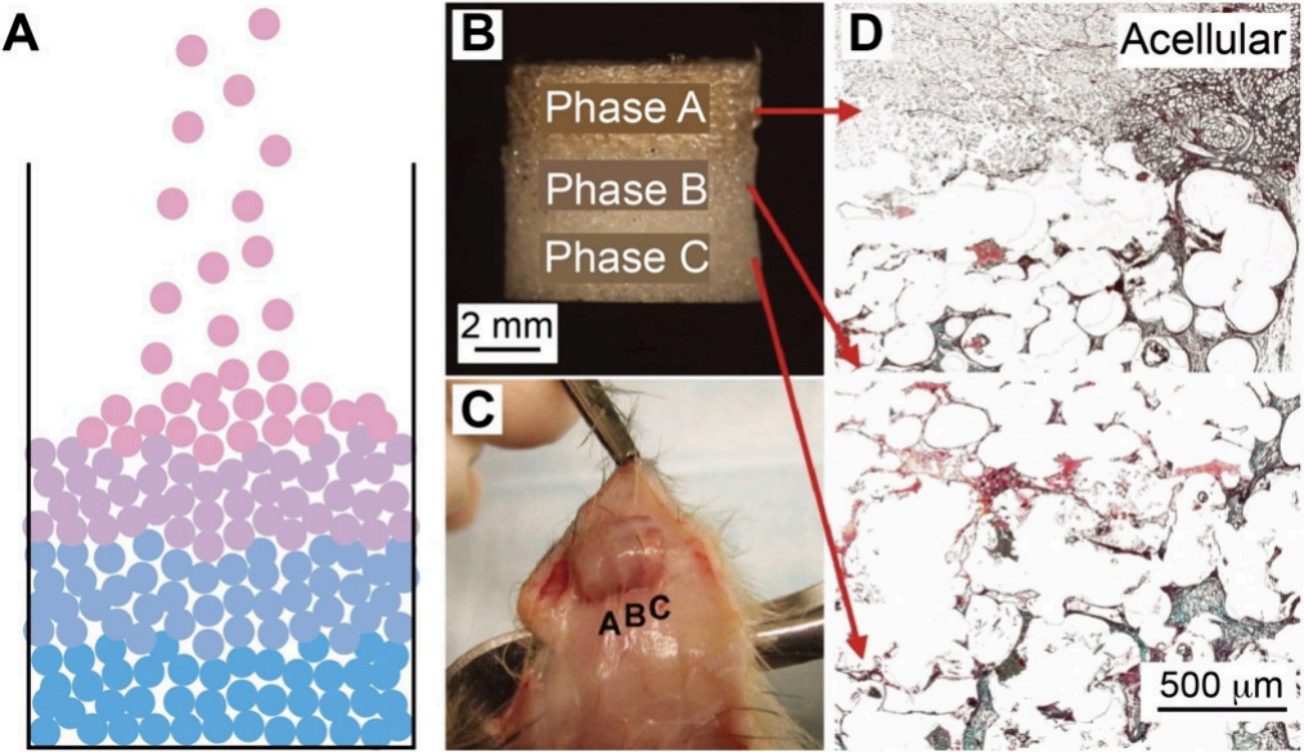
Fabrication of graded scaffold using layer-by-layer casting. (A) Schematic illustration of the layer-by-layer casting method. (B) Triphasic scaffold with three distinct phases (A, B, and C) designed to match soft tissue, interface, and bone, respectively. (C) Photograph showing the triphasic scaffold retrieved at 4 weeks postimplantation. (D) Optical micrographs of the explanted scaffold after Modified Goldner’s trichrome staining. Reproduced with permission from ref 151. Copyright 2008 Wiley-VCH.

#### 3.3.2 Brush-Coating and Spin-Coating

For layer-by-layer casting, the number of layers to be deposited and the thickness of each layer are limited due to the difficulty of operation and repeatability. Other deposition techniques, such as brushing or spin-coating, allow for a tight control over the number and thickness of each layer in the graded material. For example, brush-coating was employed to fabricate HAp-graded scaffolds with the length scale of the gradation controlled down to the micrometer scale (Figure 25A).^152^ Specifically, a series of 16 wt % PLGA solutions in 1,4-dioxane with varying HAp concentration were brush-coated on the surface of a substrate using a nail polish brush in a layer-by-layer manner. As the HAp/PLGA suspension of the subsequent layer was deposited, the freshly introduced solvent would anneal the adjacent two layers and contribute to the formation of a seamless interface. After complete removal of the solvent through evaporation, a scaffold with a gradient in HAp content was obtained. As shown by energy-dispersive X-ray spectroscopy (EDX) mapping and the elemental ratio in Figure 25B-D, the scaffold had a graded transition zone of ca. 37 μm in thickness. This value is comparable to the length in mineral gradation at the tendon-to-bone insertion. As shown in Figure 25E, the Young’s modulus monotonically increased with the increase in HAp content along the thickness direction. Such a mineral-graded scaffold offers great potential for mitigating interfacial stress concentrations between mechanically mismatched tissues and recapitulates a key structural characteristic of the native tendon-to-bone insertion.

**Figure 25:**
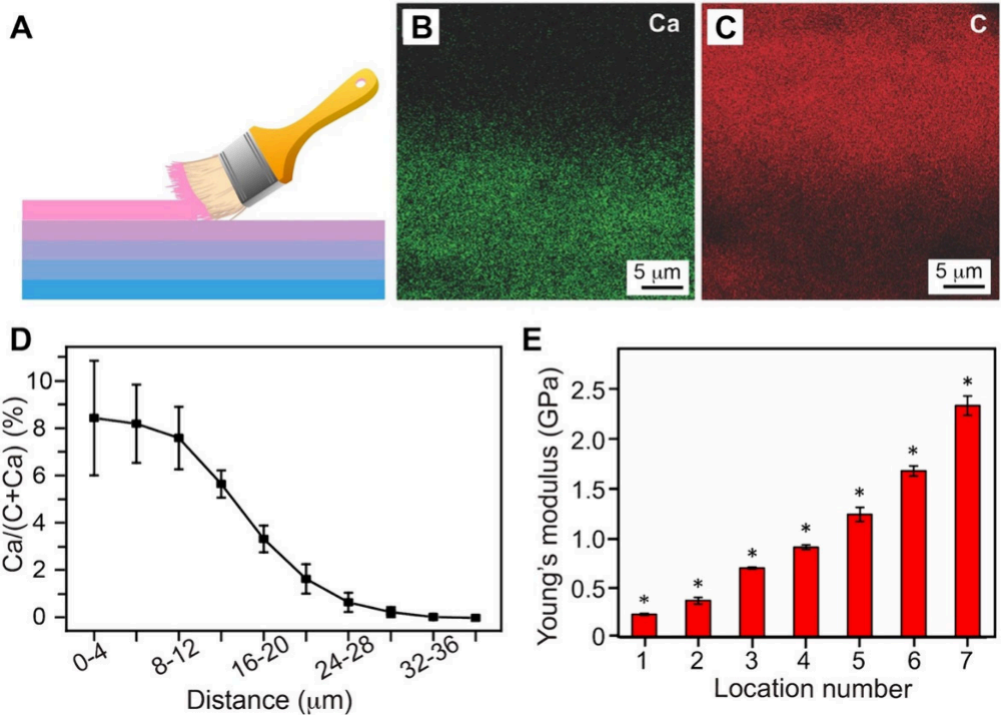
PLGA scaffold with a gradient in mineral content, which was fabricated through layer-by-layer brushing. (A) Schematic of the brush-coating method. (B, C) EDX mapping of elements in the transition zone showing the graded distributions of (B) calcium and (C) carbon, respectively. (D) Quantification of calcium content along the film thickness direction (*n* = 3), where the *x*-axis indicates the distance away from the position with the highest mineral content. (E) Local Young’s modulus along the mineral gradient (*n* = 6). The numbers 1 and 7 represent the unmineralized and highly mineralized regions, respectively. Significant differences for all pairwise comparisons are accepted at *p < 0.05. Reproduced with permission from ref 152. Copyright 2018 Wiley-VCH.

Compared to brush-coating, spin-coating offers a tighter control over the number and thickness of the deposited layers. Spin-coating refers to the process of creating a uniform coating with a well-controlled thickness by spinning a solution (or suspension) at a high speed. Xia and co-workers demonstrated that spin-coating could be adapted to fabricate scaffolds with mineral gradients that mimic those of the native tendon-to-bone insertion.^33^ Specifically, HAp/PCL suspensions with decreasing HAp concentrations were sequentially spin-coated on a silicon wafer (Figure 26A). During spin-coating, most of the solvent evaporated, resulting in a uniform PCL thin film with evenly distributed HAp nanoparticles. Raman spectroscopy data showed a monotonic change in HAp content through the depth of the graded HAp/PCL scaffold (Figure 26B,C). As a significant advantage over prior methods, the high reproducibility of spin-coating ensured both layer-to-layer and batch-to-batch consistency. In addition, the steepness of the gradient and the length scale of the mineral-graded zone could be conveniently tuned by adjusting the number of layers with specific HAp/PCL concentrations, enabling a customizable scaffold design. The method can be readily extended to other combinations of polymers and inorganic nanoparticles.

**Figure 26:**
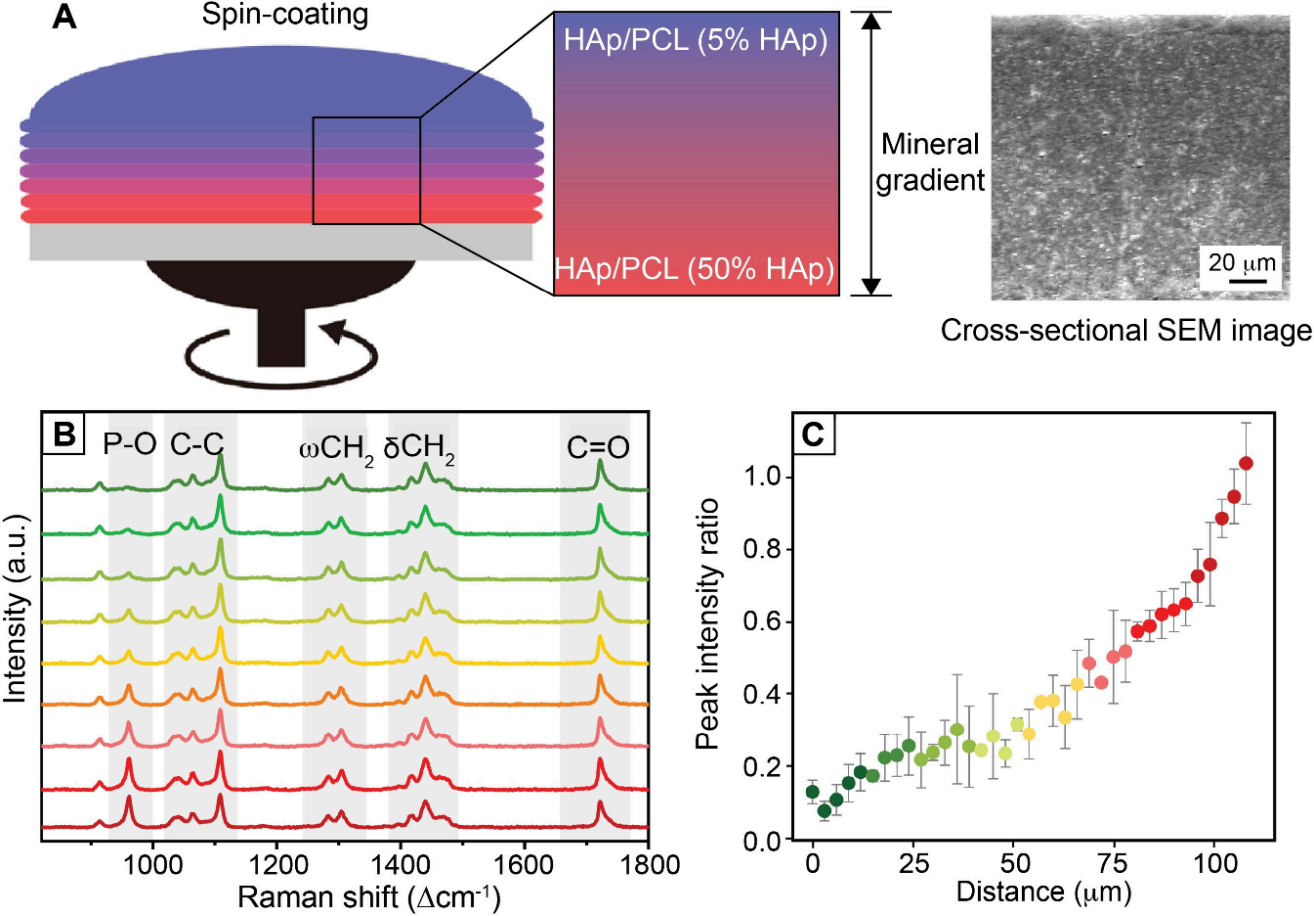
PCL scaffold with a gradient in mineral content fabricated using layer-by-layer spin-coating. (A) Schematic illustration of the fabrication process and a cross-sectional SEM image of the resultant graded HAp/PCL scaffold. (B) Representative Raman spectra recorded from different regions of the cross-section along the vertical direction of the graded scaffold. (C) Plot of the ratio between the intensities of the peaks at 960 cm^–1^ (P–O stretch) and 1724 cm^–1^ (C=O stretch), which correspond to HAp and PCL, respectively (*n* = 3). The data set in panel (C) was presented as mean ± standard deviation. Reproduced with permission from ref 33. Copyright 2024 Wiley-VCH.

#### 3.3.3 Electrospinning

Electrospinning can also be used as a powerful tool for constructing graded materials in a layer-by-layer fashion. With tunable and controlled composition, diameter, alignment, and porosity, electrospun fibers are widely used in biomedical applications as they closely mimic the structure of the ECM.^153^ Another advantage of electrospinning is that functional components or structures can be easily incorporated into the fibers using methods like blending, emulsion, and coaxial spinning.^154–156^ Deposition of fibers with different compositions and/or structures in a sequential manner enables the creation of graded materials with variations in mechanical, structural, and biological properties.

A tight control of material composition in each layer allows for the fabrication of scaffolds with distinct functional zones. A representative example is the trilayered periodontal scaffold developed by Janowski and co-workers.^157^ Using sequential electrospinning, they fabricated a scaffold consisting of three compositional layers. The bone-facing layer contained PLA and gelatin fibers incorporated with HAp nanoparticles to promote osteogenesis. The middle layer was composed of poly­(D,l-lactide-*co*-ε-caprolactone) to serve as a mechanically robust barrier with slow degradation. The epithelial-facing layer, also based on PLA/gelatin fibers, was loaded with metronidazole to confer antimicrobial activity. By altering the electrospinning solution for each layer and stacking them in a controlled sequence, they created a spatial gradient in functionality across the scaffold. The gradient design supported bone regeneration at the defect interface, prevented infection, and maintained structural stability, highlighting the potential of engineering graded materials for complex tissue repair.

In addition to composition, fiber morphologies, such as diameter, porosity, and alignment, can also be tuned during electrospinning for the layer-by-layer fabrication of graded materials. For example, Koh and co-workers fabricated a trilayer electrospun gelatin scaffold with increasing fiber diameters and pore sizes along its thickness using layer-by-layer electrospinning, in which the solution concentration, flow rate, and electrospinning time were systematically adjusted.^158^ The resulting mechanical gradient with improved fracture resistance and porosity enabled both load-bearing support and cell infiltration during tissue regeneration. Besides manipulating fiber composition and morphology during electrospinning, postprocessing techniques such as gas-foaming expansion have been leveraged by Xie and co-workers to introduce structural gradients into multilayered nanofiber mats. For instance, as shown in Figures 27A–C, sequentially electrospun four layers of PCL nanofibers with increasing concentrations of Pluronic F-127 enabled gradation in porosity after a foaming process with H_2_ gas.^159^ Because higher Pluronic F-127 concentrations would enhance H_2_ bubble retention during foaming, greater expansion and larger pore formation were achieved. As a result, a vertical gradient in porosity was created throughout the scaffold. The gradient can be used to induce spatial variations in oxygen availability, which may influence hypoxia-mediated stem cell responses and tissue-specific regeneration.

**Figure 27:**
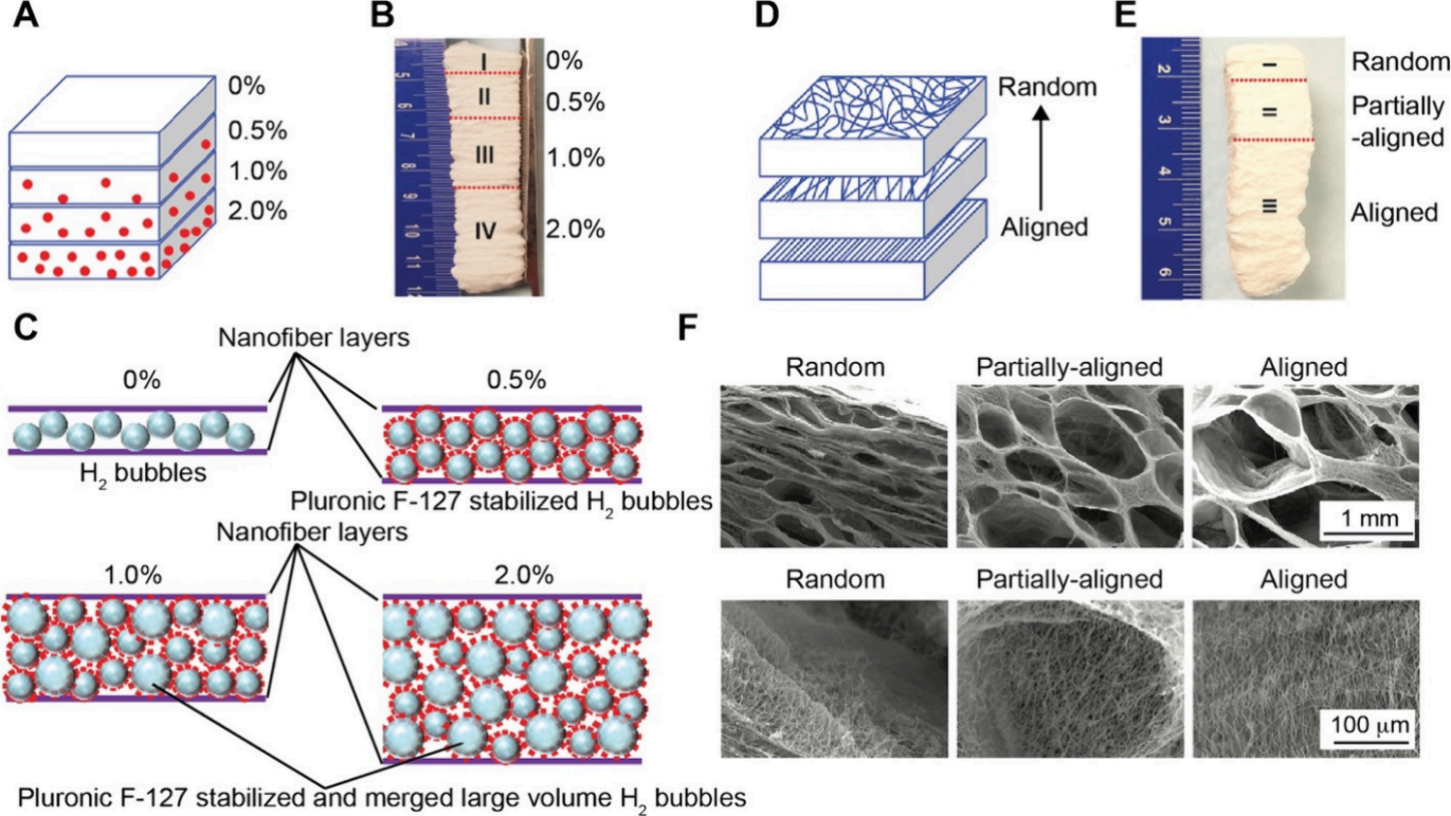
Fabrication of a graded material by layer-by-layer electrospinning. (A) Schematic showing a PCL nanofiber scaffold with increasing concentrations of Pluronic F-127 from top to bottom. (B) Photograph of the expanded PCL nanofiber scaffold after gas foaming, corresponding to the schematic in (A). (C) Schematic showing the formation of different porosity in a PCL nanofiber scaffold with varying concentrations of Pluronic F-127 during gas foaming. (D) Schematic showing the fabrication of a PCL nanofiber scaffold with dual gradients in porosity and fiber organization. (E) Photograph of the expanded PCL nanofiber scaffold with a gradient in fiber organization ranging from aligned (bottom) to partially aligned (middle) and random (top). (F) Cross-sectional SEM images of different layers of the expanded nanofiber scaffold, corresponding to the sample shown in (D, E). Bottom row: higher-magnification views showing fiber arrangement in each layer. Reproduced with permission from ref 159. Copyright 2020 Wiley-VCH.

Furthermore, the gradients in fiber alignment can be simultaneously incorporated by adjusting the rotational speed of the collector during the sequential electrospinning process.^159^ As illustrated in Figure 27D, the fiber alignment gradually changed from randomly oriented to highly aligned along the vertical axis. Subsequent gas foaming expansion of the alignment-graded mats resulted in dual gradients in porosity and fiber orientation (Figure 27E,F). Notably, the aligned regions exhibited more significant expansion, producing larger pores and reduced fiber density compared to the randomly oriented regions. The differential expansion behavior was attributed to the entanglement of random fibers, which resisted expansion more than aligned structures. The dual-graded architecture, spanning both porosity and alignment, offers a powerful strategy for replicating the multiscale anisotropy found in native tissues and for guiding cell infiltration, alignment, and differentiation. In addition to the variation in fiber alignment between layers, preserving alignment while varying orientation angles could also introduce different properties for the graded scaffolds. For example, Zou and co-workers constructed a multilayer fibrous composite by depositing aligned poly­(D,l-lactide)/HAp fibers through layer-by-layer electrospinning of aligned nanofibers at varying angles (0°, 30°, 45°, and 90°), resulting in a composite with graded mechanical properties.^160^ The mechanical gradients, along with the incorporation of HAp in the fibrous composites, provided favorable conditions for cell proliferation and osteogenic differentiation.

#### 3.3.4 3D Printing

3D printing, also known as additive manufacturing, has emerged as a versatile, rapid prototyping technique capable of fabricating objects with high structural and compositional complexities in a layer-by-layer manner. As an additive technique, 3D printing is ideal for layer-by-layer fabrication of graded materials as it enables real-time and tight manipulation of the local material composition and structural features (Figure 28A). Additionally, 3D printing also allows the creation of complex geometries. Among the seven broad categories of 3D printing techniques defined by the American Society for Testing and Materials (ASTM),^161^ material extrusion, material jetting, vat photopolymerization, and powder bed fusion are most commonly used to fabricate graded materials.^162,163^

**Figure 28:**
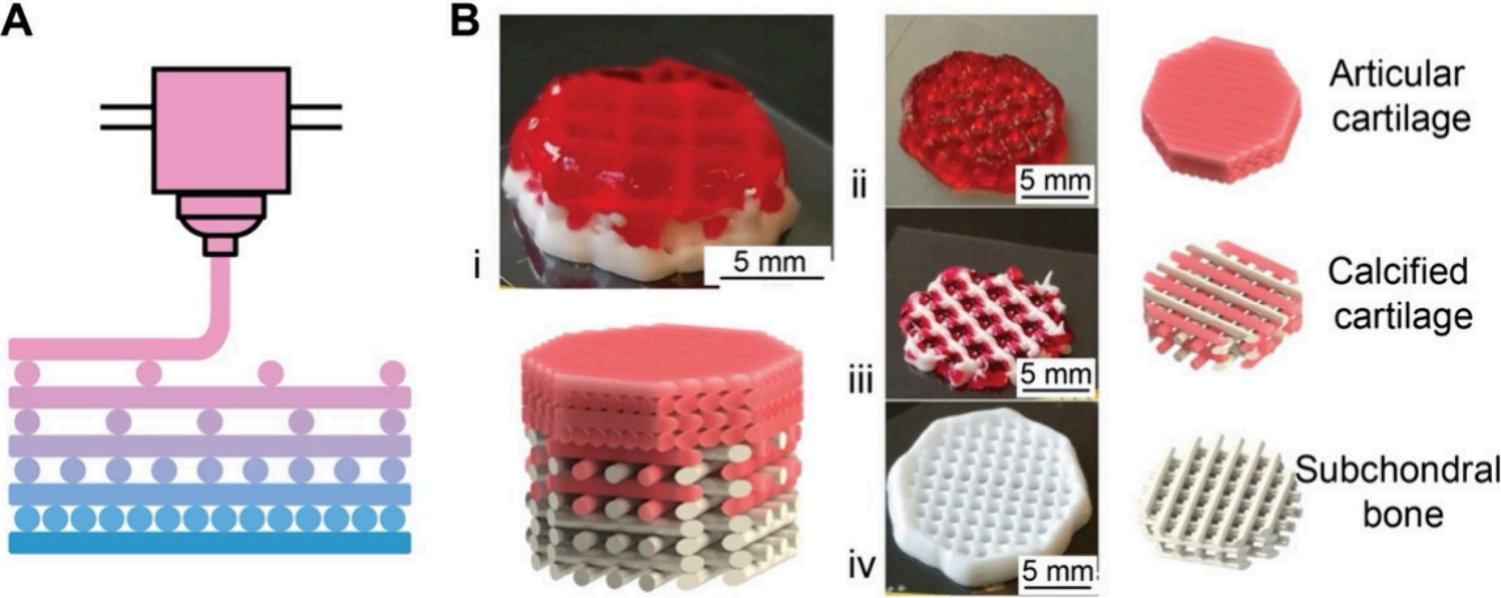
Fabrication of a graded scaffold using layer-by-layer 3D printing. (A) Schematic illustration of the layer-by-layer 3D printing process, demonstrating tight controls over both composition and structure. (B) A full-thickness multiphasic osteochondral scaffold fabricated using layer-by-layer 3D printing. (*i*) The combined structure, consisting of (*ii*) a cell-laden algMC zone resembling the articular cartilage; (*iii*) an interwoven network of cell-laden algMC and CPC resembling the calcified cartilage; and (*iv*) a CPC-based zone resembling the subchondral bone. Phenol red was added to the hydrogel for easy visualization. Reproduced with permission from ref 164. Copyright 2020 the Author(s) (CC BY 4.0).

In one study, Lode and co-workers fabricated a graded scaffold using material extrusion-based 3D printing to mimic the zonal structure of an osteochondral tissue, see Figure 28B­(*i*).^164^ The scaffold consisted of a stepwise gradient. One end of the scaffold comprised cross-linked alginate-methylcellulose (algMC) loaded with primary chondrocytes to mimic the articular cartilage, see Figure 28B­(*ii*). In the transition zone, an interwoven layer containing cell-laden algMC and mineralized calcium phosphate cement (CPC) was fabricated by alternating strand deposition to recreate the calcified cartilage structure, see Figure 28B­(*iii*). The other side consisted of mineralized CPC to mimic subchondral bone, see Figure 28B­(*iv*). The graded scaffold offered the possibility to embed chondrocytes for differentiation in both mineralized and nonmineralized environments. Additionally, the additive nature of 3D printing allows one to control the thickness, geometry, and microstructure of each layer. With the aid of magnetic resonance imaging data, patient-specific scaffolds can be created to match the anatomical features of an individual patient. As for the bone zone, the porosity of the inner and outer regions can be adjusted to match the highly porous cancellous bone and the dense cortical bone, respectively, as demonstrated by Eryildiz in his work on a PLA-based graded bone scaffold.^165^

Material jetting-based 3D printing also allows for the fabrication of compositionally graded materials by controlling the jetting of different materials from the multinozzle print heads. After deposition, the jetted material typically needs to solidify to form the 3D structure, usually via thermal, chemical, or photocuring mechanisms.^166^ Consequently, materials such as silicone elastomers^167^ and hydrogels^168,169^ are commonly used in material jetting processes for fabricating graded materials. Vat photopolymerization-based 3D printing can be used to fabricate graded materials by selectively curing liquid photopolymers layer-by-layer using a tightly controlled laser beam. Upon completion, the entire structure is usually subjected to postcuring by light or heat to improve the mechanical properties.^170^ Compositional gradient can be achieved using multivat systems that allow for layer-specific photopolymer changes^171^ or by integrating a mixing system to alter the resin composition before curing.^172^ Analogous to the use of a grayscale mask for creating surface gradients on photosensitive materials (Section 2.2), structural gradients can be formed within a single photopolymer by applying a continuously graded grayscale light pattern to modulate the curing conditions. For example, using an acrylate-based photopolymer, Qi and co-workers developed a single-vat gray scale digital light processing (DLP) method to fabricate complex structures with graded properties.^173^ Both the cross-linking density and the elastic modulus decreased as the percentage of gray scale increased, with the modulus being varied from 1.4 MPa to 1.3 GPa. The authors demonstrated that a presurgical limb model with stiff bone surrounded by pliable muscle could be fabricated in a single process using the technique. Compared to other 3D printing techniques, vat photopolymerization allows for high printing resolution (<2 μm) and therefore the creation of structures with intricate geometries.

Powder bed fusion represents another category of 3D printing extensively utilized for the fabrication of functionally graded materials. During the process, the powders are spread on a platform that gradually lowers as the layers build up, forming a powder bed. A high-power laser, such as Nd:YAG or CO_2_,^174^ provides thermal energy to fuse certain regions within the powder bed selectively. Graded materials can be fabricated by modifying either the powder composition or the energy input spatially. Achieving a compositional gradient using powder bed fusion often involves sophisticated procedures, such as premixing of powders^175^ or the use of specialized multipowder deposition systems to vary the material constituents across layers or regions.^176^ Ensuring homogeneity and tight control can be challenging. Structural gradients are more readily achievable because process parameters such as laser power, scanning speed, and scan strategy can be spatially modulated to customize local density or porosity.^177–179^

Despite the advantages offered by 3D printing in fabricating graded materials, several factors still require careful consideration for successful biomedical applications. Similar to other layer-by-layer fabrication techniques, 3D printing suffers from delamination between discrete layers. Many refinements to current techniques aim to resolve the problem while maintaining overall printing capability.^180,181^ However, the delamination issue would become more prominent when a graded structure with internal dissimilarities is fabricated. Another major challenge for broadening the biomedical applications of 3D printed structures is balancing printability and biocompatibility. In many 3D printing processes, only a limited number of biocompatible materials meet the rheological or thermal property requirements necessary for successful printing. Some methods, such as the fused deposition modeling, which involves a high-temperature melting step, are inherently incompatible with cell-loaded raw materials. Uncovering materials and processing conditions that maximize both printability and biocompatibility is of critical importance.

#### 3.3.5 Microfluidics Integrated with Layer-by-Layer Fabrication

As discussed in Section 2.4, microfluidic technology allows for the creation of gradients via controlled laminar flow and diffusion. Beyond surface patterning, microfluidics integrated with layer-by-layer fabrication can also produce functionally graded materials. Unlike surface gradients confined to a thin superficial layer, bulk gradients necessitate compositional control throughout the volume of the material. Microfluidic devices offer a distinct advantage over traditional mixing methods by providing stable laminar flows and enhanced control over solute distribution through embedded mixing modules.

In the generation of gradients, efficient mixing of multiple precursor solutions within the microchannels is critical. Advanced strategies such as chaotic advection, serpentine flow paths, and barrier-based mixers promote rapid and reproducible mixing, enabling the formation of a gradient across the bulk of a matrix.^182–184^ By tuning flow parameters and microchannel design, one can generate volumetric gradients that are essential for biomedical applications such as the fabrication of tissue scaffolds, drug delivery systems, and biomimetic constructs. The following examples highlight how microfluidics can be combined with layer-by-layer assembly to facilitate the construction of spatially controlled and compositionally graded materials. One representative example of advanced microfluidic mixing is the valve-based flow-focusing (vFF) chip developed by Abate and co-workers.^185^ In the design, the orifice size, where bubble or droplet breakup occurs, must be controlled. Fabricated using PDMS, the junction was sandwiched between a pair of pressurized dead-end channels acting as valves. The deformable PDMS walls and the orifice allowed for the gradual squeezing of the orifice based on the applied pressure, enabling dynamic control over bubble size without altering the flow rates of the immiscible phases. Such a tight and dynamic control enables the fabrication of both porous materials and functionally graded porous structures with spatially varying pore sizes, simply by adjusting the bubble size.

By combining a vFF junction microfluidic device with extrusion-based 3D printing, Guzowski and co-workers developed a porous scaffold featuring a graded internal architecture.^186^ The schematic of the vFF junction is shown in Figure 29A, where the intersection periodically injects air into a biopolymer solution to generate a liquid foam with a uniform bubble size. Real-time adjustment of the orifice geometry, as controlled by the applied gas pressure (Pv), allows one to tune the bubble size. Increasing Pv progressively altered the orifice cross-section from rectangular to triangular, ultimately constricting the lumen to block gas and aqueous flow (Figure 29B). Figure 29C shows optical micrographs of the bubbles formed under different Pv, demonstrating tunable bubble diameters ranging from 80–800 μm and good stability of the foam. By integrating the vFF device with an extrusion 3D printer and adjusting the pressure in a preprogrammable manner, they successfully deposited foam filaments in a predefined 3D shape while continuously varying the foam microstructure. Micro-CT images (Figure 29D) of the resulting scaffold revealed a spatially varying pore network, transitioning from smaller and compact pores at one end to larger, more open pores at the other. The microfluidic control allows for the creation of a gradient in pore size, demonstrating the versatility of laminar flow modulation for structural control. The capability starkly contrasts conventional foaming or phase-separation methods, which struggle to achieve well-defined spatial variations in pore size within one continuous material.

**Figure 29:**
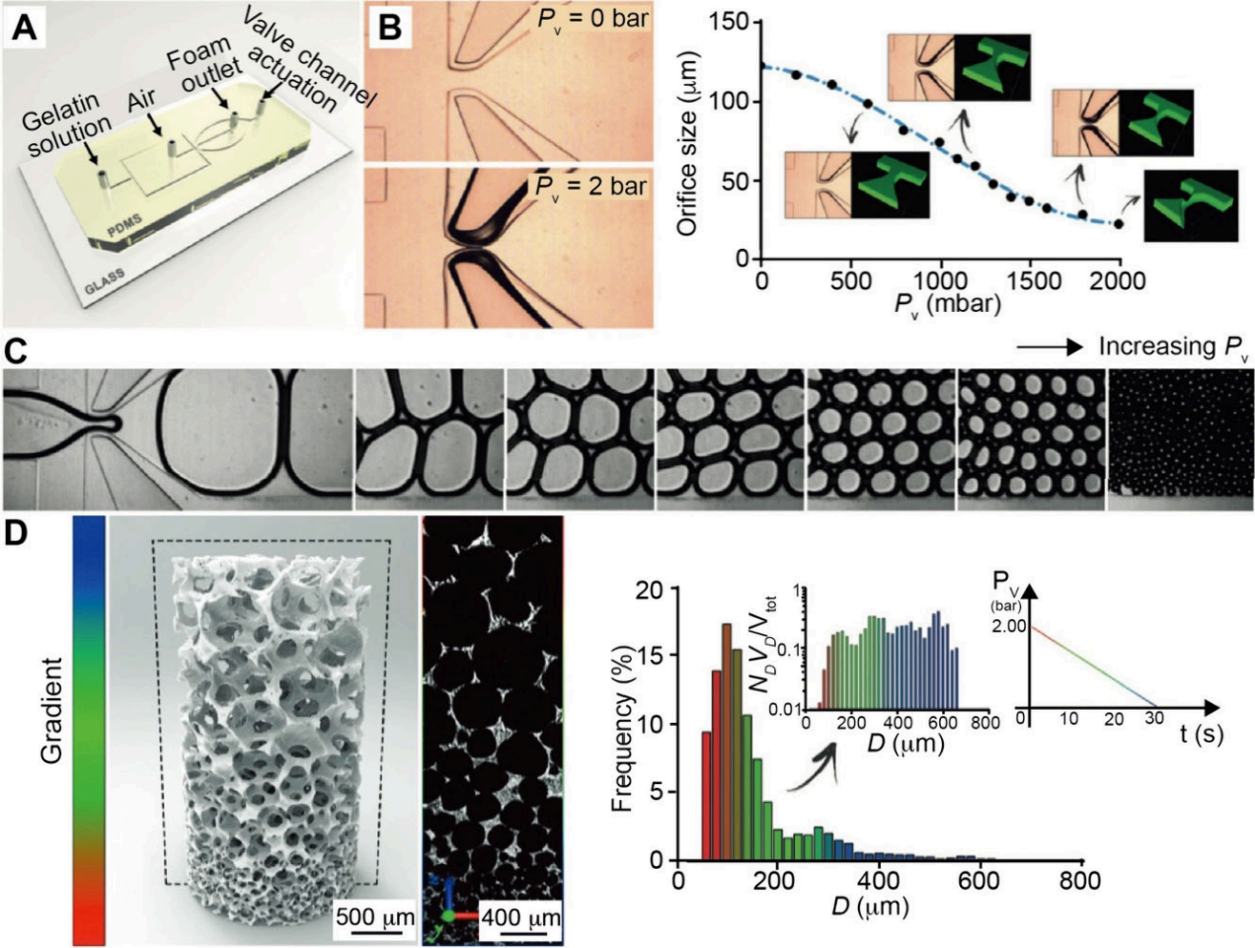
Graded materials fabricated using a microfluidic device. (A) Schematic showing the setup of the valve-based flow focusing chip. (B) Optical micrographs of the device for Pv = 0 and 2 bar showing the squeezing of the orifice, and orifice size as a function of Pv; the confocal Z stack renderings were obtained by injecting a fluorescent dye into the orifice. (C) Optical micrographs of the device during foaming for different Pv values. (D) 3D reconstructions of graded materials obtained from micro-CT scans, along with vertical cross-sectional images illustrating the variation in pore size along the *z*-axis (left). Corresponding normalized pore size distributions are shown on the right. Insets: The changes of Pv with the function of time. Reproduced with permission from ref 186. Copyright 2019 Wiley-VCH.

In another example, Kasarełło and co-workers employed microfluidic-assisted bioprinting to fabricate hydrogel constructs with gradients desired for osteochondral regeneration.^187^ They developed a microfluidic printhead that combined multiple hydrogel streams under laminar flow conditions to produce continuously graded bioinks (Figure 30A). Two different bioinks, each loaded with blue and red fluorescent microbeads, respectively, were introduced through separate inlets and converged at a Y-junction. The mixture then passed through a passive serpentine-based mixer that enhanced diffusion and enabled controlled-ratio blending before extrusion. By adjusting the relative flow rates of the two bioinks, the printer could deposit a filament that either exhibited abrupt transitions between materials to form a stepwise or gradual transitions in composition to yield a gradient. The resulting graded scaffold is shown in Figure 30B. This approach also enabled the fabrication of cell-laden hydrogel constructs with zonal compositional gradients that mimic the osteochondral interface. The constructs recapitulated the zonal differentiation from hyaline to calcified cartilage, exhibiting excellent cell viability and distinct cellular phenotypes. Notably, the use of microfluidics made laminar mixing gentle enough to preserve cell viability, allowing spatial variation in matrix composition without compromising printing fidelity.

**Figure 30:**
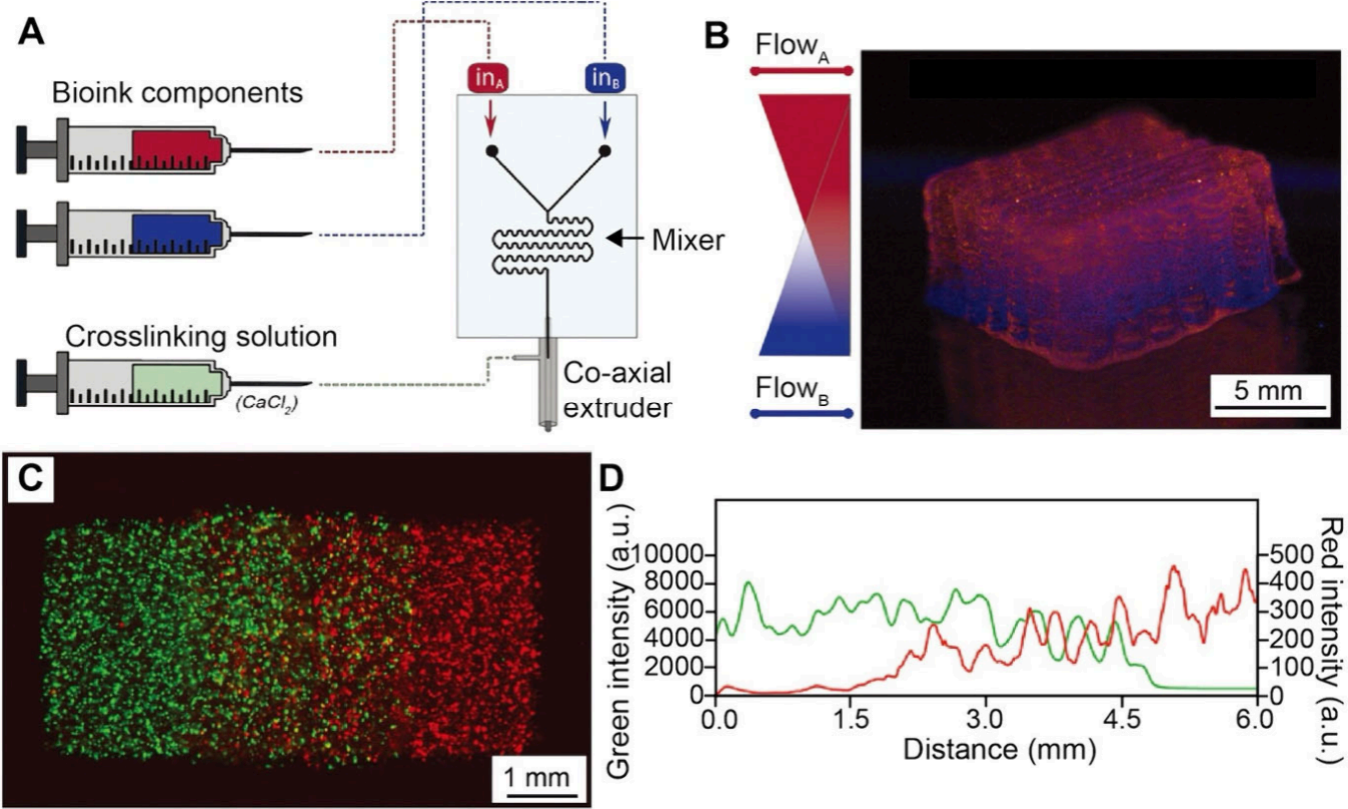
Graded materials fabricated using a combination of microfluidic devices and 3D printing techniques. (A) Schematic showing the fabrication of the graded scaffold. (B) Photograph of the graded scaffold. (C) Fluorescence micrograph showing the construct containing a gradient in RFP-HUVECs (red) and GFP-HUVECs (green) distribution. (D) Quantitative analyses of the fluorescence intensities in (C). (A, B) Reproduced with permission from ref 187. Copyright 2019 IOP Publishing. (C, D) Reproduced with permission from ref 172. Copyright 2021 Wiley-VCH.

An advanced microfluidic strategy for fabricating graded materials involves the integration of mixers with DLP bioprinting. DLP bioprinting employs a digital micromirror device to polymerize photocurable resins layer-by-layer in selected regions. In one study, Zhang and co-workers introduced a DLP bioprinting platform that utilized a microfluidic chaotic mixer to continuously vary resin composition in the printer’s vat, enabling a compositional gradient in the printed object.^172^ In the setup, two or more precursor solutions containing photopolymerizable bioinks with different compositions or cell suspensions were fed from separate inlets into a microfluidic chip where they underwent chaotic laminar mixing. The resulting mixture, at a predefined composition, then flowed into the DLP projection region, where a patterned light cured each layer. By adjusting the flow rates of the inlet streams layer-by-layer, the system can program a new composition for each printed layer. Using this method, the authors demonstrated the fabrication of various gradients in a single print, including a gradient in stiffness (via polymer concentration), a biochemical gradient (via dopant or growth factor concentration), and a gradient in cell density (via modulation of cell content in the bioink). Figure 30C highlights an example in which two different cell populations were distributed in opposite gradients across a printed tissue strand. In this case, one bioink contains green fluorescence protein (GFP)-labeled human umbilical vein endothelial cells (HUVECs), and the other contains red fluorescence protein (RFP)-labeled HUVECs. By inversely adjusting the flow rates during printing, the resulting hydrogel exhibited a dual gradient: one cell type gradually decreased from left to right, while the other increased. The dual gradients were confirmed by quantitative analyses of the fluorescence intensities in Figure 30D. Beyond cell placement, the same system was also used to fabricate scaffolds with a dual gradient in porosity and growth factor concentration, more closely mimicking the multivariate gradients found *in vivo*.

In summary, microfluidic platforms employ advanced mixing mechanisms and controlled fluid dynamics for the fabrication of graded materials with precise and reproducible volumetric gradients. In addition to enabling seamless transitions in chemical composition, mechanical properties, porosity, and cell density, microfluidic platforms integrated with extrusion, foaming, and vat-photopolymerization printers offer a scalable route to patient-specific or application-specific constructs. Taken together, the convergence of tight control over gradient and scalable fabrication strategies via microfluidics offers a robust route to advanced biomedical constructs for regenerative and personalized therapies.

Building on the above discussion, Table 2 offers a concise comparison of fabrication methods for functionally graded materials.

**Table 2:** Overview of Fabrication Methods for Functionally Graded Materials^a^

Technique	Control Strategy	Achievable Gradient	Gradient Control	Setup Complexity	Res./Cost/Rep./Scal.	Major Biomedical Applications	Ref.
Diffusion	Diffusion of molecules/nanoparticles	Composition, mechanical properties, porosity	**Good**: Diffusion time; temperature; matrix properties.	**Good**: Utilizes natural diffusion, often at ambient processing conditions.	3/5/1/5	Guiding cell migration and differentiation.	^97–100^
	Diffusion of thermal energy	Cross-linking density, mechanical properties	**Moderate**: A temperature gradient across the material; exposure time.	**Good**: Applicable to any material with temperature-dependent curing or sol–gel transitions, using simple heating/cooling setups.	3/5/1/4	High-throughput screening of cell mechanotransduction.	^104^,^112^,^113^
Force-driven movement	Gravitational force and Buoyancy	Composition, particle density	**Moderate**: Relative density difference between components; viscosity of the base material/matrix.	**Good**: Relies on fundamental forces, but limited by material choices where density differences are sufficient.	1/5/2/5	Tissue engineering scaffolds; conductive hydrogels.	^1^,^119^,^122–124^
	Centrifugal force	Composition, structure (porosity, pore size)	**Good**: Precise control via rotational speed; duration; rotor geometry.	**Moderate**: Requires access to a centrifuge.	2/4/3/2	Guiding cell migration; nerve repair; promoting angiogenesis; tissue engineering scaffolds.	^130^,^132^
	Electrostatic force	Composition, structure, mechanical properties	**Good**: Electric field intensity; the charge and size of the mobile species; matrix viscosity.	**Moderate**: Requires a controllable power supply and electrode setup.	4/3/3/2	Tissue engineering scaffolds; interfacial tissue repair.	^121^,^136–142^
	Magnetic force	Composition, microstructure (texture, particle alignment)	**Good**: Magnetic field distribution, intensity, and direction; matrix viscosity.	**Moderate**: Requires a magnetic field, which can be generated by simple permanent magnets.	4/4/3/3	Bioinspired wear-resistant composites; periodontal regeneration.	^146–148^
	Shear force	Structure (fiber alignment)	**Low**: Fluid flow rate	**Moderate**: Typically requires a microfluidic device to control flow patterns and generate shear forces.	3/3/2/2	In vitro replication of the tumor extracellular matrix.	^149^,^150^
Layer-by-layer fabrication	Layer-by-layer casting	Composition (stepwise)	**Low**: Composition of each discrete layer during casting.	**Good**: Simple method that can be done with basic lab equipment.	2/5/2/5	Interfacial tissue repair.	^151^
	Brush-coating and spin-coating	Composition	**Moderate**: Composition of each deposited layer and the total number of layers.	**Moderate**: Spin-coating requires specialized equipment, while brush-coating is more accessible.	4/4/4/3	Interfacial tissue repair.	^33^,^152^
	Electrospinning	Composition, structure (fiber diameter, porosity, alignment), mechanical properties	**Good**: Electrospinning solution and collection parameters.	**Moderate**: Requires a specialized electrospinning setup.	5/3/5/3	Periodontal regeneration; guiding cell migration, alignment, and differentiation.	^157–160^
	3D printing	Composition, structure (porosity), cross-linking density, mechanical properties	**Good**: Composition, thickness, geometry, and microstructure of each layer.	**Low**: Requires access to specialized 3D printing equipment, which can be expensive.	5/1/5/2	Interfacial tissue repair; patient-specific scaffolds; presurgical models.	^164^,^167–174^,^177–179^
	Microfluidics integration	Composition, structure (porosity), cell density, mechanical properties	**Good**: Composition for each printed layer by adjusting inlet flow rates in real-time.	**Low**: Requires complex integration of microfluidic devices with other fabrication systems like 3D printers.	5/1/5/1	Interfacial tissue repair; tissue engineering scaffolds.	^172^,^185–187^

a Res. (Resolution): 5 = Excellent (e.g., < 10 μm); 1 = Poor (e.g., > 1 mm). Cost: 5 = Very Low; 1 = Very High. Rep. (Reproducibility): 5 = Excellent; 1 = Poor. Scal. (Scalability): 5 = Excellent; 1 = Poor.

## 4 Biomedical Applications

The availability of functionally graded surfaces and materials presents new opportunities for biological studies and medical applications. By mimicking the seamless transitions in composition, structure, and properties found in native tissues, the graded surfaces and materials allow for a tight control over cell-material interactions and corresponding biological responses. In this section, we explore the broad use of graded surfaces and materials across a spectrum of applications, ranging from investigation of fundamental biological processes, including cell migration and organoid development, to addressing clinical challenges, such as repair or regeneration of interfacial, neural, and cardiovascular tissues, in addition to wound dressing and development of emerging platforms for high-throughput drug screening and biomedical actuation.

### 4.1 Investigation of Cell Migration

Cell migration is fundamental to numerous physiological and pathological processes, including tissue regeneration, wound healing, immune response, and cancer metastasis. In native tissues, cell migration is often directed by spatial gradients in biochemical signals, mechanical properties, and structural cues. In mimicking the guidance cues, functionally graded surfaces and materials have been developed to manipulate cell migration. Both 1D and 2D gradients have been fabricated to replicate the biological microenvironments *in vitro*. The graded systems have enabled modulation of cell behavior, offering viable strategies for regenerative medicine, tissue engineering, and *in vitro* disease modeling.

Cell migration is a well-coordinated process that begins with cell polarization, where the cell establishes a front leading edge and a rear trailing edge.^188^ At the leading edge, external cues, such as biological effectors or mechanical stiffness, activate signaling pathways that promote actin polymerization, pushing the membrane forward to form protruding structures like lamellipodia or filopodia.^189,190^ Simultaneously, focal adhesions form at the front to anchor the cell to the substrate, allowing it to pull its body forward.^191^ At the rear trailing edge, contractile forces generated by actomyosin activity release adhesions and thus retract the cell body.^190,191^ The cycle of protrusion, adhesion, traction, and retraction enables directional movement of cells, particularly when guided by biochemical and/or physical cues presented in gradients.

In the presence of gradients, cells would take directional migration by converting spatial cues into intracellular signaling cascades. For instance, cell migration in response to substrate stiffness, known as durotaxis, is a well-established phenomenon whereby cells preferentially migrate from softer to stiffer regions.^192^ The behavior arises from differential traction forces at the cell–substrate interface, where cells sense mechanical cues through focal adhesions and respond by reorganizing their cytoskeleton to achieve directional migration.^193^ The process begins with cell polarization, where the leading edge of the cell becomes enriched with actin protrusions and adhesion complexes, whereas the trailing edge contracts and detaches. On a stiffness-graded surface, directional migration arises from the preferential stabilization of cellular protrusions on the stiffer region, which establishes the net traction force required for moving up the gradient.^194^ In one study, Wang and co-workers constructed a microstructured substrate with a gradient in stiffness using a composition-tunable hydrogel system.^195^ They demonstrated that mouse myoblast cells migrated toward stiffer regions with increased speed and persistence. In another study, He and co-workers engineered a hydrogel with a gradient in stiffness to promote angiogenesis and osteogenesis.^196^ By integrating a thermosensitive, amino acid-based hydrogel with photo-cross-linkable methacrylated alginate, they used stepwise light exposure to generate a gradient in modulus *in situ* spanning from ca. 0.6 to 500 kPa. The spatially controlled gradient in mechanical property effectively directed the migration and differentiation of MSCs, suggesting the utility of a gradient in stiffness in regenerative medicine.

Cells are sensitive not only to the static stiffness of their microenvironment but also to dynamic mechanical cues, such as deformation of the substrate and a gradient in strain. A gradient in strain can emerge in biological tissues during processes like wound healing, morphogenesis, or muscle contraction, where spatial variations in deformation occur. In such an environment, cells tend to migrate toward the region with either lower or higher strain, depending on their phenotype and mechanosensitive signaling network, a phenomenon referred to as “tensotaxis”.^197,198^ In one study, Sun and co-workers reported a programmable cell-stretching device capable of generating a graded strain to investigate directional migration of cells.^199^ By applying mechanical stretching to a PDMS substrate with carefully designed cut-outs, the authors created a graded strain while maintaining uniform stiffness and ligand distribution, isolating the gradient in strain as the exclusive mechanical cue. As shown in the strain maps, the triangular design created a graded field of strain (Figure 31A) while the square design yielded a uniform distribution of strain (Figure 31B). When rat embryonic fibroblasts were seeded on the substrates, their migration trajectories revealed a clear bias toward the lower strain region under static graded conditions (Figure 31C), a behavior absent under uniform strain. As illustrated in Figure 31D, further analysis of individual cells showed polarized membrane protrusions and focal adhesion formation on the lower strain side, and retraction on the higher strain side, confirming that the graded strain alone can serve as a potent cue to guide directional cell migration. Additionally, the tensotaxis behavior was supported by computational modeling with an extended motor-clutch framework, highlighting the fundamental role of the graded strain in guiding cell migration during tissue morphogenesis and repair.

**Figure 31:**
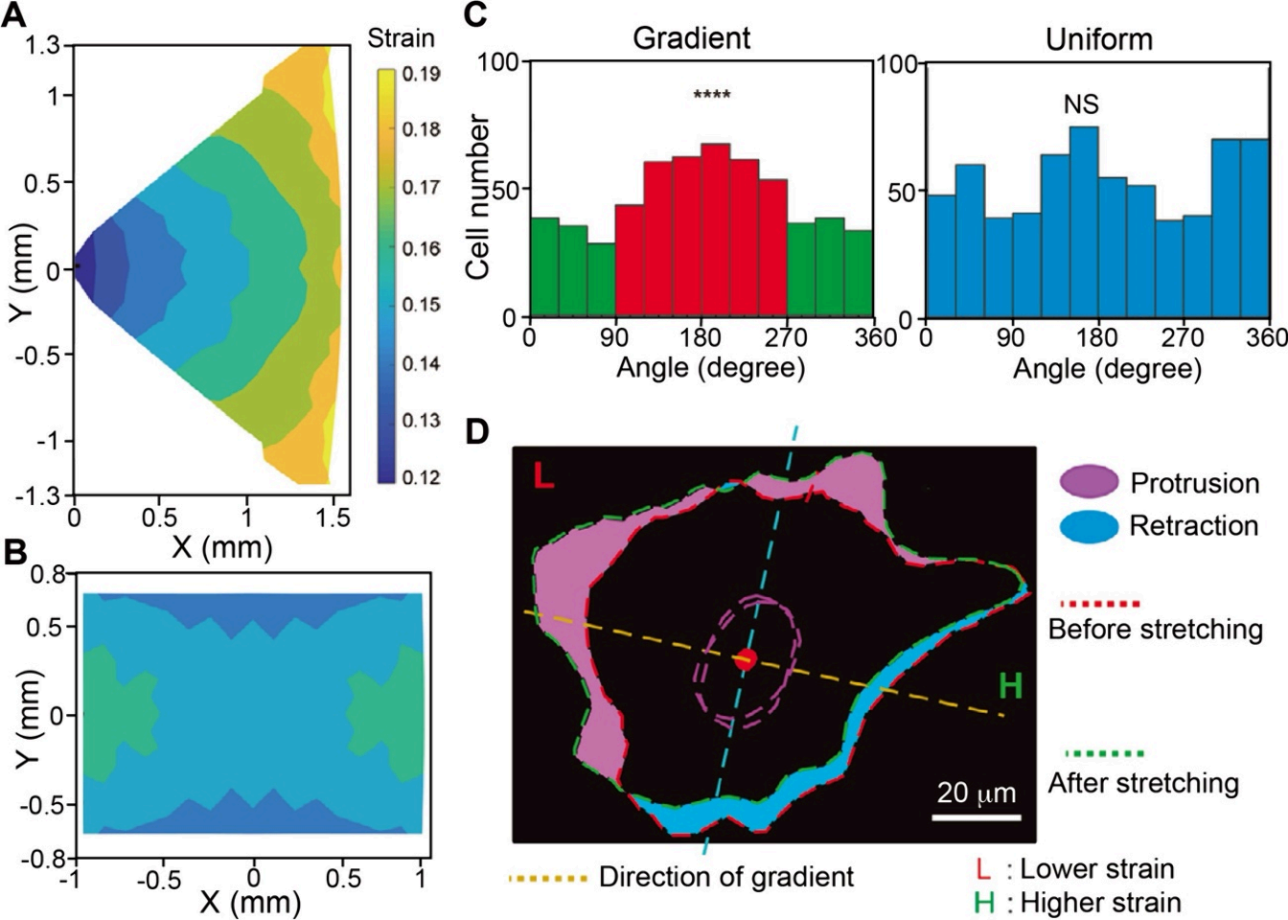
Directional cell migration in response to a gradient in strain. Strain field map showing experimental calibration of (A) graded and (B) uniform distributions of strain. (C) Histograms of cell migration angles under a static gradient in strain (left) and uniform strain (right). Under the graded condition, cells exhibited a preferential migration toward the region of lower strain (****p < 0.0001), whereas no directional migration was observed under uniform strain. NS: no significant difference. (D) Representative image of a single cell before and after stretching, showing the formation of a polarized protrusion (purple) on the side experiencing lower strain and retraction (blue) on the side under a higher strain. Dashed lines indicate cell boundaries pre- and poststretching; the graded direction is marked in yellow. Reproduced with permission from ref 199. Copyright 2023 Wiley-VCH.

Physical cues derived from surface topography also play a critical role in guiding directional cell migration. Aligned electrospun nanofibers present directional contact guidance, promoting polarized morphology and directional migration. For example, NIH-3T3 fibroblasts exhibited significantly enhanced axial migration on uniaxially aligned PCL nanofibers compared to flat tissue culture substrates.^37^ Similarly, radially aligned nanofibers facilitated centripetal migration of dural fibroblasts toward the scaffold center, unlike random fibers, which failed to induce such coordination.^200^ Beyond binary comparisons, topographical gradients, such as a gradual transition from random to aligned nanofibers, have also been explored to fine-tune cell migration behavior.^201^ The graded architectures resemble the anisotropic microenvironments found in native tissues such as tendon-to-bone insertions. As cells migrate across regions with increasing fiber alignment, they experience stronger contact guidance cues accordingly, which in turn enhances both their efficiency and directionality of migration.

Besides mechanical and physical cues, biochemical gradients offer an additional means to direct cell migration via free or surface-bound molecular signals. Specifically, chemotaxis is a form of directional cell migration in response to gradients of free signaling molecules such as growth factors or cytokines. The mode of migration relies on ligand–receptor signaling cascades initiated by concentration differences across the cell membrane, enabling cells to orient and move toward higher concentrations of attractants.^202^ A notable example was demonstrated using fibrous scaffolds loaded with a growth factor.^203^ In this case, the epidermal growth factor (EGF) was encapsulated in microparticles of a phase-change material (PCM), together with indocyanine green as a photothermal trigger. The particles were sandwiched between layers of radially aligned and random nanofibers. Upon near-infrared (NIR) light exposure, the PCM underwent a solid-to-liquid transition, releasing EGF in a spatially controlled manner. The graded release, along with the radial alignment of the nanofibers, significantly enhanced the radial migration of fibroblasts. This work also demonstrates how a stimuli-responsive system can be used to generate spatiotemporally controlled gradients to direct the chemotactic migration of cells.

Haptotaxis refers to the directional migration of cells in response to immobilized gradients of adhesion molecules or matrix-bound biochemical cues.^204^ Unlike chemotaxis, which involves gradients of free signaling molecules, haptotaxis is governed by cell interaction with substrate-bound ligands such as fibronectin, collagen, or laminin.^205^ Electrospun nanofibers offer a versatile platform for integrating topographical alignment with biochemical gradation. Surface coating with adhesion proteins like fibronectin enhances cell-fiber interaction, amplifying migratory responses. Beyond a simple uniform coating, the use of graded surface-bound ligands has been explored to establish directional tropism. In one study, collagen or collagen-fibronectin nanoparticles were deposited on the nanofibers in a 2D gradient.^50^ Schwann cells or NIH-3T3 fibroblasts could be directed to migrate toward regions of increasing ligand density, demonstrating that a surface-bound biochemical gradient can also effectively guide cell migration in the absence of soluble signaling molecules. Such haptotactic systems are particularly useful in regenerative medicine.

Functionally graded surfaces and materials provide a powerful and versatile strategy for guiding cell migration by emulating the spatial heterogeneity of native tissue microenvironments. Through the integration of mechanical (e.g., stiffness and strain), physical (e.g., topography), and biochemical (e.g., chemotactic and haptotactic) cues, the graded systems allow for a tight control of cellular behavior. Both 1D and 2D gradients have been shown to enhance migration efficiency, alignment, and tissue-specific responses *in vitro*, offering promise for biomedical applications in wound healing, nerve regeneration, and interface tissue engineering.

### 4.2 Control of Organoid Development

Organoids are self-organized 3D tissues typically derived from pluripotent, fetal, or adult stem cells.^206^ They recapitulate many aspects of the structure and function of the corresponding *in vivo* tissues. Therefore, they have been used as models for studying the fundamental mechanisms and processes of the development, regeneration, and repair of human tissues. They have also been widely used for diagnostics, disease modeling, drug discovery, and personalized medicine.^207^ The graded distributions of signaling molecules in a biomaterial or 3D scaffold play a pivotal role in organoid development.^208^ The gradients are instrumental in organizing cells into complex structures during embryogenesis and tissue regeneration. A good example can be found in morphogens: signaling molecules that establish a gradient in concentration across tissues or cellular fields, enabling cells to adopt distinct fates in a dose-dependent manner.^10,11^ By recapitulating gradient-based bioactive cues in engineered systems, researchers can guide organoid development to emulate the architectural and functional complexity of native tissues or organs, enhancing their physiological relevance as model systems.

In one study, Göpfrich and co-workers used DNA hydrogel microbeads containing morphogen molecules (e.g., Wnt agonist) to generate a gradient in morphogen within an organoid.^209^ After microinjection into an organoid, the microbeads would gradually breakdown upon UV irradiation to release the Wnt agonist (Figure 32A), leading to the formation of a graded distribution of Wnt agonist from the center toward the periphery of the spheroid (Figure 32B). Directly increasing the concentration of Wnt agonists in a medium would induce retinal pigmented epithelium formation in the retinal organoid while heavily suppressing neuroretinal differentiation. In contrast, the graded distribution of Wnt agonist in the retinal organoid could induce the retinal pigmented epithelium formation without suppressing retinal ganglion cells, leading to the development of a better retinal organoid (Figure 32C,D). The trend was a direct result of the internal Wnt agonist gradient in the organoid, which decreased from the center to the periphery. As such, the neuroretinal cells at the rim were exposed to a low Wnt agonist concentration, a condition opposite to that of standard cultures, where homogeneously supplemented medium exposes the cells to the highest Wnt agonist level.

**Figure 32:**
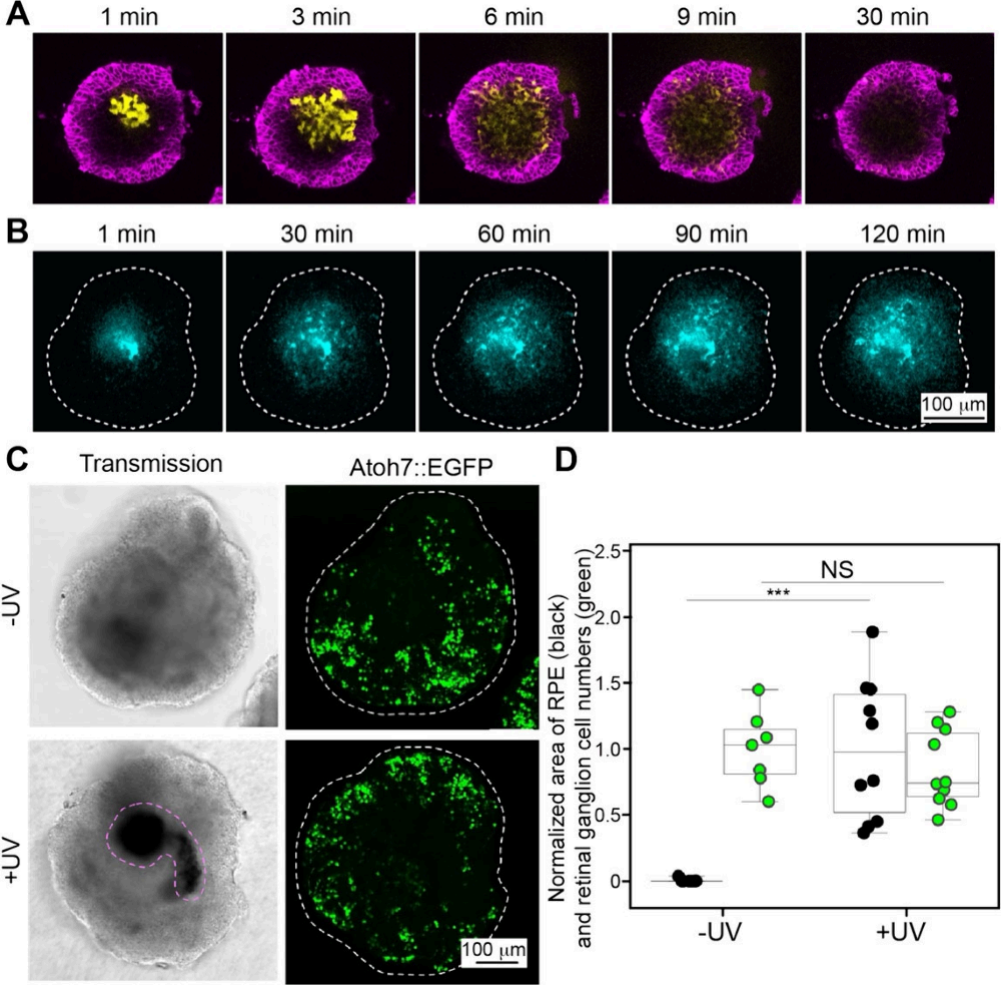
Biological responses of the retinal organoid in response to the gradient of Wnt agonist delivered by DNA microbeads. (A) Representative time-lapse confocal micrographs of DNA microbeads (yellow) after microinjection and subsequent breakdown of the microbeads in the retinal organoid, with the retinal organoid being counterstained with live plasma membrane stain (magenta). (B) Representative time-lapse confocal micrographs of Wnt agonist tagged with Alexa Fluor 647 (Wnt-AF647) after release from the DNA microbeads in one retinal organoid. (A) and (B) share the same scale bar. (C) Representative confocal transmission and maximum intensity Z-projection images of retinal organoid at day 4 after microinjection of the DNA microbeads and then release of Wnt agonist at day 1. (D) Quantitative analysis of retinal pigmented epithelium (black) area and retinal ganglion cell (green) numbers from (C), demonstrating spatially graded retinal ganglion cell development in response to the Wnt-surrogate gradient. Reproduced with permission from ref 209. Copyright 2024 the Author(s) (CC BY 4.0).

### 4.3 Repair or Regeneration of Interfacial Tissues

As mentioned in the Introduction, interfacial tissues are situated at “soft-to-hard” tissue interfaces. Soft tissues, such as ligament, tendon, and cartilage, connect and support bodily components, whereas hard tissues (e.g., bone) shape the body and enable locomotion through mechanical strength. Interfacial tissues, such as cartilage-to-bone, tendon-to-bone, and bone-to-ligament insertions, connect the soft and hard tissues, and are critical for joint motion and stabilization.^210^ The interfacial tissues are characterized by diverse gradations in mineral content, cell phenotypes, and interdigitation of collagen fibers, to facilitate the dissipation of stresses across the interface.^211^ The complex interfacial tissues are difficult to regenerate after injury. Surgically reconnecting the soft and hard tissues tends to suffer from high failure rates due to structural and mechanical mismatches between the soft and hard tissues.^212^

Tissue-engineering approaches based on functionally graded materials hold great promise for promoting the repair or regeneration of interfacial tissues.^213^ The graded materials offer gradients in composition, structure, as well as physical and biological cues, to regulate the proliferation, migration, and differentiation of stem cells for the repair or regeneration of damaged interfacial tissues.^213^ For example, multiple types of graded materials based on HAp have been designed for inducing the graded osteogenic differentiation of stem cells and tendon-to-bone insertion repair. As the primary mineral component of bone, HAp has been widely used to regulate the osteogenic differentiation of stem cells and osteogenesis due to its superior osteoinductivity. In one study, Xia and co-workers fabricated a graded HAp/PCL scaffold featuring a continuous gradient in HAp content via swelling-induced diffusion.^100^ The resulting scaffold had a sandwich-like structure, consisting of a top PCL layer, a middle HAp-graded zone, and a bottom layer with a fixed HAp concentration. To promote cell seeding and migration, an array of funnel-shaped channels (ca. 200 μm in diameter, ca. 100 μm center-to-center spacing) was machined using CO_2_ laser.

After seeding into the scaffold, adipose-derived stem cells (ASCs), a promising source of MSCs capable of differentiating into multiple cell lineages, were seeded into the channels, with a uniform distribution along the channels. The osteogenic differentiation of ASCs within the graded scaffold was assessed by evaluating the expression of alkaline phosphatase (ALP) and osteocalcin (OCN). ALP is an enzyme expressed in the early stage of differentiation into osteoblasts. At the same time, OCN is a bone ECM protein expressed by osteoblasts and plays an essential role during embryonic osteogenesis and bone remodeling. As a result, the activity of ALP and expression of OCN can be considered as early and late indicators of osteogenesis, respectively. After 7 and 14 days of osteogenic differentiation, the scaffold was cryo-sectioned and assessed with a commercial ALP staining kit. Both the fluorescence micrographs in Figure 33A and the quantitative analysis of the fluorescence intensities in Figure 33B demonstrated that there was a graded expression of ALP across the transition zone, which correlated with the HAp content. The OCN immunostaining data in Figure 33C,D confirmed a graded OCN expression along the channels, indicating that the gradient in HAp content stimulated local osteogenesis of ASCs, generating a graded distribution of cell phenotypes and promoting the formation of a tendon-to-bone-like interface.

**Figure 33:**
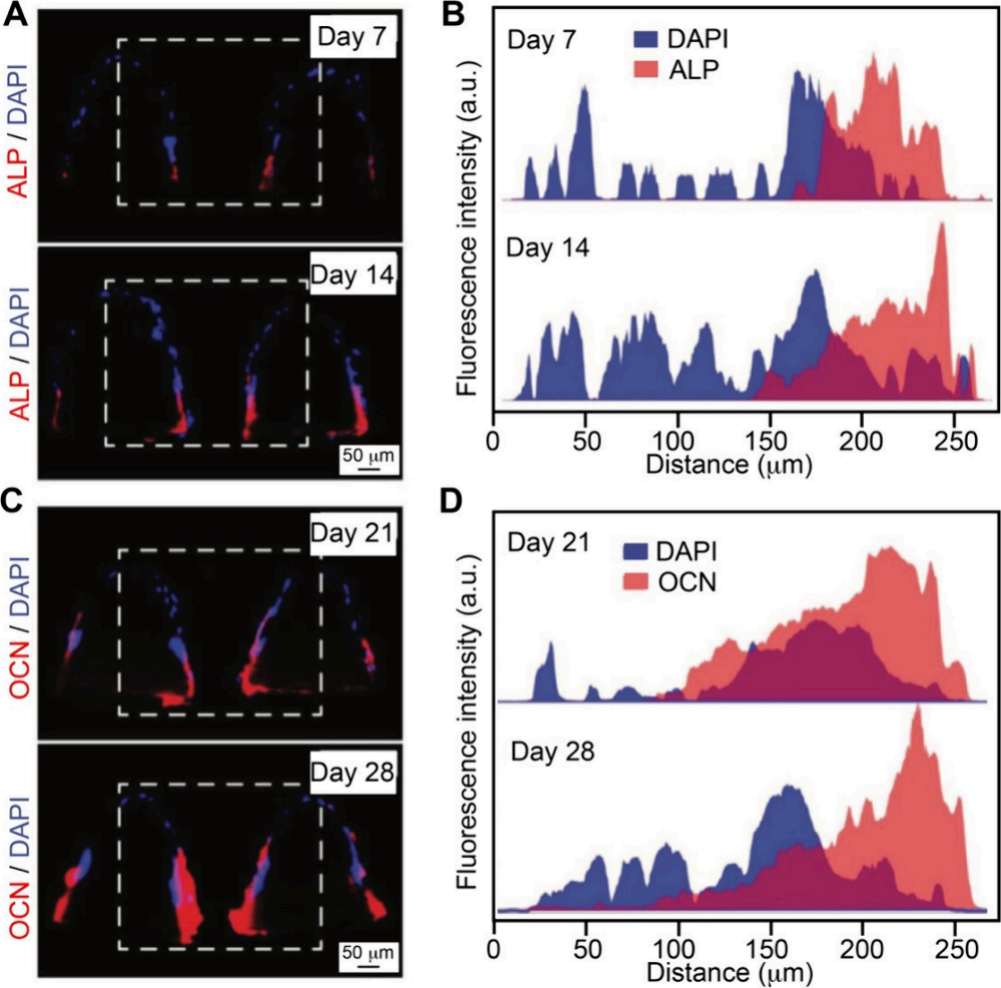
Osteogenic differentiation of ASCs in the HAp-graded scaffold. (A) Cross-sectional fluorescence micrographs showing ASCs in the scaffold after 7 and 14 days of osteogenic differentiation. ALP and cell nuclei were stained to give red and blue fluorescence, respectively. (B) Quantitative analysis of ALP expression along the channel corresponding to the images in (A). (C) Cross-sectional fluorescence micrographs showing ASCs in the scaffold after 21 and 28 days of osteogenic differentiation. OCN and cell nuclei were stained to give red and blue fluorescence, respectively. (D) Quantitative analysis of OCN expression along the vertical direction. Reproduced with permission from ref 100. Copyright 2022 Wiley-VCH.

Building upon the successful fabrication of HAp-graded scaffolds for directing osteogenic differentiation, a biological effector, Hedgehog agonist (HhAg), was also incorporated into the scaffolds to replicate natural enthesis development for the recreation of functional tendon-to-bone attachment (Figure 34A).^214^ To ensure the homogeneous distribution and enhance the trackability of HAp, uniform nanorods synthesized in-house were used instead of the nanoparticles obtained from a commercial source. Subsequently, funnel-shaped microchannels were also machined in the scaffolds with gradients in HAp and HhAg (HAp+HhAg) in the same manner as described above. Human-derived MSCs were then seeded into the scaffolds and cultured for 21 days to evaluate the combined effect of HAp and HhAg gradients in driving spatially graded stem cell differentiation. To assess differentiation, osteopontin (OPN), collagen type X (Col-10), and scleraxis A (SCXA) were used as markers for osteogenic, chondrogenic (hypertrophic), and tenogenic cell phenotypes, respectively. Confocal micrographs taken at three sections along the microchannel depth demonstrated that the synergistic effect of both effectors indeed generated a gradient of the three cell phenotypes (Figure 34B). Quantitative analysis of fluorescence intensity further elucidated the spatial distribution (Figure 34C). Specifically, in the dual-graded scaffold, SCXA expression was the highest at the top section, Col-10 expression initially increased before decreasing toward the bottom, and OPN expression was the greatest at the bottom section. The trend contrasted sharply with the scaffolds containing a single gradient in HAp content, which showed a graded cell osteogenic differentiation only. The observed trends indicate that as the content of HhAg and HAp increased, cell phenotypes transitioned seamlessly from tenocytes to mineralized (hypertrophic) chondrocytes and then to osteoblasts, mimicking the pattern observed in developing and functional tendon-to-bone insertion.

**Figure 34:**
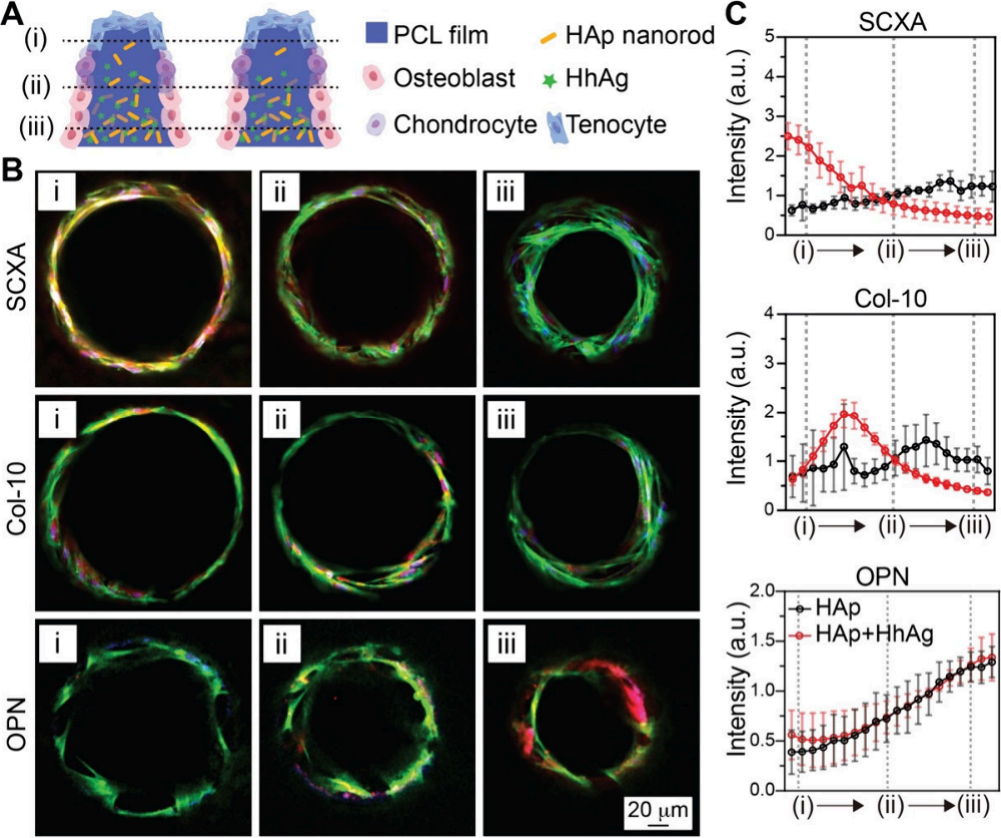
Graded differentiation of MSCs seeded in the scaffold with dual gradients in HAp and HhAg. (A) Schematic showing different sections of the scaffold. (B) Fluorescence micrographs showing the graded differentiation of MSCs in the channels of the dual-graded scaffolds. (C) Plots of the fluorescence intensities of the differentiation markers, SCXA (tenogenic), Col-10 (chondrogenic), and OPN (osteogenic), when moving from the top (*i*) to the bottom (*iii*) of the channels. Reproduced with permission from ref 214. Copyright 2025 the Author(s) (CC BY 4.0).

In an independent study, Stevens and co-workers successfully induced cartilage-to-bone transition using a scaffold featuring a gradient of growth factor.^119^ To establish the gradient, they employed a buoyancy-driven method to distribute BMP-2, a key osteoinductive factor, in decreasing concentration in a matrix of cross-linked gelatin methacryloyl and heparin methacryloyl. During the initial casting process, MSCs were homogeneously distributed within the polymer network. The entire construct was then cultured for 28 days in a medium capable of supporting both osteogenesis and chondrogenesis. Subsequently, the formation of osteochondral tissue was evaluated using histological staining. As shown by the Alizarin Red S staining result in Figure 35A, localized mineralization occurred exclusively at the end of the scaffold with the highest concentration of BMP-2. In contrast, sulfated glycosaminoglycans, a key ECM component present in both cartilage and bone, showed uniform distribution across the scaffold (Figure 35B). The histological results were further corroborated by immunofluorescence staining, which revealed that the osteogenic marker OPN was present on the bone-designated end of the scaffold (Figure 35C). Taken together, the engineered constructs exhibited distinct bone and cartilage regions, demonstrating the potential use of this versatile platform for interfacial tissue engineering.

**Figure 35:**
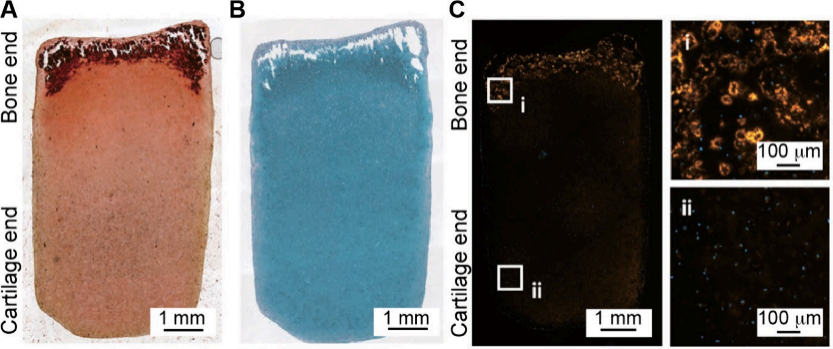
Osteochondral differentiation of MSCs seeded in a scaffold with a gradient of growth factor. (A) Optical micrographs of Alizarin Red S staining indicating localized mineral deposition. (B) Optical micrographs of Alcian Blue staining indicating the distribution of glycosaminoglycans. (C) Fluorescence micrographs of OPN staining after culturing MSCs with the graded scaffold for 28 days. Reproduced with permission from ref 119. Copyright 2019 the Author(s) (CC BY 4.0).

As for the *in vivo* applications in the repair or regeneration of interfacial tissues, the usage of graded scaffolds will be different depending on whether the gradient is on the surface or in the bulk. For example, Thomopoulos, Xia, and co-workers fabricated a nanofiber mat with graded HAp content on the surface and used it as a patch over the repair site for tendon-to-bone interface repair (Figure 36A).^215^ The graded patch can mimic the natural gradient in mechanical and biochemical properties of enthesis to enhance biological integration by providing distinct microenvironments for different types of cells. For instance, the mineral-rich region adjacent to the bone can promote osteoblast activity and mineralization, while the softer, polymer-dominated region near the tendon could support tenocyte proliferation and collagen synthesis (Figure 36B). The spatial control over cellular behavior is critical for regenerating the functionally graded ECM characteristic of the tendon-to-bone interface. In addition, the mechanical compatibility of a graded patch could reduce stress concentrations at the repair site. However, the surface-graded patch could only regulate the biological activity of cells and tissues that were in contact with the patch, which would compromise its performance in repairing the whole interface. Moreover, the patch often needs to be fixed to the repair site through sutures or adhesives, which may introduce additional trauma or harm to the tissues.

**Figure 36:**
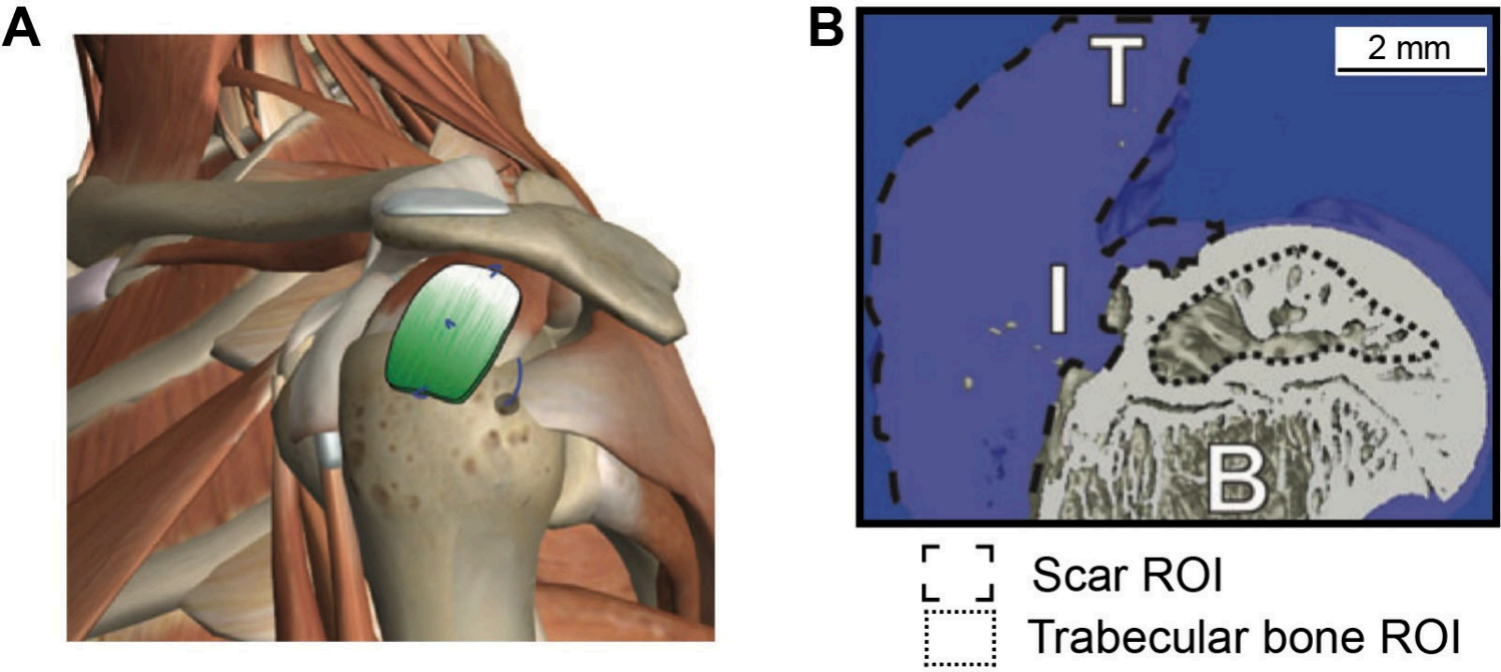
PLGA electrospun nanofiber mat with a gradient in mineral content on the surface and its use as a patch for tendon-to-bone repair. (A) Schematic illustration showing the human shoulder patched with a mineral-graded nanofiber mat. (B) 3D reconstruction of the repaired attachment of a rat, in which B, I, and T represent bone, insertion, and tendon, respectively. Reproduced with permission from ref 215. Copyright 2015 Mary Ann Liebert.

In comparison, the scaffold with mineral gradation in the bulk may circumvent some surface-related issues by supporting the gradient throughout the scaffold, thereby offering more robust mechanical integration and reducing the risk of delamination. In one demonstration, Deng and co-workers fabricated a graded hydrogel involving bimetallic ions for the repair of tendon-to-bone insertion (Figure 37A).^216^ Specifically, copper ion-based thiolate gelatin hydrogel (s-Cu-gelation) and zinc ion-based thiolate gelatin hydrogel (s-Zn-gelation) were fabricated by mixing thiolate gelatin with copper and zinc ions, respectively, through the S–Cu or S–Zn coordinative cross-linking. Afterward, the s-Cu-gelation and s-Zn-gelation hydrogels were attached to each other. Due to the diffusion of the ions across the interface and the dynamic cross-linking capacity of metal–thiolate, graded distributions of the two metal ions would be formed in the hydrogel. Figure 37B shows the EDX mapping images recorded from the cross-section of the freeze-dried sample, revealing graded distributions of both copper and zinc elements. The dual-graded hydrogel was then implanted in a rat model with a rotator cuff defect to evaluate tendon-to-bone interface regeneration. At week 4, compared with the pure suture group, the rats implanted with gelatin hydrogel and graded s-Cu/Zn-gelatin hydrogel showed less infiltration by inflammatory cells, as well as more regular morphology and arrangement of tendon tissue (Figure 37C). At week 8, the graded s-Cu/Zn-gelatin group showed a more organized collagen distribution and a significant increase in tendon maturation. In addition, the s-Cu/Zn-gelatin hydrogel also showed a larger fibrocartilage area than in the suture repair and s-gelatin groups (Figure 37D). The results indicate that the dual-graded hydrogel is more favorable for the repair of rotator cuff tears. In the process of fibrocartilage regeneration, cartilage regeneration and ingrowth were induced under a copper microenvironment, while tenocytes were recruited under a zinc microenvironment. In the dual-graded hydrogel system, zinc and copper ions not only acted as cross-linkers but also provided strong antibacterial effects to promote regenerative capacity *in vivo*.

**Figure 37:**
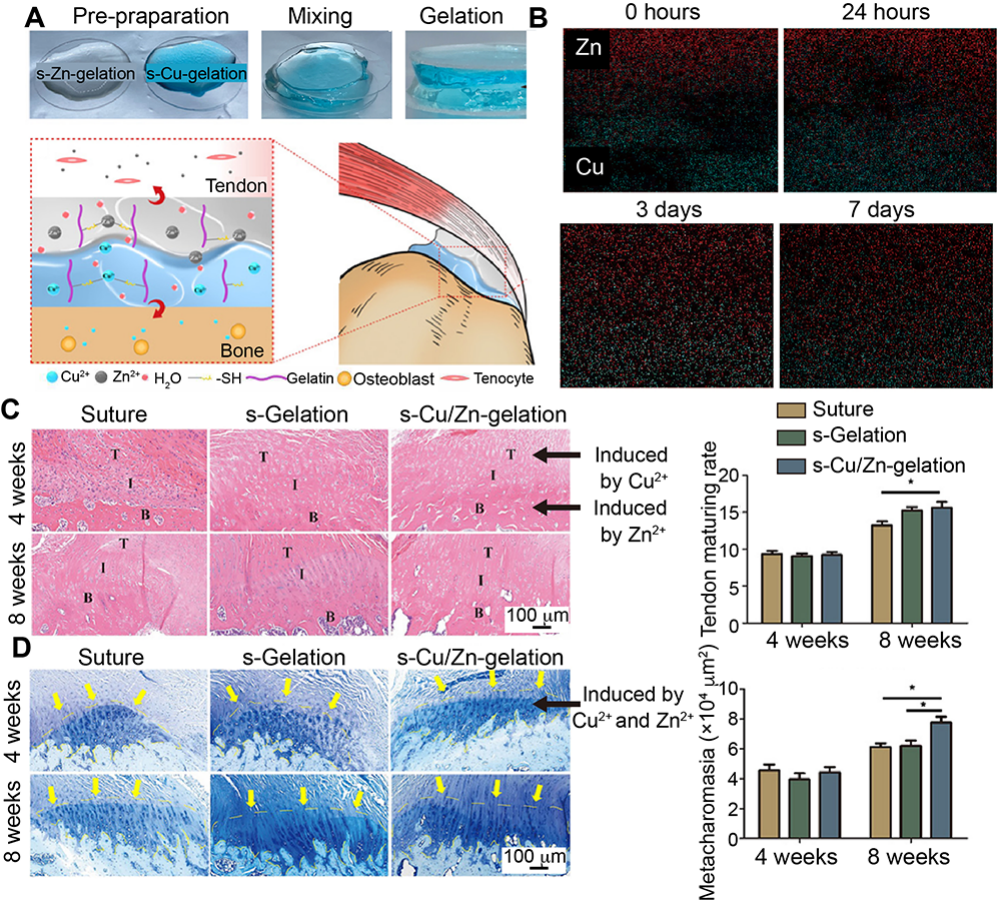
Fabrication and implantation of dual-graded hydrogels for the regeneration of tendon-to-bone insertion in a rat model. (A) Photographs showing the preparation of the dual-graded hydrogel and a schematic showing its implantation at the tendon-to-bone interface. (B) EDX mapping recorded from the cross-section of a dual-graded hydrogel after freeze-drying. (C) Optical micrographs of hematoxylin and eosin-stained tissue sections from rats treated with suture only (suture), thiolation gelatin hydrogel (s-gelatin), and the dual-graded hydrogel (s-Cu/Zn-gelatin), along with the corresponding tendon maturation scores for each treatment group (*n* = 3). T, tendon; I, interface; B, bone. (D) Optical micrographs of toluidine blue-stained tissue sections from rats in different treatment groups, along with quantification of the area of newly formed fibrocartilage. (*n* = 3). *p < 0.05. Reproduced with permission from ref 216. Copyright 2015 the Author(s) (CC BY-NC 4.0).

In addition to tendon-to-bone interface regeneration, functionally graded materials have also been used in osteochondral defect repair. For example, Cai and co-workers developed a multilevel-graded hydrogel for the repair of full-thickness osteochondral defects (Figure 38A).^62^ The graded hydrogel was comprised of a double-network matrix of gelatin methacryloyl and acrylated β-cyclodextrin and incorporated with superparamagnetic HAp (MagHA) nanorods. Under a magnetic field, the MagHA nanorods moved downward vertically in the prehydrogel solution to generate an incremental gradation, followed by fixation through hydrogel cross-linking. The resulting hydrogel possessed the top-to-bottom gradients in MagHA content and Young’s modulus that depended on the duration of exposure to the magnetic field (Figure 38B-D). Therefore, the hydrogel had a gradient like the cartilage-to-bone interfacial tissue in terms of mineral content and mechanical stiffness. The graded hydrogel was then implanted in a rabbit model of full-thickness osteochondral defects. At 12 weeks postsurgery, the defect treated with the graded hydrogel exhibited the most complete tissue integration and cartilage regeneration compared with biphasic hydrogel or hydrogels without MagHA nanorods (Figure 38E). The findings indicate that the hydrogel with gradients in mineral content and mechanical properties exhibits good regenerative performance by recapitulating the structural and functional anisotropy of native osteochondral tissue.

**Figure 38:**
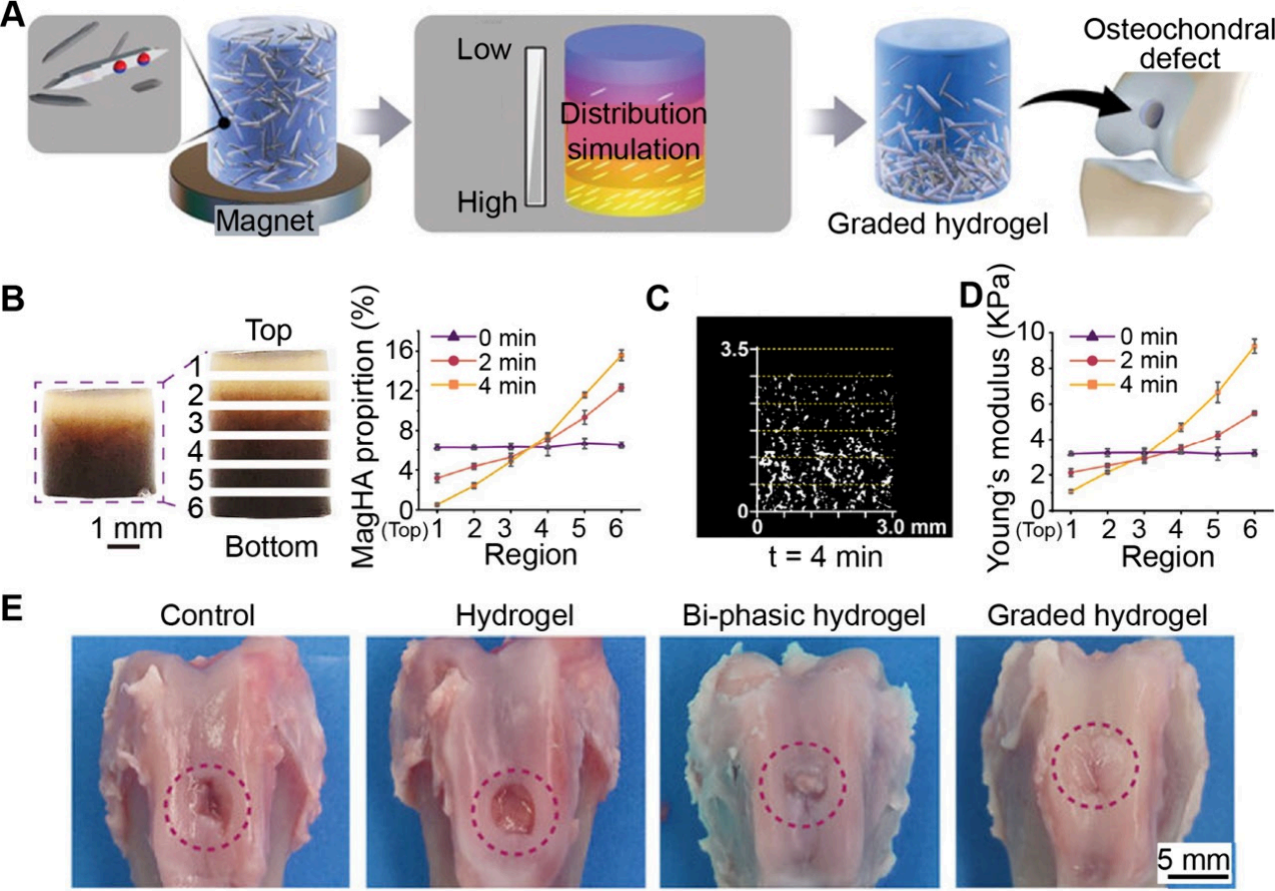
Fabrication of graded hydrogels for the repair of osteochondral defects. (A) Schematic showing the design and fabrication of the graded hydrogel. (B) Gross appearance of the graded hydrogel after exposure to a magnetic field for 4 min and thermogravimetric analysis of the different parts. (C) Micro-CT image of the graded hydrogel. (D) Young’s modulus of the different regions of the graded hydrogel. (E) Gross images of the repaired osteochondral defects in the various treatment groups. Reproduced with permission from ref 62. Copyright 2023 Wiley-VCH.

### 4.4 Neural Regeneration

Neural regeneration has gained increasing attention due to the high prevalence of neural injuries, which frequently result in the loss of motor coordination, sensory function, and independence.^217^ In most cases, the neural injuries involve both structural and functional damage to neurons. A typical neuron consists of a soma (cell body), an axon, and dendrites.^218^ The axon plays a crucial role in transmitting electrical impulses over long distances, enabling neurons to communicate with one another and respond to stimuli. As such, the effective neural regeneration requires not only the survival of neurons but also the outgrowth and extension of axons. During neural development, axonal extension can be orchestrated by growth cones that are located at the leading edges of neurites.^219^ Acting as the sensory and motile machinery at neurite tips, growth cones navigate extracellular environments by sensing various guidance factors, including biochemical gradients, mechanical signals, and topographical features. Among the various guidance factors, the biochemical gradients of neurotrophic factors such as NGF and glial cell line-derived neurotrophic factor are shown to promote the extension of neurites and influence their pathfinding direction.^220^ In particular, the graded distributions of these molecules offer chemotactic cues to enable more robust and directional guidance for neurite extension compared to uniform presentations.^221,222^ In addition to various biochemical cues, the oriented physical topographies, such as uniaxially- or radially aligned nanofibers, can provide structural guidance to elongate neurites by mimicking the anisotropic architecture of native neural tissues.^223^ Altogether, it is crucial to combine the gradients of biochemical factors with physical topographical features for effective neural regeneration.

As discussed in Sections 2 and 3, a variety of fabrication techniques have been developed to create gradients on the surface or in the bulk of a material. A representative example involves the construction of a 2D surface gradient in NGF on a mat of electrospun PCL nanofibers.^224^ The construct integrated a radially decreasing gradient in NGF with fiber alignment, generating both chemotactic and topographic guidance cues. As a result, the neurites extending from DRG explants grew significantly longer along the outward direction (following the increasing NGF concentration) compared to those extending inward toward the center (against the concentration gradient). The synergistic combination of biochemical and topographical cues highlights the potential of such a graded scaffold to promote neurite outgrowth. Another example involved the use of electrosprayed particles made of a mixture of collagen and laminin to generate a 1D surface gradient in particle density along the direction of uniaxially aligned nanofibers.^37^ When DRG explants were cultured on the resulting mat, the neurites exhibited significantly enhanced outgrowth in the direction of increasing particle density. The trend also demonstrated the synergistic effect of biochemical gradient and topographic cue in guiding neurite extension *in vitro*.

In the native neural microenvironment, cells interpret and respond to spatial cues presented in all three dimensions, including gradients in biochemical factors, mechanical stiffness, and topographical architecture.^224^ To recapitulate the complexity, there is an increasing demand for the development of scaffolds that integrate the biochemical gradients with oriented microstructures. In one demonstration, Luo and co-workers fabricated a graded scaffold featuring both an NGF gradient and aligned microchannels through a combination of 3D printing and directional freezing (Figure 39A).^225^ Specifically, a customized printer equipped with dual reservoirs—one containing NGF-free and the other NGF-loaded silk fibroin/collagen (SF/Col) solutions—was used to mix and inject the inks into a cylindrical mold. By inversely modulating the flow rates of the two inks, a bulk gradient in NGF concentration was established along the longitudinal axis of the scaffold. Subsequent directional freezing preserved the gradient within an array of oriented microchannels. The neuroinductive performance of the scaffold was evaluated using DRG explants cultured in four groups: *i*) scaffolds with randomly oriented microchannels (RS), *ii*) scaffolds with uniaxially oriented microchannels (OS), *iii*) scaffolds with a uniform distribution of NGF along the oriented microchannels (U-NGF+OS), and *iv*) scaffolds with graded NGF along the oriented microchannels (G-NGF+OS). As shown in Figure 39B, oriented topography alone promoted neurite alignment, and the incorporation of NGF gradient further enhanced neurite extension, demonstrating the synergistic effect of structural and biochemical cues on neurite outgrowth. To advance the clinical translation of the technologies for peripheral nerve repair, it is essential to demonstrate the efficacy of combining oriented topographical features with biochemical gradients *in vivo*. As such, the fabricated 3D graded scaffold was integrated into an electrospun nanofiber-based nerve conduit to facilitate nerve suturing and then implanted into a 15 mm sciatic nerve defect *in vivo*. At 6 weeks postsurgery, the cross-section of the midnerve segment was immunostained for NF200 (green) and myelin basic protein (red), which serve as markers for the regenerated axons and myelin sheaths, respectively (Figure 39C). The expression of both NF200 and antimyelin basic protein was enhanced in the scaffolds with oriented microchannels, particularly for those incorporating an NGF gradient. The results demonstrated that a combination of NGF gradient and oriented microstructures can enhance axonal regeneration and functional recovery *in vivo*.

**Figure 39:**
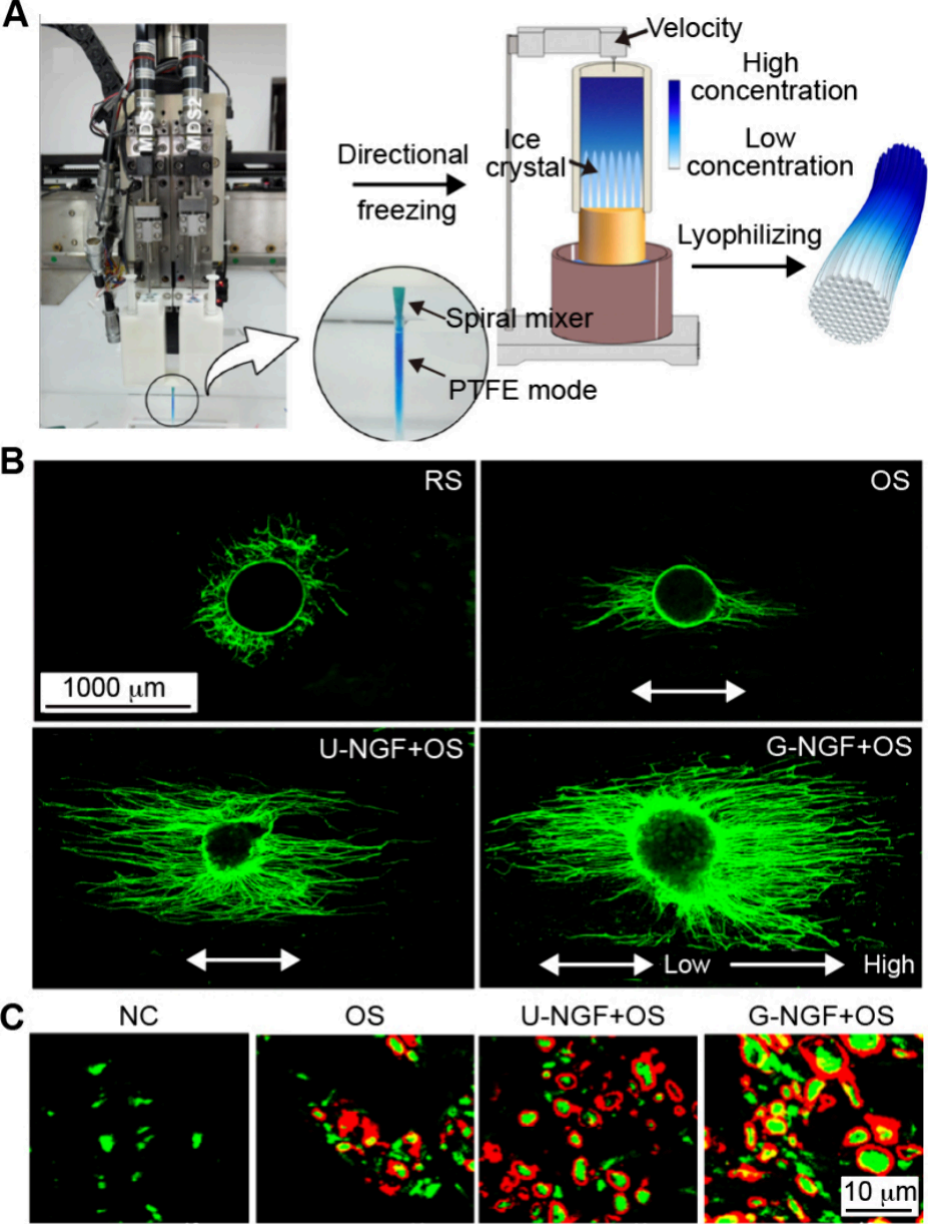
Application of graded scaffolds for neural regeneration. (A) Schematic showing the fabrication of a scaffold featuring a biochemical gradient along the microchannels. Methylene blue was used to visualize the gradient. (B) Fluorescence micrographs of DRG explants cultured for 3 days on various types of scaffolds and stained for β-tubulin III. The double arrow indicates the orientation of the microchannels, while the single arrow indicates the direction of elevated NGF concentration. (C) Fluorescence micrographs showing the regenerated axons stained with NF200 (green) and the myelin sheaths stained with myelin basic protein (red). Reproduced with permission from ref 225. Copyright 2020 American Chemical Society.

The gradient formed in a microfluidic system has also been utilized to model early human neural tube development. For instance, Kirkeby and co-workers developed a microfluidic cell culture system capable of generating a molecular gradient, in which embryonic stem cells responded to a Wnt-activating gradient, leading to progressive caudalization from forebrain to midbrain and then hindbrain.^226^ The resulting *in vitro* models offer a powerful tool to explore previously inaccessible aspects of human development and disease mechanisms. While most studies have relied on single 1D gradients, one notable example employed a four-channel microfluidic device to generate two orthogonal gradients,^227^ enabling the spatial patterning of collagen-embedded embryonic stem cells into motor neuron lineages.

Overall, the design of graded scaffolds for neural repair or regeneration has focused on the incorporation of different bioactive cues. Once implanted, the scaffold can effectively integrate with the host neural microenvironment to maximize its therapeutic benefits for repairing both the peripheral and central nervous systems.

### 4.5 Cardiovascular Tissue Engineering

The cardiovascular system consists of the heart and a closed system of vessels, including arteries, veins, and capillaries, that deliver blood throughout the body. According to the World Health Organization, cardiovascular disease accounts for 32% of global deaths, causing ca. 18 million deaths annually.^228^ As such, there is a strong interest in developing tissue engineering scaffolds, such as vascular grafts, myocardial scaffolds, and artificial heart valves, to replace or regenerate damaged cardiovascular tissues. A key limitation of the conventional cardiovascular implants is their inability to replicate the structural and functional complexity of native tissues. Typically, the heart and blood vessels do not have uniform mechanical and biochemical properties; instead, they exhibit gradual transitions in stiffness, composition, and cellular organization. Most conventional synthetic grafts and polymeric scaffolds lack the graded properties, leading to thrombosis, restenosis, inflammation, and graft failure events. As such, functionally graded materials have emerged as a promising biomimetic approach in cardiovascular tissue engineering, allowing for spatially controlled variations in mechanical, biochemical, and topographical properties.

#### 4.5.1 Cardiac Tissue Engineering

Native cardiac tissue is structurally and functionally anisotropic, comprising aligned cardiomyocytes, conductive pathways, and a gradually varying mechanical stiffness across different layers.^229^ The organization is critical for the synchronized contraction and efficient electrical conduction required for heart function. The myocardium exhibits regional differences in mechanical stiffness, electrical conductivity, and cellular alignment across its thickness, from the outer epicardial layer, through the midmyocardium, to the inner endocardium. The variations result in a transmural gradient in fiber orientation, typically following a helical or spiral pattern that facilitates effective torsional contraction (Figure 40A).^230^ Replicating the intricate myocardial architecture is essential for developing functional cardiac tissue constructs.

**Figure 40:**
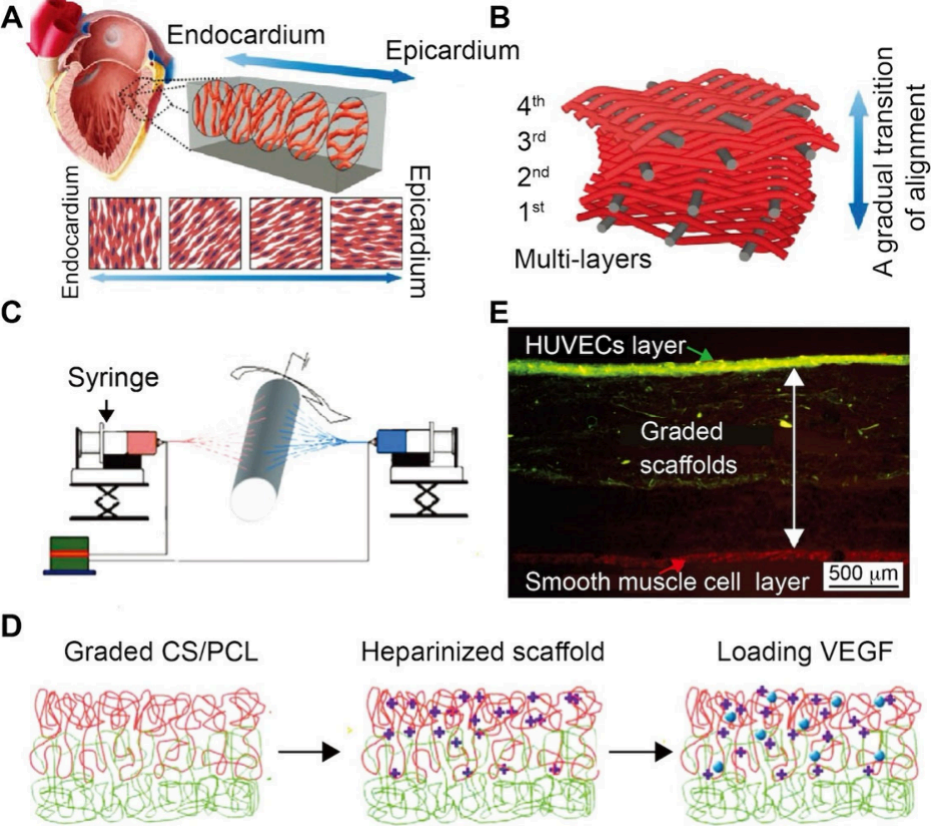
Fabrication of graded scaffolds for vascular tissue engineering. (A) Schematic of native myocardial architecture showing the transmural gradient in cardiomyocyte alignment from endocardium to epicardium. (B) Schematic of the graded scaffold with layer-specific orientation to replicate the graded anisotropic structure of vascular tissue. (C) Schematic of sequential coelectrospinning setup used to fabricate nanofibrous scaffolds with compositional gradients. (D) Fabrication of a graded vascular scaffold. (E) Cross-sectional fluorescence micrograph of the graded vascular scaffold, showing HUVECs adhesion on the luminal surface and suppressed smooth muscle cell migration in the abluminal region, confirming spatial control over cellular responses. (A, B) Reproduced with permission from ref 230. Copyright 2017 American Chemical Society. (C, D) Reproduced with permission from ref 237. Copyright 2012 Elsevier.

Wu and co-workers developed a multiscale, graded scaffold by integrating 3D printing with electrospinning to mimic the complex, graded architecture of native cardiac tissue.^231^ Their approach involved the creation of microscale frames with parallel filament structures through 3D printing, onto which aligned electrospun nanofibers were deposited, forming a well-controlled directional microenvironment. By adjusting the diameter and spacing of the printed filaments, the researchers successfully modulated nanofiber alignment, thereby developing a three-layer, multiscale scaffold with a gradual transition in nanofiber orientation. The graded scaffold effectively guided cardiomyocyte alignment and maturation to mimic the anisotropy of native myocardium. The structural guidance significantly enhances synchronous beating behavior, holding promise for improved cardiac tissue repair. In another work, Ma and co-workers fabricated a graded composite scaffold by weaving aligned conductive nanofiber yarns with surgical sutures into an interwoven network, which was subsequently encapsulated in a hydrogel matrix.^230^ As shown in Figure 40B, the resulting scaffold provided spatially organized and graded structural cues, enabling cardiomyocytes to align neatly, elongate effectively, and undergo enhanced maturation and synchronous beating. The graded, anisotropic architecture not only supported cardiomyocyte growth on individual scaffold layers but also allowed for distinct control over orientation across different layers, replicating the natural, multilayered myocardial structure. Such graded scaffolds hold promise for effectively guiding myocardial tissue regeneration by tightly controlling cell alignment and enhancing cardiac functionality.

Beyond fiber orientation, graded mechanical environments also influence the function of cardiomyocytes. For example, Corbin and co-workers fabricated a dynamic magnetorheological elastomer scaffold with gradients in mechanical stiffness to mimic the stiff-to-soft transitional region observed in postmyocardial infarction remodeling.^232^ The cardiac fibroblasts cultured on the scaffold with a gradient in stiffness exhibited spatially dependent behaviors: cells in stiffer regions displayed increased spreading area, elevated α-smooth muscle actin expression, and higher secretion of fibrotic markers such as TGF-β, compared to the cells in softer regions. Significantly, dynamic mechanical softening effectively mitigated fibroblast activation and fibrosis-related gene expression, underscoring the potential of mechanically graded scaffolds for investigating pathological remodeling mechanisms and guiding therapeutic strategies for cardiac tissue repair. Expanding upon the theme of biochemical gradients, Odedra and co-workers fabricated porous collagen scaffolds with gradients of immobilized vascular endothelial growth factor (VEGF) to control endothelial cell migration.^233^ They established a radial gradient in VEGF concentration across the scaffold using a straightforward point-source immobilization method to mimic the natural tissue environment. The resulting scaffold directed endothelial cell migration preferentially toward the center, significantly enhancing cell infiltration and distribution in the interior region relative to the control group with uniformly immobilized VEGF. This work demonstrates that the gradients of growth factors in a scaffold can effectively guide cell migration, holding promise for improved tissue integration and vascularization in engineered cardiac tissues.

#### 4.5.2 Vascular Tissue Engineering

While cardiac tissue engineering requires scaffolds with graded anisotropic electrical and mechanical properties to match the contractile function, vascular tissue engineering calls for scaffolds with a different but equally complex set of requirements. Blood vessels are comprised of multilayered structures with gradually varying elasticity, biochemical composition, and cellular phenotype.^234^ They contain a thick outer layer made of connective tissue; a thicker middle layer consisting of circularly arranged elastic fibers, connective tissue, and smooth muscle cells; and the thinnest inner layer comprised of a single layer of endothelium.^235,236^ The intrinsic gradients are instrumental in regulating blood flow, preventing thrombosis, and supporting vascular remodeling. As with cardiac tissue engineering, conventional vascular grafts often fail to reproduce the graded transitions, leading to compliance mismatch, intimal hyperplasia, or graft occlusion. To overcome the challenges, functionally graded vascular scaffolds have emerged as a promising approach to mimic the native vessel architecture and promote long-term graft integration.

Zhang and co-workers developed a graded nanofibrous scaffold consisting of chitosan (CS) and PCL to replicate the graded microenvironment of native blood vessels.^237^ Using a sequential coelectrospinning method (Figure 40C), they created a graded scaffold in which the proportion of CS nanofibers gradually increased from the adventitial layer toward the luminal surface. The scaffold was subsequently heparinized to enhance anticoagulant capability and functionalized with VEGF (Figure 40D) for controlled release of VEGF. The gradation allowed for sustained release of VEGF and spatially guided HUVECs proliferation and monolayer formation on the luminal surface, while inhibiting vascular smooth muscle cell proliferation on the abluminal side (Figure 40E). The graded scaffold thus effectively mimics natural vascular ECM, supporting endothelial regeneration and reducing thrombosis risks, making it promising for vascular tissue engineering applications.

Functionally graded materials hold great potential in addressing the structural and functional limitations of conventional scaffolds for cardiovascular engineering. Native cardiac and vascular tissues possess inherent spatial heterogeneities in terms of stiffness, alignment, conductivity, and biochemical composition, which are essential for their physiological function. By integrating spatial control over multiple factors, such as structural, mechanical, and biochemical cues, graded scaffolds provide a versatile platform for engineering cardiovascular tissues with enhanced functionality and long-term performance.

### 4.6 Wound Management

As the largest organ of the human body, the skin serves as a dynamic and multifunctional barrier, protecting against microbial invasion while regulating water retention, thermoregulation, and sensory perception.^238^ When the barrier is disrupted due to injury or disease, a wound is formed, exposing the underlying tissue to external stressors.^239^ Without the protection of skin, the wound bed becomes highly susceptible to microbial contamination, significantly increasing the risk of infection. In the absence of prompt and effective intervention, the wound may evolve into a chronic, nonhealing state, presenting challenges for clinical management.^240^ Bioactive gradient-laid dressings have emerged as a promising strategy for wound management. By mimicking the gradients in native tissue, including spatial variations in mechanical stiffness, structural architecture, and biochemical signals, gradient-laid dressings can effectively direct critical cellular processes, such as migration, proliferation, and re-epithelialization.^241^

Typically, the design of an effective scaffold requires a comprehensive understanding of the multidimensional gradients of native skin, which encompass physical (e.g., stiffness, porosity, topography), chemical (e.g., growth factor distribution), and ionic (e.g., Ca^2+^) parameters.^242^ To successfully replicate the natural wound healing microenvironment, it is critical to rationally select the biomaterials and fabrication techniques. A compelling example is the work by Wu and co-workers, who engineered a fibroblast-loaded artificial dermis with a “sandwich” architecture.^243^ Using a layer-by-layer method, they fabricated a three-layer scaffold featuring a gradient in pore size. The larger pores in the outer layers can enhance the formation of granulation tissue, while a denser middle layer provides mechanical stability and prevents the scaffold from rapid degradation. Compared to its uniform counterpart, the gradient-laid scaffold showed robust cell proliferation, enhanced re-epithelialization, and accelerated wound closure. Through a tight control of the thermal gradient, Han and co-workers also fabricated alginate-based scaffolds with a continuous gradient in pore size.^244^ The resulting scaffold showed a 1D gradient in pore size from the bottom to the top, effectively mimicking the natural dermis-to-epidermis transition in skin. The structural gradient not only promoted cell migration but also enhanced nutrient diffusion, offering a promising platform for wound management. Notably, the solvent-free fabrication method underscores the versatility of thermal manipulation in producing graded scaffolds for advanced wound management. To further address the challenges of wound closure in a fluid-rich and dynamic environment, Li and co-workers developed a tissue adhesive patch with gradient in mechanical properties, which was composed of three key components: *i*) a tissue-adhesive hydrogel matrix for strong bonding to wet tissue surface, *ii*) a micromesh with graded mechanical properties to protect the wound from dynamic mechanical stress, and *iii*) an oil-infused surface to prevent unwanted adhesion with the surrounding tissues.^241^ The multifunctional patch achieved robust sealing and dynamic wound closure under a fluid-rich environment, allowing the injured tissue to deform naturally with a minimal stress concentration.

While the graded physical cues, such as pore size and stiffness, have proven effective for wound management, their combination with biochemical signaling (e.g., chemokines or proteins), especially for the recruitment of endogenous stem cells, offers a promising strategy for enhancing wound healing.^245^ It is well-established that the stromal cell-derived factor-1α (SDF-1α) plays a pivotal role in guiding cell migration through specific interaction with CXCR4 receptors on MSCs.^246^ Scaffolds with a graded concentration of SDF-1α have been explored to promote directional stem cell migration for effective wound management. Drawing inspiration from the radial venation pattern of the royal water lily and its remarkable mechanical stability (Figure 41A), Ding and co-workers fabricated a radially aligned nanofiber patch using a specially designed collector.^247^ The biomimetic design incorporated dual gradients—a radial gradient in fiber density coupled with another gradient in SDF-1α concentration—to collectively direct stem cell migration toward the center of the wound (Figure 41B). Due to the degradation of gelatin methacryloyl in response to the upregulated matrix metalloproteinase-9 (MMP-9) within the inflammatory microenvironment of a wound defect, the patch was further coated with gelatin methacryloyl to enable “on-demand” release of the anti-inflammatory drug diclofenac sodium (DS).

**Figure 41:**
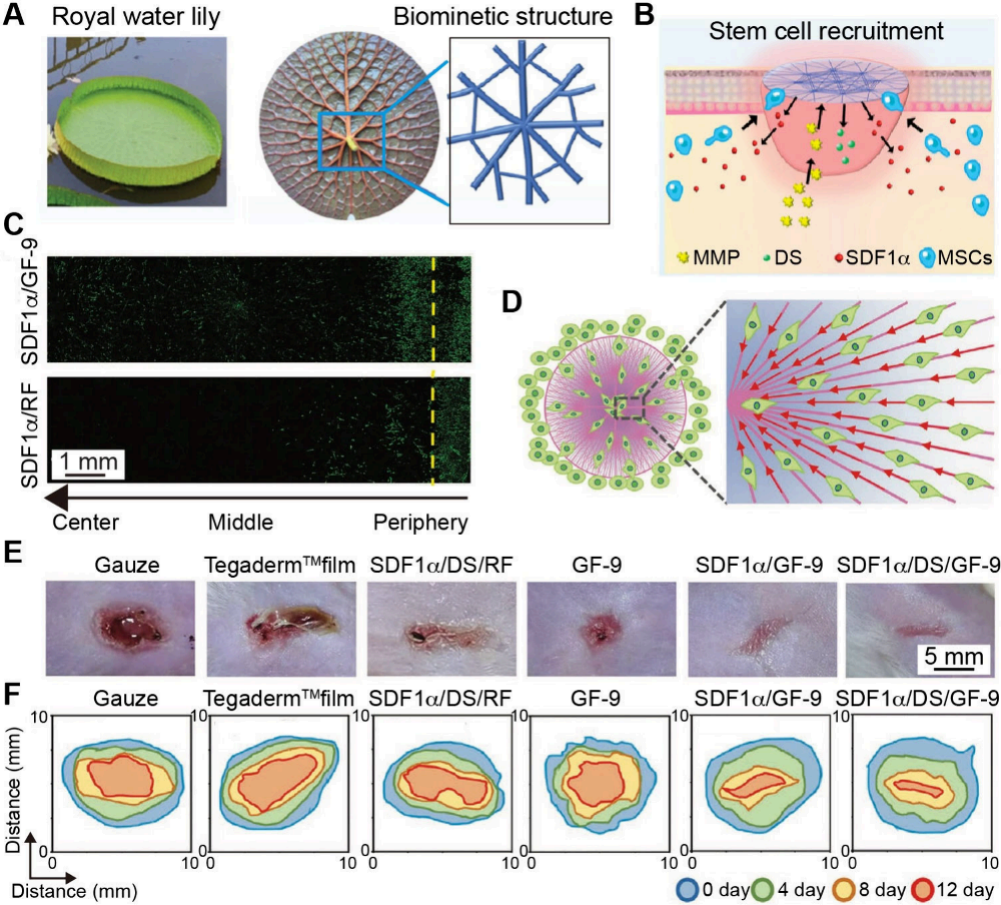
Application of graded nanofibrous patches for accelerating wound healing. (A) Photograph of a royal water lily and the graded structure. (B) Schematic showing the graded patch with a radial architecture, enabling mechanical support and controlled release at the wound site. DS was released in response to MMP-9 to mitigate inflammation, while the gradient of SDF-1α guides stem cell migration toward the wound center, collectively promoting wound healing. (C) Fluorescence micrographs showing the distribution of MSCs on different patches after 7 days of culture. The dashed line indicates the boundary between the seeding zone and the migration zone. The GF-9 patch was fabricated through electrospinning. (D) Schematic illustrating the synergistic MSC migration induced by the aligned nanofibers and gradient of SDF-1α. (E) Photographs of wound sites after 12 days postsurgery. (F) Schematic showing the reduction in wound area over time across different treatment groups. Reproduced with permission from ref 247. Copyright 2021 Wiley-VCH.

To evaluate cell migration, MSCs were selectively seeded at the periphery of the patch with the assistance of a stainless-steel ring to mimic peripheral healthy skin tissue, whereas the void in the center simulated a wound defect. After 7 days of culture (Figure 41C), the random-oriented patch with uniformly distributed SDF-1α (SDF1α/RF) exhibited limited cell migration, with only a few cells migrating from the periphery to the center. In contrast, the radially aligned patch featuring a graded distribution of SDF-1α (SDF1α/GF-9) demonstrated significantly enhanced cell migration, with MSCs actively moving from the periphery to the central region. As illustrated in Figure 41D, the improved migratory behavior resulting from the synergistic effect of aligned fiber-induced topological guidance and the chemotactic SDF-1α gradient directs MSC migration toward the center of the wound. Next, the wound healing efficacy of the patch was evaluated using a mouse full-thickness skin incision model. As shown in Figure 41E, the radially aligned patches with graded SDF-1α distribution (SDF1α/GF-9 and SDF1α/DS/GF-9 groups) significantly accelerated wound closure compared to the conventional gauze and the commercial Tegaderm film. Notably, the patches capable of codelivering SDF1α and DS (SDF1α/DS/GF-9 group) achieved the most pronounced healing effect among all groups. Consistent with these observations, wound area traces over time revealed that patches incorporated with both SDF-1α and DS exhibited the most rapid wound closure (Figure 41F), particularly, during the critical early inflammatory phase. The results highlight the synergistic therapeutic effect arising from the topographical guidance of the radially aligned nanofibers, chemotactic gradient of SDF-1α, and inflammation-responsive drug release from the hydrogel coating, collectively contributing to the enhanced wound repair.

Taken together, the rational design and implementation of gradients in bioactive dressings are advancing wound management. The approach requires strategic integration of physical gradients (e.g., layered porosity or radial fiber alignment) with biochemical gradients (e.g., chemokine or growth factor distribution) to recreate a regenerative microenvironment akin to preparing fertile “soil” for wound repair. When combined with appropriate stem cell therapies, such gradient-laid scaffolds are able to orchestrate cellular migration, modulate inflammatory microenvironments, and accelerate wound closure, offering a promising strategy for advanced wound repair.

### 4.7 Drug Screening

A multitude of new drugs are continually being developed to address unmet needs in various fields. The process of drug discovery typically starts with screening, during which appropriate candidates are selected from an extensive library of lead compounds against the specific therapeutic target. High-throughput screening can significantly improve the efficiency of drug discovery. Current assays, including 2D cell monolayers, 3D cell culture models, and *in vivo* animal models, still face distinct limitations. Despite their accessibility and affordability, 2D cell models on plastic culture plates poorly recapitulate the intricate and heterogeneous physiological environment, particularly the spatially varying properties of the ECM, including its organization, stiffness,^248^ biochemical cues,^249^ and cellular components.^119^ The significant simplification greatly limits their predictive validity. Conversely, *in vivo* animal models, while offering greater complexity to replicate the physiological environment, are hampered by high cost, considerable time investment, and ethical concerns that restrict throughput. Most importantly, interspecies differences often undermine the translation of findings from animal platforms to human clinical outcomes.^250,251^ Functionally graded materials, on the other hand, enable the creation of advanced 3D *in vitro* models that can better mimic physiological complexity through controlled spatial variations in their properties, bridging the translational gap in drug screening.

Functionally graded materials offer two significant advantages for drug screening: *i*) improved recapitulation of the heterogeneity of the physiological environment of the disease site, enhancing the clinical relevance of the screening outcomes, and *ii*) significant increases in experimental efficiency and information density, facilitating higher-throughput screening capabilities. Specifically, graded materials enable systematic investigations of cellular behavior across a continuous spectrum of microenvironmental conditions, including factors such as matrix elasticity, ligand presentation, and nutrient availability, all integrated within a single experimental platform. In one study, Yang and co-workers developed a graded hydrogel system for screening the effect of matrix stiffness on glioblastoma multiforme, one of the most common forms of brain cancer.^252^ To generate the graded material, a graded generator was used to mix two solutions containing 3 and 7 wt % PEG hydrogel precursors, respectively, with the proportion of 7 wt % PEG precursor solution gradually increased as the experiment proceeded. The resultant solution, with increasing PEG precursor concentration, was then pumped into a mold and polymerized under UV light. To improve the physiological relevance of the screening platform, the range of stiffness of the hydrogel was designed to match that of the brain, varying from 40 to 1300 Pa. Compared with conventional studies that employed multiple discrete hydrogels with varied formulations to study brain cancer cell behavior, the graded hydrogel matrix enabled efficient screening across a continuous range. Brain cancer cells were incorporated into the hydrogel matrix by suspending them in the initial PEG precursor solutions. Immunostaining and matrix metalloproteinase expression results showed that the brain cancer cells preferentially attached to softer matrices, as the less-dense matrices facilitated easier infiltration and remodeling. Notably, cells in stiffer regions showed an increased resistance to treatment, suggesting that matrix stiffness could directly modulate cellular behavior and influence treatment outcomes. Without graded materials, such crucial insights of context-dependent therapeutic effects would be masked or averaged out in traditional, homogeneous culture systems.

Graded materials can also be engineered to model other niche biological gradients. For example, many drug screening platforms have been developed using microfluidics to study the cellular effects of steep physicochemical gradients at tumor-host interfaces.^253^ A good example is the construction of structures equivalent to *in vivo* skin for toxicity, cosmetic, and pharmaceutical testing.^254,255^ The organotypic model replicates the three compositional layers of skin: a hypodermis, an artificial dermis with fibroblasts and plasma, and a stratified keratinocyte-containing epidermis. After seeding with stem cells, these model systems can serve as a versatile platform for realistic *in vitro* tests on drug absorption.

Furthermore, as discussed in Section 4.3, graded materials offer advantages in replicating the zonal organization of interfacial tissues, such as the osteochondral tissues. Grunlan and co-workers prepared a single hydrogel scaffold with graded properties mimicking the interfacial tissue. The platform not only enabled rapid screening of cell-material interactions,^256^ but also potentially serves as a scaffold to facilitate osteochondral tissue repair.

Beyond improving biological fidelity, drug screening approaches based on functionally graded materials also offer significant benefits in terms of experimental efficiency and the density of information obtained. By integrating a range of stimuli—for instance, a continuous gradient in drug concentration or systematically varying substrate topographies—into a single platform, the graded materials are particularly well-suited for multiplexed assays.^257,258^ The simultaneous testing of multiple conditions within the same culture environment significantly reduces well-to-well or sample-to-sample variation when comparing discrete experimental conditions. Such a platform also streamlines workflows, minimizing reagent usage, manual handling, and overall experimental time. Using a combinatorial hydrogel with multiple biochemical gradients, for example, Burdick and co-workers achieved high-throughput screening of cellular microenvironments.^259^ Specifically, they modified HA hydrogels with a norbornene group (NorHA), enabling the formation of linkages with monothiolated peptides through light-mediated reactions (Figure 42A). By controlling the UV light exposure with a movable opaque mask, graded peptide concentrations (0–5 mM) were achieved on the surface of the NorHA hydrogel (Figure 42B). Particularly, to replicate cell–cell and cell-matrix interactions, single and orthogonal gradients of Arg-Gly-Asp (RGD) sequences and His-Ala-Val (HAV) motifs were incorporated into the hydrogel. The gradients could be visualized using fluorescent tags, as shown in Figure 42C. With MSCs photoencapsulated in the hydrogel, the authors investigated how the levels of these interactions synergistically influenced MSC chondrogenesis, using Sox9 and Aggrecan as chondrogenic markers (Figure 42D,E). The hydrogel platform featuring spatially varying biochemical formulations allowed for the derivation of optimal RGD and HAV combinatorial concentrations for promoting chondrogenesis, which were further validated on discrete hydrogels. This approach allows one to readily alter the graded steepness and/or map different biochemical components, and it is also adaptable to combinations of other factors.

**Figure 42:**
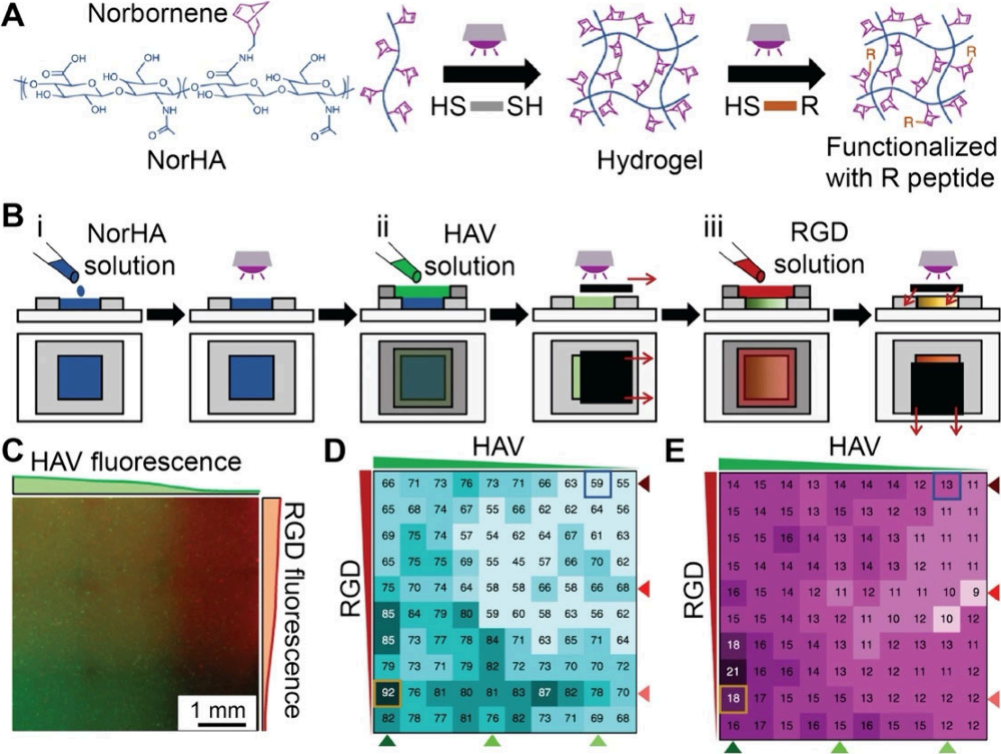
Combinatorial hydrogels with biochemical gradients for screening 3D cellular microenvironments. (A) Schematic showing the fabrication of the hydrogel with a single peptide gradient. (B) Fabrication of a dual-graded hydrogel: *i*) preparation of the hydrogels in a mold via a thiol-norbornene UV light-mediated reaction between NorHA and dithiol cross-linker; *ii*) incubation with a 5 mM monothiolated HAV peptide solution and generation of the HAV gradient with a horizontally sliding opaque mask to control the extent of light-mediated reaction between HAV peptides and norbornenes in the hydrogel; and *iii*) incubation with a 5 mM monothiolated RGD peptide solution and generation of the secondary HAV gradient with a sliding opaque mask in the orthogonal direction. (C) Fluorescence micrographs of rhodamine-labeled RGD and fluorescein-labeled HAV orthogonal gradients, including intensity profiles on each side. (D) Effect of the orthogonal HAV and RGD gradients on transcription factor Sox9 expression. (E) Effects of the orthogonal HAV and RGD gradients on Aggrecan synthesis. Reproduced with permission from ref 259. Copyright 2018 the Author(s) (CC BY 4.0).

### 4.8 Soft Actuators

Inspired by the efficient, intricate movements found in biological systems, soft actuators are emerging as an active area of research. Notably, the remarkable working of nature, from the powerful contractions of cephalopod tentacles to the subtle seismonastic movements of *Mimosa pudica* leaves, is enabled by the functional gradients within their structures. Mimicking these gradients holds the key to developing next-generation soft actuators for applications ranging from delicate robotic gripping to fluid-based switches, soft electronics, and bionic robots.^260,261^

Achieving the nature-equivalent complexity presents a significant challenge. Conventional actuators, typically fabricated from single, uniform materials, face fundamental limitations in both performance and versatility. They are often limited by a trade-off between force output and response speed and tend to produce only simple, predictable motions like uniform bending or twisting. Creating more complex and/or programmable motions with these materials requires cumbersome external control systems or complex graded stimuli that are challenging to implement and maintain, thereby reducing the efficiency and autonomy of the soft actuators. While multilayered actuators have been explored to overcome some of the limitations, they often suffer from stress concentrations at the sharp interfaces between dissimilar materials, leading to delamination and unsatisfactory performance. Functionally graded materials, on the other hand, provide an efficient way to resolve these issues. By programming spatial variations in chemical composition, mechanical properties, and macro/microscopic structures directly into a single actuator body, it is possible to achieve complex, preprogrammed motions in response to one global stimulus.

Soft actuators driven by gradients in their mechanical properties serve as a good example of how functionally graded materials are applied to actuation. For instance, Menges and co-workers fabricated a cellulose-based soft actuator with a continuous, multidirectional gradient in mechanical properties by 3D printing.^262^ The gradient was created by modulating the composition of the extruded fibers. A higher concentration of lignin, a natural reinforcing agent derived from plant cell walls, resulted in increased stiffness and tensile strength of the printed material. In contrast, the incorporation of citric acid diminished both mechanical characteristics. The stiffness of the material was also controlled geometrically by varying the thickness of the extruded fibers, with thicker fibers creating regions of higher stiffness. The gradient in stiffness effectively guided the deformation of the printed material, enabling complex behaviors and redistribution of forces in response to the actuation force.

In addition to mechanical properties, gradients in chemical composition provide another route to complex, autonomous actuation. Hydrogel-based systems, for instance, can be synthesized with a preprogrammed gradient in their swelling ratio.^263^ When exposed to a global stimulus like water, different regions of the hydrogel swell to varying degrees, generating internal stresses that drive a 2D-to-3D shape transformation. As a result, a simple, flat material can execute sophisticated actuation, autonomously morphing into a 3D functional structure like a delicate flower petal or a gripping claw. As a key challenge, many of these passive systems are plagued by their slow response speed. To accelerate actuation, researchers have embedded a graded distribution of magnetically, photonically, or electrically responsive components within the material matrix. The components include magnetic nanoparticles (e.g., SPIONs or MnFe_2_O_4_),^264,265^ light-responsive metallic nanorods,^266^ and metal ions.^267^ This approach creates a gradient of responsiveness to an external magnetic or electric field, enabling a tight control over the final actuated shape. For example, Podstawczyk and co-workers fabricated soft actuators that morphed into different preprogrammed shapes under a uniform magnetic field by varying the concentration of magnetic fillers in their 3D printing ink.^268^

Alternatively, the internal architecture of an actuator can be engineered with structural gradients to create anisotropic motion. For example, Chen and co-workers fabricated a hydrogel with a well-controlled gradient in porosity to achieve rapid, programmable locomotion.^269^ The gradient was created by polymerizing NIPAM with a heterobifunctional cross-linker (4-hydroxybutyl acrylate) under hydrothermal conditions, which caused PNIPAM–OH to precipitate out and form a gradient from top to bottom (Figure 43A). Subsequent intermolecular dihydroxylation yielded a hydrogel with a continuous gradient in porosity (Figure 43B). After loading the porous network with polypyrrole-based photothermal transducers, the material could execute a variety of complex motions, including bending, twisting, and octopus-like swimming under NIR laser irradiation. A swelling/deswelling gradient was generated in response to porosity changes, enabling rapid thermal responses and directional locomotion. As shown in Figure 43C, under constant laser irradiation at a fixed spot, the graded hydrogel strip could lift a mass of 700 mg, demonstrating its potential as a photomechanical converter for artificial muscles and soft robotics.

**Figure 43:**
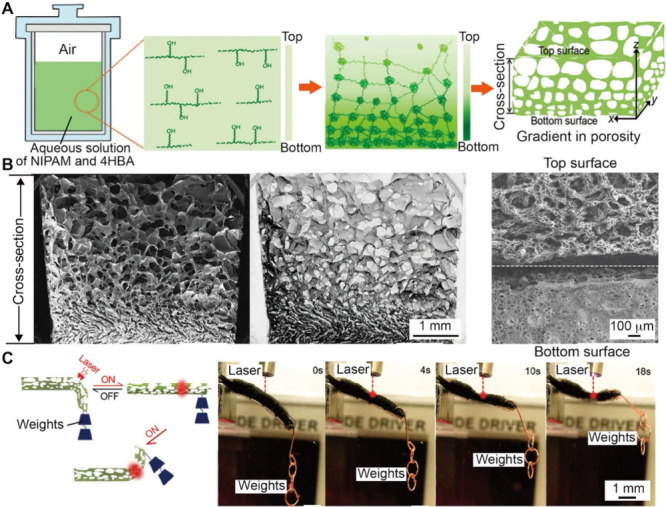
Hydrogels with a gradient in porosity for soft actuation. (A) Schematic showing the fabrication of a graded porous hydrogel. (B) Cross-sectional SEM images of the hydrogel with a gradient in porosity. (C) Schematic and photographs showing the use of the hydrogel to lift weights upon laser irradiation. Reproduced with permission from ref 269. Copyright 2015 Wiley-VCH.

Ultimately, the rational design and fabrication of functionally graded materials are critical for advancing soft actuators from simple, single-motion devices to sophisticated, functional systems capable of complex tasks. The continued development of graded materials holds promise for creating the next-generation smart actuators, which will, in turn, enable revolutionary advances in diverse fields such as adaptive robotics, biomedical devices, artificial muscles, and dynamic materials that can autonomously reconfigure their shape and function.

Taken together, while both functionally graded surfaces and materials utilize gradients for advanced functionality, their primary biomedical applications are fundamentally distinct, rooted in their dimensionality and intended functions. Specifically, the functionally graded surfaces are typically applied as coatings or modifications to existing substrates. Their primary role is to control and guide events at the surface by presenting spatially varying cues, such as gradients in proteins, minerals, or stiffness, to modulate specific cellular responses like adhesion, migration, and differentiation. As these applications often involve modifying existing medical devices or creating platforms for *in vitro* assays, functionally graded surfaces are generally closer to direct clinical or diagnostic use. In contrast, functionally graded materials are primarily developed as bulk, three-dimensional scaffolds intended to replace or regenerate entire tissue sections. These materials feature gradients in composition, porosity, and mechanical properties throughout their volume to recreate the complex, hierarchical architecture of native tissues. Their fundamental applications are in regenerative medicine, including the creation of scaffolds for tendon-to-bone regeneration, the engineering of triphasic plugs for osteochondral defects, and the fabrication of biomimetic structures for bone engineering. These ambitious goals position functionally graded materials predominantly in the preclinical research and development stage, where the focus is on achieving long-term, functional tissue integration. For clarity, the major biomedical applications, strengths, limitations, technology readiness/clinical status, and translational barriers of functionally graded surfaces and materials are outlined in Table 3.

**Table 3:** Overview of Functionally Graded Surfaces and Materials for Biomedical Applications^a^

Category	Biomedical Application	Primary Clinical Goal	Dominant Gradient Variable(s)	Strengths	Limitations	Technology Readiness Level	Major Translational Barriers
Functionally graded surfaces	Dental implants	Soft-tissue seal; osseointegration; biofilm resistance	Roughness; wettability; ligand/ion density	Zone-specific behavior with minimal bulk change; clinical precedent	Biofilm challenge; multienvironment wear/corrosion; aesthetics	7–9 (widespread)	Cross-vendor quality control durability of antibacterial chemistries
	Cardiovascular stents (luminal coatings)	Hemocompatibility; rapid endothelialization; antirestenosis	Ligand/NO donors; drug dose; wettability; nano/microtopography	Mechanics-neutral; precise axial dosing; retrofit	Cracks during crimp/expand; dose profile drift in flow	7–9 (in clinic)	Fatigue under pulsatile load; shelf life; reproducible axial dosing; coating quality control
	Neural regeneration (luminal coatings)	Axon alignment; localized neurotrophic factor delivery	Groove depth/pitch; factor density; charge/wettability	Adds cues without altering tube mechanics; compatible with common polymers	Limited drug loading; coating fragility; effect diminishes over long gaps	4–6 (preclinical/early devices)	Sterilization and shelf life for bioactive coatings; long-gap efficacy
	Biosensors	High sensitivity/selectivity; stable baseline; antifouling; rapid response	Ligand/antibody density; antifouling polymer density (PEG/zwitterionic); conductivity/doping; catalytic sites; micro/nanotopography	Surface-controlled capture; multiplexing via spatial gradients; reduced fouling; retrofit on existing transducers	Biofouling and signal drift over time; delamination under flex; sterilization/solvent sensitivity	7–9 for *in vitro* analyzers; 3–6 for long-term implantables	Long-term antifouling and biostability; sterilization and packaging; regulatory validation of analytical performance
Functionally graded materials	Orthopedic bone grafts/segmental defects	Modulus matching; load transfer; vascularized ingrowth	Porosity; mineral content; stiffness; factor dose	Volumetric ingrowth; mechanical gradation; drug depots	Batch heterogeneity; gradient drift after sterilization; low throughput	4–6 (preclinical/early clinical)	Sterilization compatibility; endotoxin control; scale-up quality control
	Periodontal/alveolar regeneration scaffolds	Bone-periodontal ligament-cementum regeneration; vascularization	Stiffness/mineral gradients; porosity; factor dose	Multitissue interface formation with volumetric cues	Wet-field handling; fixation/alignment; gradient stability	3–5 (preclinical)	Sterilization stability; surgical workflow fit; combo-product regulation
	Vascular grafts	Through-wall compliance match; patency; transmural infiltration	Stiffness/compliance; porosity; fiber orientation	Compliance match reduces intimal hyperplasia; cell ingrowth	Handling damage; suture retention vs compliance trade-off; creep	4–6 (preclinical/first-in-human)	Long-term patency; sterilization effects on mechanics; heavy bench/animal testing
	Wound dressings	Epidermal–dermal integration; exudate management; vascularization	Porosity; stiffness; hydration; factor dose	Volumetric matrix and transport control; sustained release	Swelling/shape fidelity; infection risk in thick scaffolds; cost	4–6 (preclinical/limited clinical)	Bioburden control; reproducibility
	Osteochondral scaffolds (bone-cartilage)	Continuous modulus/mineral gradient; zonal regeneration	Stiffness; mineral content; pore architecture; factor dose	Load transfer across hard–soft tissues; zonal guidance	Complex fixation; uneven remodeling; manufacturing complexity	4–6 (strong preclinical pipeline)	Long-term multizone integration; multiend point regulatory claims
	Cartilage/intervertebral disc constructs	Depth-wise modulus/charge gradient; zonal chondrocyte guidance	Stiffness; Glycosaminoglycans/sulfation; fiber orientation	Restores depth-dependent mechanics and transport	Long-term durability; integration; nutrient diffusion limits	3–5 (preclinical)	Mechanical fatigue testing; complex manufacturing; *in vivo* monitoring
	Corneal stromal substitutes	Depth-wise refractive/mechanical match; stromal guidance	Stiffness; hydration; collagen alignment	Volumetric replacement with optical/mechanical fidelity	Optical haze; dehydration/rehydration drift; suture handling	3–4 (early preclinical)	Optical clarity over time; immune response; sterilization compatibility

a Legend for Technology Readiness Level: 1–3 concept; 4–6 preclinical; 7–8 clinical prototype/limited use; 9 established clinical use.

## 5 Conclusions and Perspectives

This review focuses on the fabrication and biomedical applications of functionally graded surfaces and materials that feature spatial variations in terms of composition, structure, and other properties. For each method, we discuss the principle of fabrication while highlighting its capability to enable functionalities that are difficult to achieve with conventional homogeneous surfaces or materials. Despite significant progress in recent years, this field still faces a set of challenges. For example, prior to clinical translation, it is essential to ensure the repeatability and reproducibility of the fabrication method, as well as to scale up the production volume without compromising quality control. It is also worth exploring the rational integration of hierarchical structures across multiple scales while leveraging the powers of modeling and artificial intelligence (AI). Here we briefly discuss each of these subjects, aiming to inspire new research efforts in this field.

### 5.1 Repeatability and Reproducibility

Repeatability and reproducibility are vital parameters for assessing whether the fabrication method holds promise for clinical applications.^270^ While closely related, repeatability and reproducibility refer to different aspects of experimental reliability. As shown in Figure 44A, repeatability reflects the consistency of results within a single laboratory, including how reliable the results are when the same investigator performs the experiment multiple times and how robust the results are over time. In contrast, reproducibility refers to the ability to obtain consistent results when an experiment is independently replicated by different teams using the same protocol and instrumentation. In essence, reproducibility ensures that similar results can be reliably obtained across laboratories. It is important to note that repeatability and reproducibility are not exclusive to functionally graded surfaces and materials but are widely encountered across the broader field of materials research.

**Figure 44:**
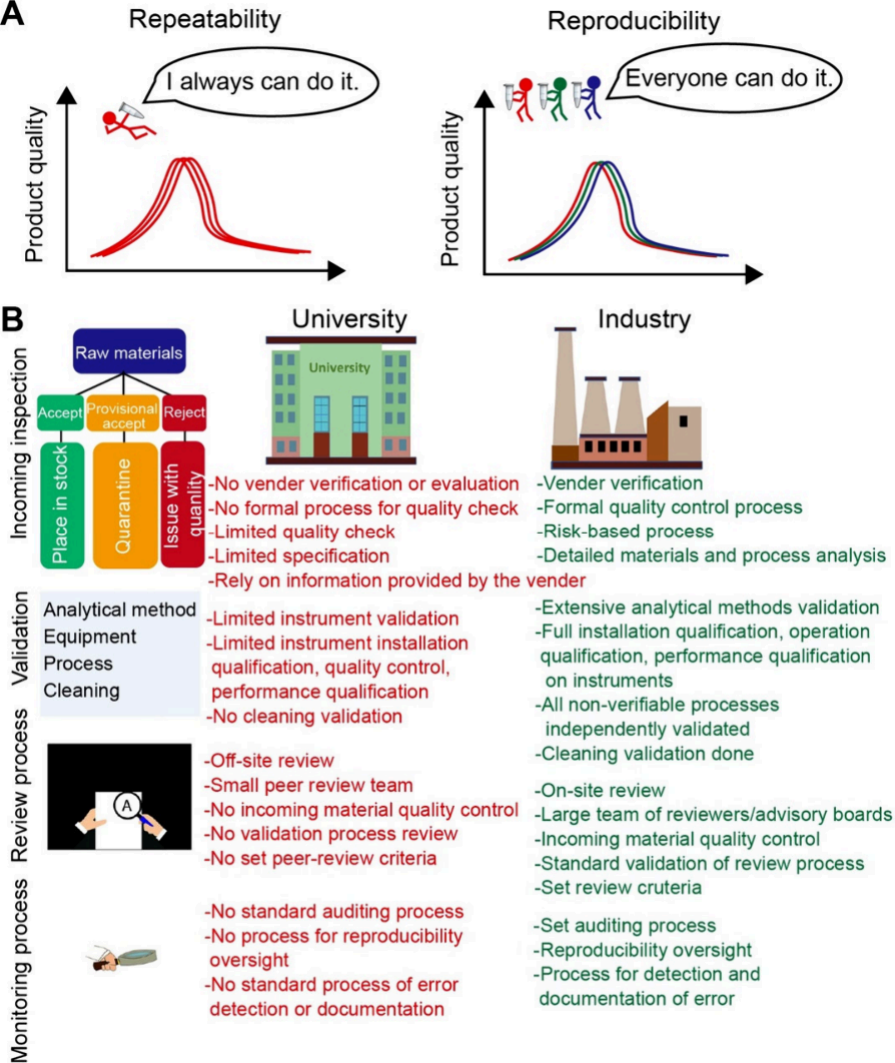
Repeatability and reproducibility issues of a fabrication method. (A) Schematic illustrating the differences between repeatability and reproducibility. (B) Comparison of academic and clinical/industrial practices in terms of repeatability and reproducibility. Text colors indicate the implementation levels: red (poor) and green (full). Reproduced with permission from ref 271. Copyright 2023 American Chemical Society.

Despite recent progress, the lack of repeatability and reproducibility still presents a significant barrier to the translation of functionally graded surfaces and materials.^271^ As shown in Figure 44B, the barrier often arises from several key factors. For instance, lack of rigorous control over raw material sourcing, caused by frequent changes in vendors or unverified certificates of analysis, can directly compromise the consistency of a fabrication method. Moreover, the custom-built laboratory equipment often lacks standardized calibration, validation, and routine maintenance that should be regularly performed in industrial settings. The discrepancies are further amplified by differences in oversight: academic research generally relies on peer review, and it does not include the rigorous process-level audits and quality assurance systems standard in clinical and industrial settings.

Achieving both high repeatability and reproducibility for the fabrication methods requires a two-pronged strategy. First, it is essential to standardize technology and systematically document the fabrication protocols.^272^ The strategy will enable efficient knowledge transfer across different laboratories and facilities. Second, the development of open-access data sets and the promotion of collaborative benchmarking initiatives will be pivotal for establishing reproducible and clinically translatable graded systems. Both steps will help bridge the gap between laboratory innovation and commercial deployment.

### 5.2 Precision and Spatial Resolution

Precision in the fabrication of functionally graded surfaces and materials refers to the ability to define, control, and execute the desired spatial variations of material properties with high accuracy and fine resolution.^273^ It ensures that the actual gradient profile closely matches the intended design while allowing for high spatial resolution in implementing the variations. For instance, if we aim for a 1D gradient in a property that ranges from 0% to 100% across a certain distance, the precision ensures that the fabricated gradient closely matches the desired profile, without any unintended fluctuations or deviations. Such a control is particularly important in specific biomedical contexts, where spatially defined gradients must be tailored to meet specific demands, such as replication of the features of a native tissue interface that depends on the graded transitions in structure and composition.

As we discussed in Sections 2 and 3, essentially all fabrication methods have experienced significant progress in generating gradients with appreciable spatial resolution by modulating experimental parameters such as concentration, light exposure, or deposition rate. However, they still face considerable inherent limitations. For methods such as progressive immersion, they offer a facile and scalable approach to generating continuous gradients that often exhibit low spatial precision and poor reproducibility. The deficiencies are particularly evident near the regions where the substrate moves into or out of the solution. The uncontrolled fluid dynamics in these regions tend to cause edge effects and abrupt discontinuities in the gradient profile. Moreover, the above approaches still struggle to generate multidimensional gradients with nanoscale resolution—an increasingly important requirement for designing biomimetic interfaces.

In contrast, 3D printing has emerged as a promising strategy for achieving greater design flexibility and enhanced spatial control in the fabrication of gradients. By leveraging techniques such as graded extrusion and voxel-by-voxel control, 3D printing allows for the precise and localized deposition of materials with tunable compositions. Such a control enables the fabrication of customized gradients tailored for specific biomedical applications. However, 3D printing still falls short of the precision and stability required for widespread clinical translation or industrial application. The limitations arise not only from hardware constraints or nonoptimized parameters but also from difficulties in accurately delivering raw materials. For example, Li and co-workers reported a substantial discrepancy in composition between premixed feedstock and the deposited material (Figure 45A), and they attributed it to variations in particle size and density, where smaller, lighter particles flowed faster than their larger, heavier counterparts.^274^ Such an inconsistency in particle transport compromises the accuracy of gradient formation, ultimately undermining the precision required for constructing well-defined architectures.

**Figure 45:**
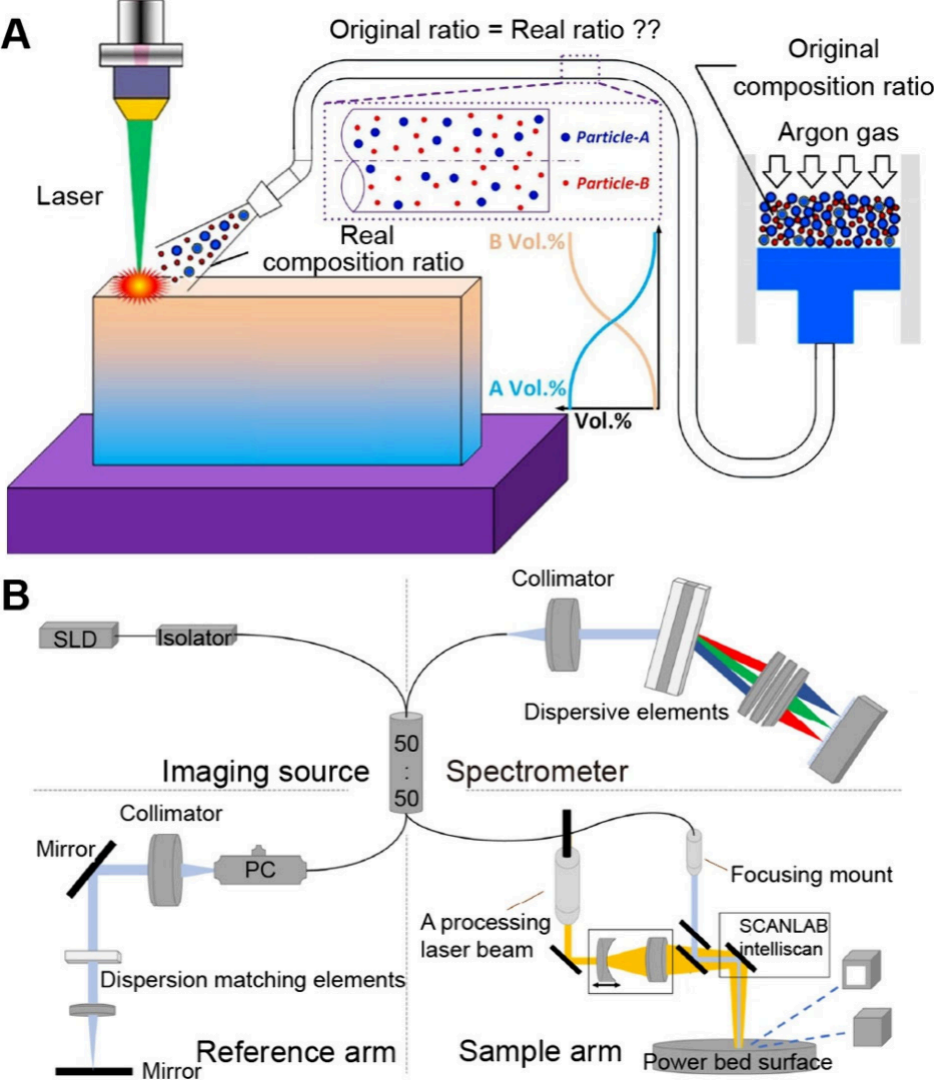
Challenges in and strategies for improving the precision of a fabrication method. (A) Schematic showing the fabrication of graded materials via laser melting deposition. (B) Schematic showing a commercial platform that integrates spectral-domain OCT for *in situ* monitoring. The system consists of a low-coherence superluminescent diode, a high-speed scanning spectrometer, and a fiber-based Michelson interferometer. (A) Reproduced with permission from ref 274. Copyright 2018 Elsevier. (B) Reproduced with permission from ref 275. Copyright 2018 Elsevier.

To achieve a tight control over the gradients, integration of high-precision, *in situ*, and real-time monitoring techniques into the fabrication process has proven essential. Specifically, advanced tools such as laser-induced breakdown spectroscopy, optical coherence tomography (OCT), and infrared thermography provide immediate feedback on critical parameters, including composition, temperature, microstructure, and interface integrity, throughout the fabrication process. The real-time data is crucial for quickly identifying deviations from the target gradient profiles. As shown in Figure 45B, Matthews and co-workers successfully employed large-area spectral-domain OCT to achieve real-time monitoring of surface roughness.^275^ In the future, when integrated with AI-based image recognition, such monitoring systems can facilitate the implementation of closed-loop feedback control, enabling unprecedented precision in the fabrication of gradients. While these advances improve the precision and resolution of fabricated gradients toward maximal controllability, it is important to note that the gradients in nature feature intrinsic variability. For instance, the tendon-to-bone interface varies across individuals in both structural dimension and molecular organization, suggesting the personalized nature of biological systems. Therefore, beyond the pursuit of superior precision and resolution, an emerging direction lies in integrating controlled variability or adaptive features to emulate the stochastic yet functional characteristics of natural gradients.

### 5.3 Scalability for Mass Production

Although significant progress has been made in the laboratory-scale fabrication of graded surfaces and materials, moving these innovations to mass production or widespread clinical applications remains a substantial challenge.^276^ Most of the current fabrication methods operate at relatively slow throughputs, making them unsuitable to meet the speed and volume demands for industrial manufacturing. For example, mask-assisted fabrication requires several steps, including design of masks, preparation of photosensitive substrates, and selective UV exposure. Fabricating a sample often requires an extended period, making it challenging to maintain consistency between batches. While the methods may work well in a research setting, they are impractical for mass production, where thousands or even millions of units are involved.

Beyond throughput limitations, the scale-up from laboratory to mass production faces two major challenges: *i*) whether the fabrication methodology still works at a large scale and *ii*) whether the production can operate safely, continuously, and economically. Specifically, the first challenge is to reproduce the same gradient as in the lab at a large volume. As the system increases in size, the physical environment becomes more difficult to control, particularly in terms of heat transfer, mass transfer, and reagent mixing. In diffusion- or reaction-driven systems, such factors often lead to nonuniform gradients and uncontrolled cross-linking or curing behavior at large scales. When scaling up, even minor fluctuations in temperature, flow rate, or composition can be amplified, compromising precision. The second issue involves economic, safety, and logistical considerations inherent to industrial-scale operations. Large-volume handling of reactive or volatile chemicals necessitates the use of industrial-grade equipment, such as stainless-steel reactors, capable of withstanding high temperatures, pressures, and corrosion. Moreover, a reaction that is technically viable in the lab may be economically impractical at the large scale due to reliance on high-purity, expensive reagents. Thus, the fabrication method must be optimized for low-purity, industrial-grade raw materials that can be reliably and affordably sourced in bulk when developing effective strategies for mass production.

To achieve actual mass production, future efforts should focus on several key aspects. First, developing novel high-throughput fabrication strategies, such as continuous lithography, roll-to-roll patterning, and advanced multimaterial 3D printing, will be instrumental in bridging the gap between precision and productivity.^277–279^ A promising platform is automated, reconfigurable microfluidics (Figure 46), which allows users to digitally design the desired configurations on a computer and implement them on reprogrammable chips with a precise control.^280^ Such systems could dynamically switch between multiple operational states, offering a versatile route to large-scale yet finely tuned gradient fabrication. Second, strategies for cost reduction, such as optimizing raw material sourcing, implementing solvent recycling, and improving energy efficiency, are crucial to ensure economic feasibility at all scales. Finally, establishing standardized protocols and robust quality assurance frameworks is vital for ensuring reproducibility, regulatory compliance, and consistent performance across production batches. Taken together, these advancements will be pivotal for transforming functionally graded materials from niche, lab-scale prototypes into scalable and deployable platforms for commercial applications.

**Figure 46:**
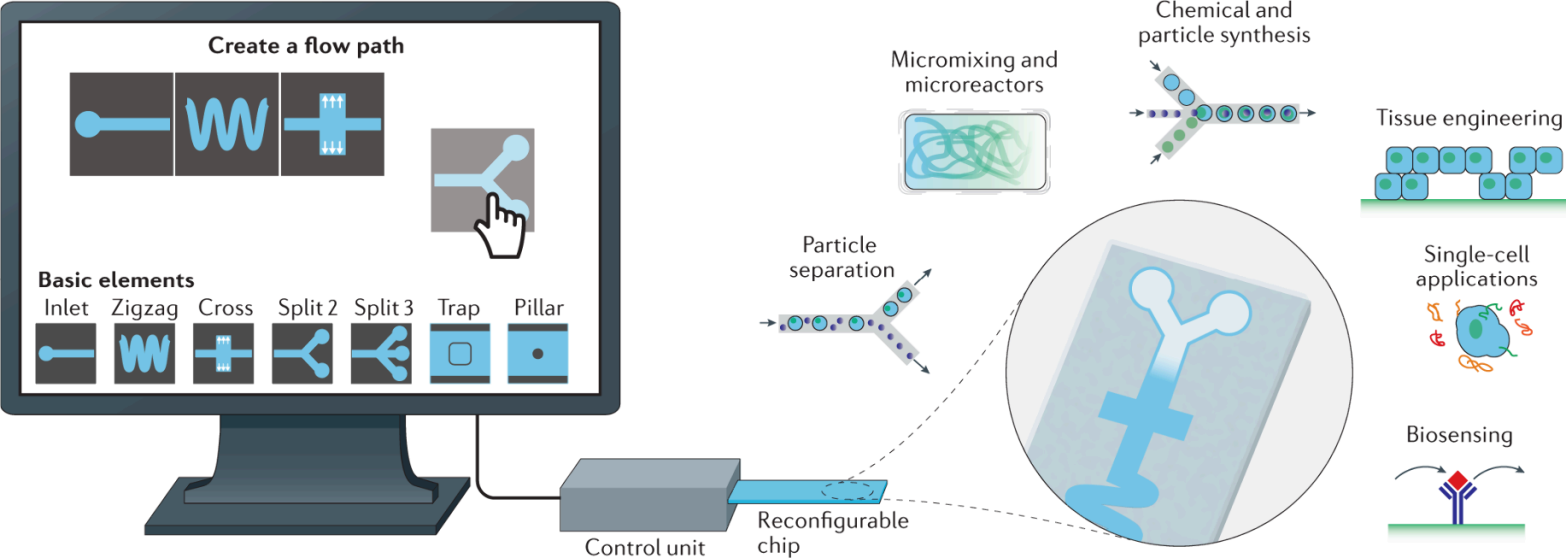
Vision for the reconfigurable microfluidic platform. Reproduced with permission from ref 280. Copyright 2021 Springer Nature.

### 5.4 Hierarchical Structures across Length Scales

In the context of functionally graded materials, hierarchical structures refer to the systematic and multilevel organization of material elements across different length scales ranging from nanoscale to macroscale.^281^ The use of such hierarchical designs is increasingly recognized as essential for replicating the complexity of natural tissues and advancing clinical translation. Nature offers a rich source of inspiration for fabricating structural motifs that seamlessly integrate gradients at different length scales through a bottom-up approach (Figure 47A).^282^ Representative hierarchical patterns include lamellar, columnar, coaxial, Bouligand, and array-based structures (Figure 47B).^283^ Each type of structure offers distinct advantages in terms of mechanical reinforcement or directional transportation. For instance, lamellar structures can enhance the strength and fracture resistance of ceramic-like tissues. In comparison, coaxial arrangements found in wood and bone provide both mechanical stiffness and efficient mass transport. An illustrative example is the Bouligand structure found in the claw of the American lobster (Figure 47C), which exhibits a continuous transition in mechanical and structural properties from nanoscale fibril orientation to macroscale geometry.^283^ By following the designs in nature, hierarchical structures have been fabricated with gradients for guiding cell migration, promoting osteochondral interface regeneration, or enhancing vascular integration. Admittedly, the manmade hierarchical structures often fail to replicate the intricate detail and multifunctionality typically found in nature.

**Figure 47:**
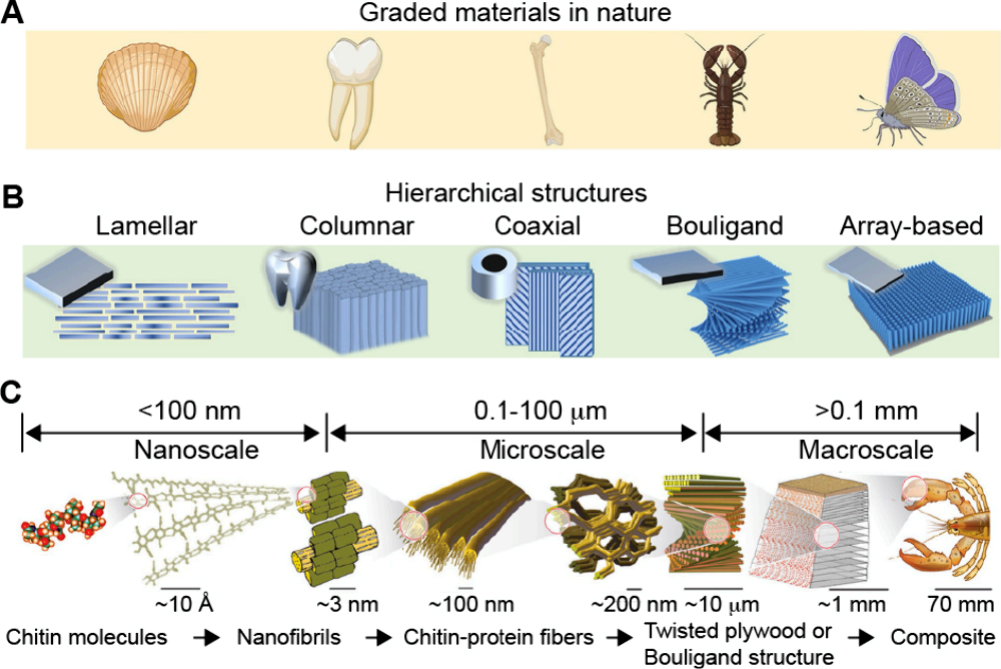
Representative hierarchical structures in nature and their classification. (A) Examples of natural materials that exhibit graded architectures across multiple length scales. (B) Major structural motifs observed in biological hierarchical systems. (C) Hierarchical structures of the lobster claw. Reproduced with permission from ref 283. Copyright 2023 The Author(s) (CC BY 4.0).

Regarding bioinspired hierarchical structures, it is essential to address two fundamental questions: where do they come from and which direction will they take? In other words, we need to learn from nature and thoroughly understand the chemical or structural determinants that contribute to hierarchical organization. Moreover, to guide their development, it is crucial to uncover the relationships among composition, structure, and properties, and how these parameters dictate function, thereby enabling the derivation of transferable design principles. Taking the royal water lily inspired patch for wound healing as an example (Figure 41), we need to understand how the hierarchical structures are designed and what specific benefits the structures offer for wound repair. Such insights lay the groundwork for the rational design of next-generation surfaces and materials featuring gradations. Moreover, multiscale computational modeling and AI-assisted optimization can provide powerful tools for tailoring hierarchical architectures that meet the criteria of biological relevance, mechanical robustness, and scalable manufacturability.^284^ Future efforts should also explore dynamic, self-adaptive hierarchical materials capable of remodeling in response to environmental or biological cues, further narrowing the gap between engineered and natural systems.

### 5.5 Modeling and AI-Aided Fabrication

Alongside advances in fabrication precision, modeling and AI-assisted fabrication have become essential in developing functionally graded surfaces and materials. The computational algorithms provide a deeper insight into how complex geometries and material formulations perform under varying conditions, enabling rational design, predictive optimization, and real-time process control.^285^

Modeling, particularly physics-based methods such as finite element analysis, is vital in simulating how processing parameters influence stress distribution, heat transfer, or structural deformation across graded regions.^286,287^ In one report, Studart and co-workers employed multimaterial 3D printing to fabricate a biomimetic intervertebral disc (IVD) with a tailored gradient in stiffness (Figure 48A).^287^ By leveraging finite element modeling (Figure 48B), they identified local density of strain energy as a key parameter in governing failure localization. The study not only demonstrates the functional advantages of stiffness gradient in load-bearing but also provides a quantitative design guideline for optimizing mechanical performance. Moreover, the consistency between simulated and experimental results further validates the essential role of modeling in accelerating the design and qualification of functionally graded surfaces and materials.

**Figure 48:**
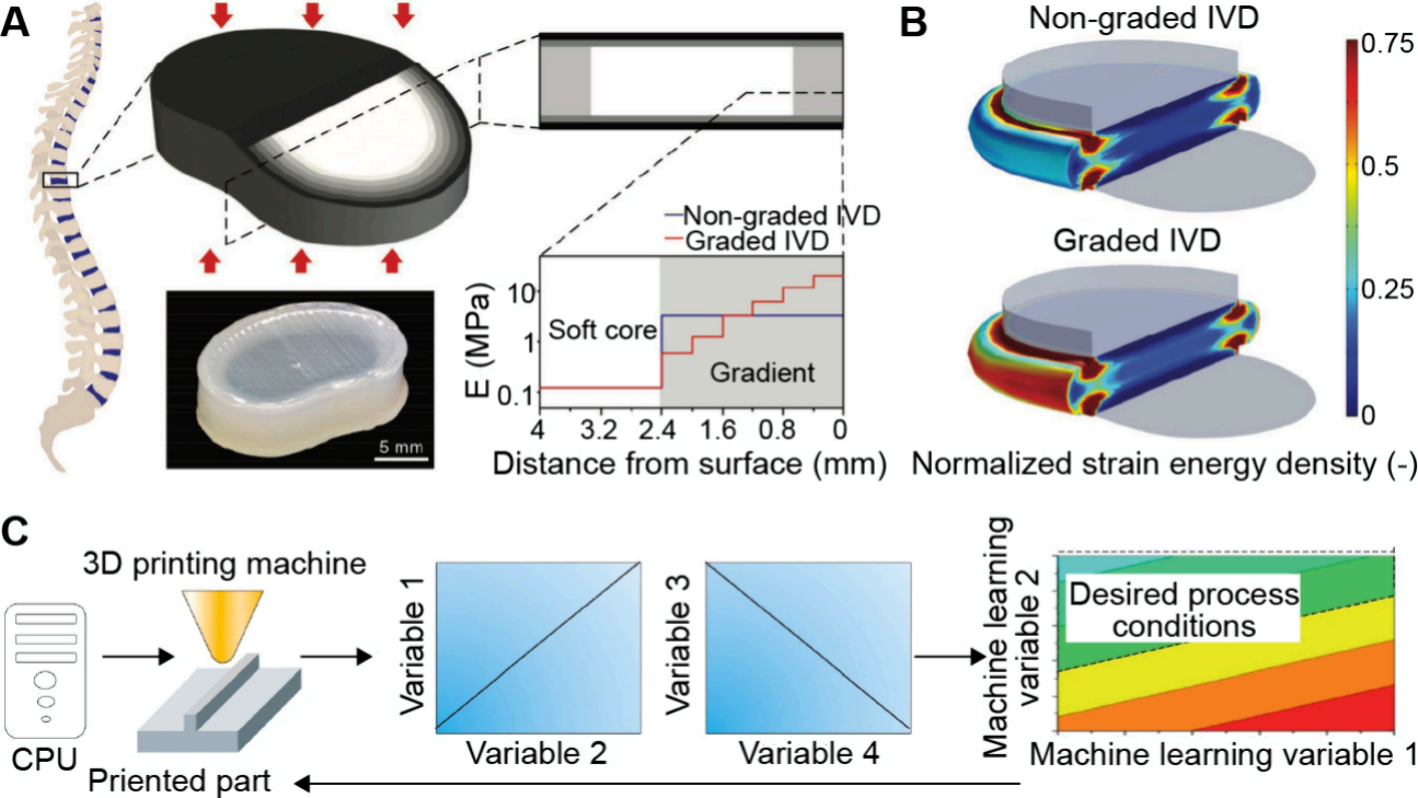
Modeling and AI-aided fabrication of graded materials. (A) Schematic showing the mechanical loading conditions and the graded design in stiffness of an artificial IVD. (B) Finite element analysis of both homogeneous and graded IVD constructs, showing distinct distributions of strain energy density, either concentrated at the attachment edge or along the surface. The shaded region represents the original undeformed configuration. (C) Schematic showing the optimization of process parameters to obtain the desired attribute of a part using machine learning. (A, B) Reproduced with permission from ref 287. Copyright 2018 The Author(s) (CC-BY-NC-ND 4.0). (C) Reproduced with permission from ref 284. Copyright 2020 Springer Nature.

In recent years, AI has emerged as a powerful complement to conventional modeling in materials research. Unlike physics-based methods, AI-driven techniques, particularly those utilizing machine learning, leverage extensive data sets to forecast outcomes, identify defects, and optimize the design and fabrication of graded surfaces and materials.^288^ By enabling rapid and reliable adjustments of parameters (Figure 48C), machine learning facilitates the attainment of desired material properties with greater efficiency.^284^ Furthermore, the integration of standardized AI frameworks reduces reliance on trial-and-error experimentation, minimizes production variability, and improves the overall consistency of complex, graded architectures. As AI technologies continue to evolve, their synergy with conventional modeling is poised to unlock unprecedented levels of precision, scalability, and functionality in the development of functionally graded surfaces and materials.

In conclusion, functionally graded surfaces and materials present a transformative paradigm in biomedical engineering, offering unparalleled opportunities to bridge the gap between synthetic constructs and biological systems. As our understanding of the design principles are deepened, encompassing reproducibility, precision, scalability, hierarchical integration, and computational strategies, we are poised to overcome existing barriers to clinical translation. By advancing from laboratory-scale innovations to standardized, high-throughput fabrication, the tailored material systems will unlock new frontiers in biomedical engineering. We envision this review not only as a comprehensive overview of current advances but also as a strategic roadmap that inspires researchers to integrate multiscale gradients with bioinspired thinking and emerging fabrication technologies. Ultimately, the convergence of materials innovation, advanced manufacturing, and AI-driven design will elevate graded systems from experimental models to transformative clinical solutions, fulfilling their promise as next-generation tools in biomedicine and beyond.

## Abbreviations

1D: one-dimensional
2D: two-dimensional
3D: three-dimensional
AFM: atomic force microscopy
AI: artificial intelligence
algMC: alginate-methylcellulose
ALP: alkaline phosphatase
ASCs: adipose-derived stem cells
ASTM: American Society for Testing and Materials
BC: bacterial cellulose
BMP: bone morphogenetic protein
BSA: bovine serum albumin
Col-10: collagen type X
CPC: calcium phosphate cement
CS: chitosan
DLP: digital light processing
DRG: dorsal root ganglion
DS: diclofenac sodium
ECM: extracellular matrix
EDX: energy-dispersive X-ray spectroscopy
EGF: epidermal growth factor
FGF: fibroblast growth factor
FITC-BSA: fluorescein isothiocyanate-labeled BSA
GFP: green fluorescence protein
G-NGF+OS: graded NGF along an oriented microstructure
H_2_O_2_: hydrogen peroxide
HA: hyaluronic acid
HAp: hydroxyapatite
HAV: His-Ala-Val
HhAg: Hedgehog agonist
HRP: horseradish peroxidase
HUVECs: human umbilical vein endothelial cells
IVD: intervertebral disc
LN_2_: liquid nitrogen
MagHA: superparamagnetic hydroxyapatite
MC: methylcellulose
micro-CT: microcomputed tomography
MMP-9: matrix metalloproteinase-9
MPI: mean pixel intensity
MSCs: mesenchymal stem cells
NGF: nerve growth factor
NIR: near-infrared
Nor: norbornene group
OCN: osteocalcin
OCT: optical coherence tomography
OPN: osteopontin
OS: oriented microstructure
PA: polyacrylamide
PAA: polyacrylamide-acrylamide
PCL: polycaprolactone
PCM: phase-change material
PDMS: poly­(dimethylsiloxane)
PEG: poly­(ethylene glycol)
PLGA: poly­(lactic-*co*-glycolic acid)
PNIPAM: poly­(*N*-isopropylacrylamide)
PS: polystyrene
Pv: gas pressure
PVA: poly­(vinyl alcohol)
RFP: red fluorescence protein
RGD: Arg-Gly-Asp
RMS: root-mean-square
RS: randomly structured
s-Cu-gelation: Cu^2+^-based thiolate gelatin hydrogel
SCXA: scleraxis A
SDF-1α: stromal cell-derived factor-1α
SEM: scanning electron microscopy
SF/Col: silk fibroin/collagen
SPION: superparamagnetic iron oxide nanoparticle
s-Zn-gelation: Zn^2+^-based thiolate gelatin hydrogel
Tg: glass transition temperature
TGF: transforming growth factor
U-NGF+OS: uniformly NGF distribution along an oriented microstructure
UV: ultraviolet
VEGF: vascular endothelial growth factor
vFF: valve-based flow-focusing

## References

[1] Zhu C., Qiu J., Pongkitwitoon S., Thomopoulos S., Xia Y. (2018). Inverse Opal Scaffolds with Gradations
in Mineral Content for Spatial
Control of Osteogenesis. Adv. Mater..

[2] Behravesh E., Zygourakis K., Mikos A. G. (2003). Adhesion and Migration of Marrow-Derived
Osteoblasts on Injectable *In Situ* Crosslinkable Poly
(Propylene Fumarate-*co*-Ethylene Glycol)-Based Hydrogels
with a Covalently Linked RGDS Peptide. J. Biomed.
Mater. Res., Part A.

[3] Chen Y., Kinoshita S., Yan E., Hao M., Shen H., Gelberman R., Thomopoulos S., Xia Y. (2024). A Novel Bi-Directional
and Bi-Temporal Delivery System for Enhancing Intrasynovial Tendon
Repair. Materials and Interfaces.

[4] Han S., Xu H., Chen F., Wang G. (2023). Construction Relationship Between
a Functionally Graded Structure of Bamboo and Its Strength and Toughness:
Underlying Mechanisms. Constr. Build. Mater..

[5] Amada S. (1995). Hierarchical
Functionally Gradient Structures of Bamboo, Barley, and Corn. MRS Bull..

[6] Ju J., Bai H., Zheng Y., Zhao T., Fang R., Jiang L. (2012). A Multi-Structural
and Multi-Functional Integrated Fog Collection System in Cactus. Nat. Commun..

[7] Pagani S., Liverani E., Giavaresi G., De Luca A., Belvedere C., Fortunato A., Leardini A., Fini M., Tomesani L., Caravaggi P. (2021). Mechanical
and *In Vitro* Biological
Properties of Uniform and Graded Cobalt-Chrome Lattice Structures
in Orthopedic Implants. J. Biomed. Mater. Res.,
Part B.

[8] Miyamoto, Y. ; Kaysser, W. ; Rabin, B. ; Kawasaki, A. ; Ford, R. G. Functionally Graded Materials: Design, Processing and Applications; Springer Science & Business Media, 2013.

[9] Leong K. F., Chua C. K., Sudarmadji N., Yeong W. Y. (2008). Engineering Functionally
Graded Tissue Engineering Scaffolds. J. Mech.
Behav. Biomed. Mater..

[10] Sagner A., Briscoe J. (2017). Morphogen Interpretation: Concentration,
Time, Competence,
and Signaling Dynamics. Wiley Interdiscip. Rev.:Dev.
Biol..

[11] Bier E., De Robertis E. M. (2015). BMP Gradients:
A Paradigm for Morphogen-Mediated Developmental
Patterning. Science.

[12] Song J., Li L., Fang L., Zhang E., Zhang Y., Zhang Z., Vangari P., Huang Y., Tian F., Zhao Y., Chen W., Xue J. (2023). Advanced Strategies of Scaffolds
Design for Bone Regeneration. BMEMat.

[13] Bel-Vialar S., Itasaki N., Krumlauf R. (2002). Initiating
Hox Gene Expression: In
the Early Chick Neural Tube Differential Sensitivity to FGF and RA
Signaling Subdivides the *HoxB* Genes in Two Distinct
Groups. Development.

[14] Studer M., Lumsden A., Ariza-McNaughton L., Bradley A., Krumlauf R. (1996). Altered Segmental
Identity and Abnormal Migration of Motor Neurons in Mice Lacking *Hoxb*-1. Nature.

[15] Dasen J. S., Liu J.-P., Jessell T. M. (2003). Motor Neuron
Columnar Fate Imposed
by Sequential Phases of Hox-C Activity. Nature.

[16] Fukuda K. (2020). Corneal Fibroblasts:
Function and Markers. Exp. Eye Res..

[17] Lovicu F., McAvoy J., De Iongh R. (2011). Understanding
the Role of Growth
Factors in Embryonic Development: Insights from the Lens. Philos. Trans. R. Soc., B.

[18] Lovicu F., McAvoy J. (2005). Growth Factor Regulation
of Lens Development. Dev. Biol..

[19] Pierscionek B. K., Regini J. W. (2012). The Gradient Index
Lens of the Eye: An Opto-Biological
Synchrony. Prog. Retinal Eye Res..

[20] Zhao H., Brown P. H., Magone M. T., Schuck P. (2011). The Molecular Refractive
Function of Lens γ-Crystallins. J. Mol.
Biol..

[21] Mao A., Chen J., Bu X., Tian L., Gao W., Saiz E., Bai H. (2023). Bamboo-Inspired
Structurally Efficient
Materials with a Large Continuous Gradient. Small.

[22] Alexandre, N. ; Martinho Lopes, A. ; Guerra Guimarães, T. ; Lopes, B. ; Sousa, P. ; Sousa, A. C. ; Damásio Alvites, R. ; Colette Maurício, A. Biomechanical Basis of Bone Fracture and Fracture Osteosynthesis in Small Animals; IntechOpen Limited: London, U.K., 2023.

[23] Hambli, R. ; Hattab, N. Application of Neural Network and Finite Element Method for Multiscale Prediction of Bone Fatigue Crack Growth in Cancellous Bone. In Multiscale Computer Modeling in Biomechanics and Biomedical Engineering; Springer, 2012; pp 3–30.

[24] Mizuno K., Hirashima T., Toda S. (2024). Robust Tissue Pattern Formation by
Coupling Morphogen Signal and Cell Adhesion. EMBO Rep..

[25] Sugiyama Y., Reed D. A., Herrmann D., Lovicu F. J., Robinson M. L., Timpson P., Masai I. (2024). Fibroblast Growth Factor-Induced
Lens Fiber Cell Elongation Is Driven by the Stepwise Activity of Rho
and Rac. Development.

[26] Woo, S. L. ; An, K. ; Frank, C. B. ; Livesay, G. A. ; Ma, C. B. ; Zeminski, J. A. ; Wayne, J. S. ; Myers, B. S. Anatomy, Biology, and Biomechanics of Tendon and Ligament. In Orthopaedic Basic Science; American Academy of Orthopaedic Surgeons, 2000; pp 582–616.

[27] Bostrom M. P. G., Boskey A., Kauffman J. K., Einhorn T. A. (2000). Form and Function
of Bone. Orthopaedic Basic Science.

[28] Dang G., Qin W., Wan Q., Gu J., Wang K., Mu Z., Gao B., Jiao K., Tay F. R., Niu L. (2023). Regulation and Reconstruction
of Cell Phenotype Gradients Along the Tendon-Bone Interface. Adv. Funct. Mater..

[29] Smith L., Xia Y., Galatz L. M., Genin G. M., Thomopoulos S. (2012). Tissue-Engineering
Strategies for the Tendon/Ligament-to-Bone Insertion. Connect. Tissue Res..

[30] Hu Y., Birman V., Deymier-Black A., Schwartz A. G., Thomopoulos S., Genin G. M. (2015). Stochastic Interdigitation as a Toughening Mechanism
at the Interface Between Tendon and Bone. Biophys.
J..

[31] Thomopoulos S., Genin G. M., Galatz L. M. (2010). The Development and Morphogenesis
of the Tendon-to-Bone Insertion: What Development Can Teach Us About
Healing. J. Musculoskeletal Neuronal Interact..

[32] Apostolakos J., Durant T. J., Dwyer C. R., Russell R. P., Weinreb J. H., Alaee F., Beitzel K., McCarthy M. B., Cote M. P., Mazzocca A. D. (2014). The Enthesis: A Review of the Tendon-to-Bone Insertion. Muscles Ligaments Tendons J..

[33] Chen Y., Hao M., Bousso I., Thomopoulos S., Xia Y. (2024). Reliable Fabrication
of Mineral-Graded Scaffolds by Spin-Coating and Laser Machining for
Use in Tendon-to-Bone Insertion Repair. Adv.
Healthcare Mater..

[34] Zhu C., Qiu J., Thomopoulos S., Xia Y. (2021). Augmenting Tendon-to-Bone Repair
with Functionally Graded Scaffolds. Adv. Healthcare
Mater..

[35] Li X., Xie J., Lipner J., Yuan X., Thomopoulos S., Xia Y. (2009). Nanofiber Scaffolds
with Gradations in Mineral Content for Mimicking
the Tendon-to-Bone Insertion Site. Nano Lett..

[36] Kloxin A. M., Benton J. A., Anseth K. S. (2010). *In Situ* Elasticity
Modulation with Dynamic Substrates to Direct Cell Phenotype. Biomaterials.

[37] Xue J., Wu T., Qiu J., Rutledge S., Tanes M. L., Xia Y. (2020). Promoting
Cell Migration and Neurite Extension along Uniaxially Aligned Nanofibers
with Biomacromolecular Particles in a Density Gradient. Adv. Funct. Mater..

[38] Liu W., Zhang Y., Thomopoulos S., Xia Y. (2013). Generation of Controllable
Gradients in Cell Density. Angew. Chem., Int.
Ed..

[39] Liu W., Lipner J., Xie J., Manning C. N., Thomopoulos S., Xia Y. (2014). Nanofiber Scaffolds
with Gradients in Mineral Content for Spatial
Control of Osteogenesis. ACS Appl. Mater. Interfaces.

[40] Krämer S., Xie H., Gaff J., Williamson J. R., Tkachenko A. G., Nouri N., Feldheim D. A., Feldheim D. L. (2004). Preparation of Protein
Gradients Through the Controlled Deposition of Protein-Nanoparticle
Conjugates onto Functionalized Surfaces. J.
Am. Chem. Soc..

[41] Uedayukoshi T., Matsuda T. (1995). Cellular-Responses on a Wettability Gradient Surface
with Continuous Variations in Surface Compositions of Carbonate and
Hydroxyl-Groups. Langmuir.

[42] Aden B., Street D. P., Hopkins B. W., Lokitz B. S., Kilbey S. M. (2018). Tailoring
Surface Properties through *In Situ* Functionality
Gradients in Reactively Modified Poly­(2-vinyl-4,4-dimethyl azlactone)
Thin Films. Langmuir.

[43] Tanes M. L., Xue J., Xia Y. (2017). A General
Strategy for Generating Gradients of Bioactive
Proteins on Electrospun Nanofiber Mats by Masking with Bovine Serum
Albumin. J. Mater. Chem. B.

[44] Li H., Wu T., Xue J., Ke Q., Xia Y. (2020). Transforming Nanofiber
Mats into Hierarchical Scaffolds with Graded Changes in Porosity and/or
Nanofiber Alignment. Macromol. Rapid Commun..

[45] Wu T., Li X., Xue J., Xia Y. (2024). Rational Fabrication of Functionally-Graded
Surfaces for Biological and Biomedical Applications. Acc. Mater. Res..

[46] Wu T., Xue J., Li H., Zhu C., Mo X., Xia Y. (2018). General Method
for Generating Circular Gradients of Active Proteins on Nanofiber
Scaffolds Sought for Wound Closure and Related Applications. ACS Appl. Mater. Interfaces.

[47] Lai W., Di L., Zhao C., Tian Y., Duan Y., Pan Y., Ye D., Jiang L., Guo Y., He G., Deng W., Guan Y., Huang Y. (2024). Electrospray Deposition for Electronic
Thin Films on 3D Freeform Surfaces: From Mechanisms to Applications. Adv. Mater. Technol..

[48] Wallen H. (2023). Mass Spectrometry
Using Electrospray Ionization. Nat. Rev. Methods
Primers.

[49] Li X., MacEwan M. R., Xie J., Siewe D., Yuan X., Xia Y. (2010). Fabrication of Density
Gradients of Biodegradable Polymer Microparticles
and Their Use in Guiding Neurite Outgrowth. Adv. Funct. Mater..

[50] Xue J., Wu T., Qiu J., Xia Y. (2022). Accelerating Cell Migration along
Radially Aligned Nanofibers through the Addition of Electrosprayed
Nanoparticles in a Radial Density Gradient. Part. Part. Syst. Charact..

[51] Li X., Liang H., Sun J., Zhuang Y., Xu B., Dai J. (2015). Electrospun Collagen
Fibers with Spatial Patterning of SDF1α
for the Guidance of Neural Stem Cells. Adv.
Healthcare Mater..

[52] Fei G., Parra-Cabrera C., Zhong K., Clays K., Ameloot R. (2024). From Grayscale
Photopolymerization 3D Printing to Functionally Graded Materials. Adv. Funct. Mater..

[53] Wong J. Y., Velasco A., Rajagopalan P., Pham Q. (2003). Directed Movement of
Vascular Smooth Muscle Cells on Gradient-Compliant Hydrogels. Langmuir.

[54] Sunyer R., Jin A. J., Nossal R., Sackett D. L. (2012). Fabrication of Hydrogels
with Steep Stiffness Gradients for Studying Cell Mechanical Response. PLoS One.

[55] Liu Y., Takafuji M., Ihara H., Zhu M., Yang M., Gu K., Guo W. (2012). Programmable Responsive Shaping Behavior Induced by
Visible Multi-Dimensional Gradients of Magnetic Nanoparticles. Soft Matter.

[56] Schaller V., Kräling U., Rusu C., Petersson K., Wipenmyr J., Krozer A., Wahnström G., Sanz-Velasco A., Enoksson P., Johansson C. (2008). Motion of
Nanometer-Sized Magnetic Particles in a Magnetic Field Gradient. J. Appl. Phys..

[57] Hua D., Xiong R., Braeckmans K., Scheid B., Huang C., Sauvage F., De Smedt S. C. (2021). Concentration
Gradients in Material
Sciences: Methods to Design and Biomedical Applications. Adv. Funct. Mater..

[58] Valmikinathan C. M., Wang J., Smiriglio S., Golwala N. G., Yu X. (2009). Magnetically
Induced Protein Gradients on Electrospun Nanofibers. Comb. Chem. High Throughput Screening.

[59] Polyak B., Fishbein I., Chorny M., Alferiev I., Williams D., Yellen B., Friedman G., Levy R. J. (2008). High Field Gradient
Targeting of Magnetic Nanoparticle-Loaded Endothelial Cells to the
Surfaces of Steel Stents. Proc. Natl. Acad.
Sci. U. S. A..

[60] Li Z., Zhang Y., Ma M., Wang W., Hui H., Tian J., Chen Y. (2024). Targeted Mitigation of Neointimal
Hyperplasia *via* Magnetic Field-Directed Localization
of Superparamagnetic Iron Oxide Nanoparticle-Labeled Endothelial Progenitor
Cells Following Carotid Balloon Catheter Injury in Rats. Biomed. Pharmacother..

[61] Xu Y., Sun K., Huang L., Dai Y., Zhang X., Xia F. (2024). Magneto-Induced
Janus Adhesive-Tough Hydrogels for Wearable Human Motion Sensing and
Enhanced Low-Grade Heat Harvesting. ACS Appl.
Mater. Interfaces.

[62] Zhang L. W., Dai W. L., Gao C. Y., Wei W., Huang R. R., Zhang X., Yu Y. J., Yang X. P., Cai Q. (2023). Multileveled
Hierarchical Hydrogel with Continuous Biophysical and Biochemical
Gradients for Enhanced Repair of Full-Thickness Osteochondral Defect. Adv. Mater..

[63] Tofail S. A., Bauer J. (2016). Electrically Polarized Biomaterials. Adv. Mater..

[64] Chai Z., Childress A., Busnaina A. A. (2022). Directed Assembly of Nanomaterials
for Making Nanoscale Devices and Structures: Mechanisms and Applications. ACS Nano.

[65] Yan W.-C., Xie J., Wang C.-H. (2018). Electrical Field Guided Electrospray Deposition for
Production of Gradient Particle Patterns. ACS
Appl. Mater. Interfaces.

[66] Tycova A., Prikryl J., Kotzianova A., Datinska V., Velebny V., Foret F. (2021). Electrospray: More Than Just an Ionization Source. Electrophoresis.

[67] Shao J., Zheng Y., Newton M. A. A., Li Y., Xin B. (2023). Effect of
Electric Field Induced by Collector Shape on the Electrospun Jet Motion
and Fiber Structure Evolution. Fibers Polym..

[68] Zhang D., Chang J. (2008). Electrospinning of
Three-Dimensional Nanofibrous Tubes with Controllable
Architectures. Nano Lett..

[69] Zhou Z., Liu N., Zhang X., Ning X., Miao Y., Wang Y., Sun J., Wan Q., Leng X., Wu T. (2022). Manipulating Electrostatic
Field to Control the Distribution of Bioactive Proteins or Polymeric
Microparticles on Planar Surfaces for Guiding Cell Migration. Colloids Surf., B.

[70] Bromberg L. E., Ron E. S. (1998). Temperature-Responsive Gels and Thermogelling Polymer
Matrices for Protein and Peptide Delivery. Adv.
Drug Delivery Rev..

[71] Andlinger D. J., Kulozik U. (2023). Protein-Protein Interactions Explain
the Temperature-Dependent
Viscoelastic Changes Occurring in Colloidal Protein Gels. Soft Matter.

[72] Karimi M., Zangabad P. S., Ghasemi A., Amiri M., Bahrami M., Malekzad H., Asl H. G., Mandieh Z., Bozorgomid M., Ghasemi A., Boyuk M. R. R. T., Hamblin M. R. (2016). Temperature-Responsive
Smart Nanocarriers for Delivery of Therapeutic Agents: Applications
and Recent Advances. ACS Appl. Mater. Interfaces.

[73] Zhang J., Xue L., Han Y. (2005). Fabrication
Gradient Surfaces by Changing Polystyrene
Microsphere Topography. Langmuir.

[74] Atencia J., Beebe D. J. (2005). Controlled Microfluidic
Interfaces. Nature.

[75] Battat S., Weitz D. A., Whitesides G. M. (2022). Nonlinear
Phenomena in Microfluidics. Chem. Rev..

[76] Mou L., Jiang X. (2017). Materials for Microfluidic
Immunoassays: A Review. Adv. Healthcare Mater..

[77] Hitzbleck M., Delamarche E. (2013). Reagents in
Microfluidics: An ‘In’ and
‘Out’ Challenge. Chem. Soc. Rev..

[78] Liao Z., Zhang Y., Li Y., Miao Y., Gao S., Lin F., Deng Y., Geng L. (2019). Microfluidic Chip Coupled with Optical
Biosensors for Simultaneous Detection of Multiple Analytes: A Review. Biosens. Bioelectron..

[79] Schulte T. H., Bardell R. L., Weigl B. H. (2002). Microfluidic Technologies
in Clinical
Diagnostics. Clin. Chim. Acta.

[80] Dutta D., Ramachandran A., Leighton D. T. (2006). Effect of Channel Geometry on Solute
Dispersion in Pressure-Driven Microfluidic Systems. Microfluid Nanofluid.

[81] Kumar A. U., Ganesh D. S., Krishna T. V., Sashank B., Satyanarayana T. (2022). Modeling and
Investigation on Mixing Characteristics of T- and Y-Shaped Micromixers
for Microfluidic Devices. Mater. Today: Proc..

[82] Hattori A., Yasuda K. (2012). Evaluation of a Centrifuged
Double Y-Shape Microfluidic
Platform for Simple Continuous Cell Environment Exchange. Int. J. Mol. Sci..

[83] Belousov K. I., Bukatin A. S., Chubinskiy-Nadezhdin V. I., Vasilyeva V. Y., Negulyaev Y. A., Evstrapov A. A., Kukhtevich I. V. (2016). Microfluidic
Device with Y-Shaped Design for Study of Cell Migration in Concentration
Gradient of Chemoattractants. Nauchn Priborostr..

[84] Jeon H., Lee Y., Jin S., Koo S., Lee C. S., Yoo J. Y. (2009). Quantitative
Analysis of Single Bacterial Chemotaxis Using a Linear Concentration
Gradient Microchannel. Biomed. Microdevices.

[85] Mahmood S., Nandagopal S., Sow I., Lin F., Kung S. K. P. (2014). Microfluidic-Based,
Live-Cell Analysis Allows Assessment of NK-Cell Migration in Response
to Crosstalk with Dendritic Cells. Eur. J. Immunol..

[86] Weisgrab G., Ovsianikov A., Costa P. F. (2019). Functional 3D Printing for Microfluidic
Chips. Adv. Mater. Technol..

[87] Ahmed H., Ramesan S., Lee L., Rezk A. R., Yeo L. Y. (2020). On-Chip
Generation of Vortical Flows for Microfluidic Centrifugation. Small.

[88] Rush B. M., Dorfman K. D., Brenner H., Kim S. (2002). Dispersion by Pressure-Driven
Flow in Serpentine Microfluidic Channels. Ind.
Eng. Chem. Res..

[89] Abed W. M., Whalley R. D., Dennis D. J., Poole R. J. (2015). Numerical and Experimental
Investigation of Heat Transfer and Fluid Flow Characteristics in a
Micro-Scale Serpentine Channel. Int. J. Heat
Mass Transfer.

[90] Mistretta M., Gangneux N., Manina G. (2022). Microfluidic Dose-Response Platform
to Track the Dynamics of Drug Response in Single Mycobacterial Cells. Sci. Rep..

[91] Sweet E., Yang B., Chen J., Vickerman R., Lin Y., Long A., Jacobs E., Wu T., Mercier C., Jew R. (2020). 3D Microfluidic Gradient
Generator for Combination
Antimicrobial Susceptibility Testing. Microsyst.
Nanoeng..

[92] Jeon N. L., Dertinger S. K., Chiu D. T., Choi I. S., Stroock A. D., Whitesides G. M. (2000). Generation
of Solution and Surface Gradients Using
Microfluidic Systems. Langmuir.

[93] Zhang X., Gao X., Jiang L., Qin J. (2012). Flexible Generation of Gradient Electrospinning
Nanofibers Using a Microfluidic Assisted Approach. Langmuir.

[94] Samandari M., Rafiee L., Alipanah F., Sanati-Nezhad A., Javanmard S. H. (2021). A Simple, Low Cost and Reusable Microfluidic Gradient
Strategy and Its Application in Modeling Cancer Invasion. Sci. Rep..

[95] Ballantine, D. S., Jr. ; Martin, S. J. ; Ricco, A. J. ; Frye, G. C. ; Wohltjen, H. ; White, R. M. ; Zellers, E. T. Materials Characterization. Acoustic Wave Sensors - Theory, Design, and Physico-Chemical Applications; Academic Press, 1997; pp 150–221.

[96] Fick A. (1995). On Liquid
Diffusion. J. Membr. Sci..

[97] Hadden W. J., Young J. L., Holle A. W., McFetridge M. L., Kim D. Y., Wijesinghe P., Taylor-Weiner H., Wen J. H., Lee A. R., Bieback K., Vo B. N., Sampson D. D., Kennedy B. F., Spatz J. P., Engler A. J., Choi Y. S. (2017). Stem Cell Migration and Mechanotransduction
on Linear
Stiffness Gradient Hydrogels. Proc. Natl. Acad.
Sci. U. S. A..

[98] Liu J., Cao D. P., Zhang L. Q. (2008). Molecular Dynamics Study on Nanoparticle
Diffusion in Polymer Melts: A Test of the Stokes-Einstein Law. J. Phys. Chem. C.

[99] Karatrantos A., Composto R. J., Winey K. I., Clarke N. (2017). Polymer and Spherical
Nanoparticle Diffusion in Nanocomposites. J.
Chem. Phys..

[100] Qiu J., Ahn J., Qin D., Thomopoulos S., Xia Y. (2022). Biomimetic Scaffolds with a Mineral
Gradient and Funnel-Shaped Channels
for Spatially Controllable Osteogenesis. Adv.
Healthcare Mater..

[101] Choi Y. H., Chung K. H., Hong H. B., Lee W. S. (2018). Production
of PDMS Microparticles by Emulsification of Two Phases and Their Potential
Biological Application. Int. J. Polym. Mater..

[102] Tran P. A., Fox K., Tran N. (2017). Novel Hierarchical
Tantalum Oxide-PDMS Hybrid Coating for Medical Implants: One Pot Synthesis,
Characterization and Modulation of Fibroblast Proliferation. J. Colloid Interface Sci..

[103] Pino C. J., Haselton F. R., Chang M. S. (2005). Seeding
of Corneal
Wounds by Epithelial Cell Transfer from Micropatterned PDMS Contact
Lenses. Cell Transplant..

[104] Wang P.-Y., Tsai W.-B., Voelcker N. H. (2012). Screening
of Rat
Mesenchymal Stem Cell Behaviour on Polydimethylsiloxane Stiffness
Gradients. Acta Biomater..

[105] Huang Z., Li S., Zhang J., Pang H., Ivankin A., Wang Y. (2023). Localized Photoactuation
of Polymer
Pens for Nanolithography. Molecules.

[106] Li S., Zhang J., He J., Liu W., Wang Y., Huang Z., Pang H., Chen Y. (2023). Functional
PDMS Elastomers:
Bulk Composites, Surface Engineering, and Precision Fabrication. Adv. Sci..

[107] Konku-Asase Y., Yaya A., Kan-Dapaah K. (2020). Curing Temperature
Effects on the Tensile Properties and Hardness of γ- Fe_2_O_3_ Reinforced PDMS Nanocomposites. Adv. Mater. Sci. Eng..

[108] Liu M., Sun J., Chen Q. (2009). Influences of Heating Temperature
on Mechanical Properties of Polydimethylsiloxane. Sens. Actuators, A.

[109] Johnston I. D., McCluskey D. K., Tan C. K., Tracey M. C. (2014). Mechanical
Characterization of Bulk Sylgard 184 for Microfluidics and Microengineering. J. Micromech. Microeng..

[110] Campeau M.-A., Lortie A., Tremblay P., Béliveau M.-O., Dubé D., Langelier È., Rouleau L. (2017). Effect of Manufacturing
and Experimental Conditions on the Mechanical and Surface Properties
of Silicone Elastomer Scaffolds Used in Endothelial Mechanobiological
Studies. Biomed. Eng..

[111] Wang J., Zhuang S. (2022). Chitosan-Based Materials: Preparation,
Modification and Application. J. Cleaner Prod..

[112] Kim T. H., An D. B., Oh S. H., Kang M. K., Song H. H., Lee J. H. (2015). Creating Stiffness
Gradient Polyvinyl
Alcohol Hydrogel Using a Simple Gradual Freezing-Thawing Method to
Investigate Stem Cell Differentiation Behaviors. Biomaterials.

[113] Oh S. H., An D. B., Kim T. H., Lee J. H. (2016). Wide-Range
Stiffness Gradient PVA/HA Hydrogel to Investigate Stem Cell Differentiation
Behavior. Acta Biomater..

[114] Miraftab M., Saifullah A. N., Çay A. (2015). Physical Stabilisation
of Electrospun Poly­(Vinyl Alcohol) Nanofibres: Comparative Study on
Methanol and Heat-Based Crosslinking. J. Mater.
Sci..

[115] Sau S., Pandit S., Kundu S. (2021). Crosslinked Poly (Vinyl Alcohol):
Structural, Optical and Mechanical Properties. Surf. Interfaces.

[116] Katz M. G., Wydeven T. (1982). Selective Permeability
of PVA Membranes. II. Heat-Treated Membranes. J. Appl. Polym. Sci..

[117] Bonetti L., De Nardo L., Fare S. (2023). Crosslinking Strategies
in Modulating Methylcellulose Hydrogel Properties. Soft Matter.

[118] Morozova S., Coughlin M. L., Early J. T., Ertem S. P., Reineke T. M., Bates F. S., Lodge T. P. (2019). Properties of Chemically
Cross-Linked Methylcellulose Gels. Macromolecules.

[119] Li C., Ouyang L., Pence I. J., Moore A. C., Lin Y., Winter C. W., Armstrong J. P., Stevens M. M. (2019). Buoyancy-Driven
Gradients for Biomaterial Fabrication and Tissue Engineering. Adv. Mater..

[120] Zhang J., Wang X., Zhang Y., Yang J., Han X. (2025). Centrifugal
Force Boosts Self-Assembly of Gradient Lattice Photonic
Crystals. Adv. Photonics Res..

[121] Wang Y., Qin X., Feng Y., Zhang T., Wang X., Li J., Yin P., Yu Y., Liu C. (2025). Dual-Gradient Silk-Based Hydrogel for Spatially Targeted
Delivery
and Osteochondral Regeneration. Adv. Mater..

[122] Zhang Q., Lu H., Yun G., Gong L., Chen Z., Jin S., Du H., Jiang Z., Li W. (2024). A Laminated Gravity-Driven Liquid
Metal-Doped Hydrogel of Unparalleled
Toughness and Conductivity. Adv. Funct. Mater..

[123] Parameswaran V., Shukla A. (2000). Processing and Characterization
of
a Model Functionally Gradient Material. J. Mater.
Sci..

[124] Zhang Q., Wang H., Shi J., Luo H., Yin C., Wan Y. (2024). Hydroxyapatite Gradient Poly (Vinyl Alcohol)/Bacteria
Cellulose Bone Scaffold *via* Buoyancy-Driven Gradient
Method. Fibers Polym..

[125] Cuppoletti, J. Nanocomposites with Unique Properties and Applications in Medicine and Industry; IntechOpen Limited: London, U.K., 2011.

[126] Kang C., Rohatgi P. (1996). Transient Thermal Analysis of Solidification
in a Centrifugal Casting for Composite Materials Containing Particle
Segregation. Metall. Mater. Trans. B.

[127] Watanabe Y., Yamanaka N., Fukui Y. (1998). Control of
Composition
Gradient in a Metal-Ceramic Functionally Graded Material Manufactured
by the Centrifugal Method. Composites, Part
A.

[128] Ogawa T., Watanabe Y., Sato H., Kim I.-S., Fukui Y. (2006). Theoretical Study on Fabrication
of Functionally Graded Material
with Density Gradient by a Centrifugal Solid-Particle Method. Composites, Part A.

[129] Pradeep A., Rameshkumar T. (2021). Review on
Centrifugal Casting of
Functionally Graded Materials. Mater. Today:
Proc..

[130] Marrella A., Aiello M., Quarto R., Scaglione S. (2016). Chemical and
Morphological Gradient Scaffolds to Mimic Hierarchically Complex Tissues:
From Theoretical Modeling to Their Fabrication. Biotechnol. Bioeng..

[131] Spinnrock A., Schupp D., Cölfen H. (2018). Nanoparticle
Gradient Materials by Centrifugation. Small.

[132] Oh S. H., Park I. K., Kim J. M., Lee J. H. (2007). *In Vitro* and *In Vivo* Characteristics of
PCL Scaffolds with Pore Size Gradient Fabricated by a Centrifugation
Method. Biomaterials.

[133] Oh S. H., Kim T. H., Lee J. H. (2011). Creating
Growth
Factor Gradients in Three Dimensional Porous Matrix by Centrifugation
and Surface Immobilization. Biomaterials.

[134] Harvey, D. 12.7: Electrophoresis. Libretexts, 2025.

[135] Xu G., Ding Z., Lu Q., Zhang X., Zhou X., Xiao L., Lu G., Kaplan D. L. (2020). Electric Field-Driven
Building Blocks for Introducing Multiple Gradients to Hydrogels. Protein Cell.

[136] Wang Y., Li L., Ji Y.-E., Wang T., Fu Y., Li X., Li G., Zheng T., Wu L., Han Q. (2023). Silk-Protein-Based Gradient Hydrogels with Multimode
Reprogrammable Shape Changes for Biointegrated Devices. Proc. Natl. Acad. Sci. U.S.A..

[137] Xu P., Tan Y., Wang X., Xu H., Wang D., Yang Y., An W., Xu S. (2020). Multidimensional
Gradient
Hydrogel and Its Application in Sustained Release. Colloid Polym. Sci..

[138] Hu Y., Yang Y., Tian F., Xu P., Du R., Xia X., Xu S. (2021). Fabrication of Stiffness Gradient
Nanocomposite Hydrogels
for Mimicking Cell Microenvironment. Macromol.
Res..

[139] Tan Y., Wang D., Xu H., Yang Y., An W., Yu L., Xiao Z., Xu S. (2018). A Fast, Reversible, and Robust Gradient
Nanocomposite Hydrogel Actuator with Water-Promoted Thermal Response. Macromol. Rapid Commun..

[140] Tan Y., Wang D., Xu H., Yang Y., Wang X.-L., Tian F., Xu P., An W., Zhao X., Xu S. (2018). Rapid Recovery Hydrogel Actuators in Air with Bionic Large-Ranged
Gradient Structure. ACS Appl. Mater. Interfaces.

[141] Mo K., He M., Cao X., Chang C. (2020). Direct Current Electric
Field Induced Gradient Hydrogel Actuators with Rapid Thermo-Responsive
Performance as Soft Manipulators. J. Mater.
Chem. C.

[142] Ren Y., Liu Z., Jin G., Yang M., Shao Y., Li W., Wu Y., Liu L., Yan F. (2021). Electric-Field-Induced
Gradient Ionogels for Highly Sensitive, Broad-Range-Response, and
Freeze/Heat-Resistant Ionic Fingers. Adv. Mater..

[143] Ali A., Zafar H., Zia M., ul Haq I., Phull A. R., Ali J. S., Hussain A. (2016). Synthesis,
Characterization, Applications,
and Challenges of Iron Oxide Nanoparticles. Nanotechnol., Sci. Appl..

[144] Lim J., Lanni C., Evarts E. R., Lanni F., Tilton R. D., Majetich S. A. (2011). Magnetophoresis of Nanoparticles. ACS Nano.

[145] Watarai H., Suwa M., Iiguni Y. (2004). Magnetophoresis
and
Electromagnetophoresis of Microparticles in Liquids. Anal. Bioanal. Chem..

[146] Wang Z., Wang K., Huang H., Cui X., Shi X., Ma X., Li B., Zhang Z., Tang X., Chiang M. Y. M. (2018). Bioinspired Wear-Resistant and Ultradurable Functional
Gradient Coatings. Small.

[147] Wang Z., Shi X., Huang H., Yao C., Xie W., Huang C., Gu P., Ma X., Zhang Z., Chen L.-Q. (2017). Magnetically Actuated Functional Gradient Nanocomposites
for Strong and Ultra-Durable Biomimetic Interfaces/Surfaces. Mater. Horiz..

[148] Le Ferrand H., Bouville F., Niebel T. P., Studart A. R. (2015). Magnetically
Assisted Slip Casting of Bioinspired Heterogeneous Composites. Nat. Mater..

[149] Kamranpour N. O., Miri A. K., James-Bhasin M., Nazhat S. N. (2016). A Gel Aspiration-Ejection
System for the Controlled
Production and Delivery of Injectable Dense Collagen Scaffolds. Biofabrication.

[150] Joshi I. M., Mansouri M., Ahmed A., De Silva D., Simon R. A., Esmaili P., Desa D. E., Elias T. M., Brown E. B., Abhyankar V. V. (2024). Microengineering
3D Collagen Matrices with Tumor-Mimetic Gradients in Fiber Alignment. Adv. Funct. Mater..

[151] Spalazzi J. P., Dagher E., Doty S. B., Guo X. E., Rodeo S. A., Lu H. H. (2008). *In Vivo* Evaluation
of a Multiphased Scaffold Designed for Orthopaedic Interface Tissue
Engineering and Soft Tissue-to-Bone Integration. J. Biomed. Mater. Res., Part A.

[152] Zhu C., Pongkitwitoon S., Qiu J., Thomopoulos S., Xia Y. (2018). Design and Fabrication of a Hierarchically Structured Scaffold for
Tendon-to-Bone Repair. Adv. Mater..

[153] Larsen A.-K. K., Chen M. (2025). Cell Electrospinning:
Electrohydrodynamic
Effects on Cell Viability and Beyond. Materials
and Interfaces.

[154] Shahriar S. M. S., Polavoram N. S., Andrabi S. M., Su Y., Lee D., Tran H. Q., Schindler S. J., Xie J. (2024). Transforming Layered
2D Mats into Multiphasic 3D Nanofiber Scaffolds with Tailored Gradient
Features for Tissue Regeneration. BMEMat.

[155] Yan X., Xu B., Xia C., Xu M., Zeng B., Zhang R., Zhu L., Zhang C. (2023). Dual Drug-Loaded
Core-Shell
Nanofiber Membranes *via* Emulsion Electrospinning
and Their Controllable Sustained Release Property. J. Drug Delivery Sci. Technol..

[156] Guo J., Wang T., Yan Z., Ji D., Li J., Pan H. (2022). Preparation and Evaluation of Dual Drug-Loaded Nanofiber
Membranes
Based on Coaxial Electrostatic Spinning Technology. Int. J. Pharm..

[157] Bottino M. C., Thomas V., Janowski G. M. (2011). A Novel Spatially
Designed and Functionally Graded Electrospun Membrane for Periodontal
Regeneration. Acta Biomater..

[158] Khoo W., Chung S. M., Lim S. C., Low C. Y., Shapiro J. M., Koh C. T. (2019). Fracture Behavior
of Multilayer Fibrous
Scaffolds Featuring Microstructural Gradients. Mater. Des..

[159] Chen S., McCarthy A., John J. V., Su Y., Xie J. (2020). Converting 2D Nanofiber Membranes to 3D Hierarchical
Assemblies with
Structural and Compositional Gradients Regulates Cell Behavior. Adv. Mater..

[160] Chen J., Li X., Cui W., Xie C., Zou J., Zou B. (2010). Fibrous Composites
With Anisotropic Distribution of
Mechanical Properties After Layer-by-Layer Deposition of Aligned Electrospun
Fibers. Adv. Eng. Mater..

[161] Adugna, Y. ; Akessa, A. ; Lemu, H. Overview Study on Challenges of Additive Manufacturing for a Healthcare Application. In IOP Conference Series: Materials Science and Engineering, 2021, 1201, 012041.

[162] Bracaglia L. G., Smith B. T., Watson E., Arumugasaamy N., Mikos A. G., Fisher J. P. (2017). 3D Printing for
the Design and Fabrication
of Polymer-Based Gradient Scaffolds. Acta Biomater..

[163] Cao Z., Yang Y., Chen Y., Feng W., Wang L. (2025). Additive Manufacturing
of Bioinspired Structural-Color Materials. Materials
and Interfaces.

[164] Kilian D., Ahlfeld T., Akkineni A. R., Bernhardt A., Gelinsky M., Lode A. (2020). 3D Bioprinting of Osteochondral Tissue
Substitutes-*In Vitro* Chondrogenesis in Multi-Layered
Mineralized Constructs. Sci. Rep..

[165] Eryildiz M. (2025). Biomimetic Design and Fabrication
of Thermally Induced
Radial Gradient Shape Memory Scaffolds Using Fused Deposition Modeling
(FDM) for Bone Tissue Engineering. Proc. Inst.
Mech. Eng., Part L.

[166] Zhou L. Y., Fu J., He Y. (2020). A Review of 3D Printing
Technologies for Soft Polymer Materials. Adv.
Funct. Mater..

[167] McCoul D., Rosset S., Schlatter S., Shea H. (2017). Inkjet 3D Printing of UV and Thermal Cure Silicone Elastomers for
Dielectric Elastomer Actuators. Smart Mater.
Struct..

[168] Zhan H., Ni H., Yu X., Gholipourmalekabadi M., Wang T., Lin K., Pan J., Yuan C. (2025). 3D Bioprinting
in Oral and Craniomaxillofacial Tissue Regeneration: Progress, Challenges,
and Future Directions. BMEMat.

[169] Pataky K., Braschler T., Negro A., Renaud P., Lutolf M. P., Brugger J. (2012). Microdrop Printing of Hydrogel Bioinks
into 3D Tissue-Like Geometries. Adv. Mater..

[170] Melchels F. P., Feijen J., Grijpma D. W. (2010). A Review
on Stereolithography
and Its Applications in Biomedical Engineering. Biomaterials.

[171] Ge Q., Sakhaei A. H., Lee H., Dunn C. K., Fang N. X., Dunn M. L. (2016). Multimaterial 4D Printing with Tailorable Shape Memory
Polymers. Sci. Rep..

[172] Wang M., Li W., Mille L. S., Ching T., Luo Z., Tang G., Garciamendez C. E., Lesha A., Hashimoto M., Zhang Y. S. (2022). Digital Light Processing Based Bioprinting with Composable
Gradients. Adv. Mater..

[173] Kuang X., Wu J., Chen K., Zhao Z., Ding Z., Hu F., Fang D., Qi H. J. (2019). Grayscale
Digital Light Processing 3D Printing for Highly Functionally Graded
Materials. Sci. Adv..

[174] Ullah A., Shah M., Ali Z., Asami K., Ur Rehman A., Emmelmann C. (2025). Additive Manufacturing
of Ceramics *via* the Laser Powder Bed Fusion Process. Int. J. Appl. Ceram. Technol..

[175] Zhang X., Chueh Y.-h., Wei C., Sun Z., Yan J., Li L. (2020). Additive Manufacturing of Three-Dimensional Metal-Glass
Functionally Gradient Material Components by Laser Powder Bed Fusion
with *In Situ* Powder Mixing. Addit. Manuf..

[176] Wei C., Sun Z., Chen Q., Liu Z., Li L. (2019). Additive Manufacturing
of Horizontal and 3D Functionally Graded 316L/Cu10Sn Components *via* Multiple Material Selective Laser Melting. J. Manuf. Sci. Eng..

[177] Li Y., Jahr H., Pavanram P., Bobbert F., Puggi U., Zhang X.-Y., Pouran B., Leeflang M., Weinans H., Zhou J. (2019). Additively
Manufactured Functionally Graded Biodegradable
Porous Iron. Acta Biomater..

[178] Popovich V., Borisov E., Popovich A., Sufiiarov V. S., Masaylo D., Alzina L. (2017). Functionally Graded
Inconel 718 Processed
by Additive Manufacturing: Crystallographic Texture, Anisotropy of
Microstructure and Mechanical Properties. Mater.
Des..

[179] Al-Saedi D. S., Masood S., Faizan-Ur-Rab M., Alomarah A., Ponnusamy P. (2018). Mechanical
Properties and Energy
Absorption Capability of Functionally Graded F2BCC Lattice Fabricated
by SLM. Mater. Des..

[180] Tumbleston J. R., Shirvanyants D., Ermoshkin N., Janusziewicz R., Johnson A. R., Kelly D., Chen K., Pinschmidt R., Rolland J. P., Ermoshkin A. (2015). Continuous Liquid Interface Production of 3D Objects. Science.

[181] Walker D. A., Hedrick J. L., Mirkin C. A. (2019). Rapid, Large-Volume,
Thermally Controlled 3D Printing Using a Mobile Liquid Interface. Science.

[182] Ottino J. M. (1990). Mixing, Chaotic Advection, and Turbulence. Annu. Rev. Fluid Mech..

[183] Hong H., Yeom E. (2022). Numerical and Experimental
Analysis
of Effective Passive Mixing *via* a 3D Serpentine Channel. Chem. Eng. Sci..

[184] Feng Y., Wang J., Zhang H., Wang J., Yang Y. (2021). A 3D-Printed
Continuous Flow Platform for the Synthesis of Methylaluminoxane. Green Chem..

[185] Abate A. R., Romanowsky M. B., Agresti J. J., Weitz D. A. (2009). Valve-Based
Flow Focusing for Drop Formation. Appl. Phys.
Lett..

[186] Costantini M., Jaroszewicz J., Kozon L., Szlazak K., Swieszkowski W., Garstecki P., Stubenrauch C., Barbetta A., Guzowski J. (2019). 3D-Printing
of Functionally Graded
Porous Materials Using On-Demand Reconfigurable Microfluidics. Angew. Chem., Int. Ed..

[187] Idaszek J., Costantini M., Karlsen T. A., Jaroszewicz J., Colosi C., Testa S., Fornetti E., Bernardini S., Seta M., Kasarełło K. (2019). 3D Bioprinting
of Hydrogel Constructs with Cell and Material Gradients for the Regeneration
of Full-Thickness Chondral Defect Using a Microfluidic Printing Head. Biofabrication.

[188] Jain S., Cachoux V. M., Narayana G. H., de Beco S., D’alessandro J., Cellerin V., Chen T., Heuzé M. L., Marcq P., Mège R.-M. (2020). The Role of Single-Cell
Mechanical Behaviour and Polarity in Driving Collective Cell Migration. Nat. Phys..

[189] Stroka K. M., Jiang H., Chen S. H., Tong Z., Wirtz D., Sun S. X., Konstantopoulos K. (2014). Water Permeation
Drives Tumor Cell Migration in Confined Microenvironments. Cell.

[190] Datta N. (2025). Actin Polymerization:
Mechanistic Insights into Cellular Adaptation
Processes. Biol. Bull. Rev..

[191] Gallant N. D., Michael K. E., García A. J. (2005). Cell Adhesion
Strengthening: Contributions of Adhesive Area, Integrin Binding, and
Focal Adhesion Assembly. Mol. Biol. Cell.

[192] Yamada K. M., Sixt M. (2019). Mechanisms of 3D Cell
Migration. Nat. Rev. Mol. Cell Biol..

[193] Van Helvert S., Storm C., Friedl P. (2018). Mechanoreciprocity
in Cell Migration. Nat. Cell Biol..

[194] Kuo C. H. R., Xian J., Brenton J. D., Franze K., Sivaniah E. (2012). Complex Stiffness Gradient Substrates
for Studying
Mechanotactic Cell Migration. Adv. Mater..

[195] Yin X., Zhu X., Wang Z. (2021). Cell Migration
Regulated by Spatially
Controlled Stiffness Inside Composition-Tunable Three-Dimensional
Dextran Hydrogels. Adv. Mater. Interfaces.

[196] Wang Y., Zhang J., Shu X., Wu F., He J. (2024). Controlled Domain Gels with a Wide Stiffness Gradient
Simultaneously
Promote Bone Regeneration and Suppress Tumor Recurrence Through DAPK
Activity. Chem. Eng. J..

[197] Rosalem G. S., Las Casas E. B., Lima T. P., González-Torres L. A. (2020). A Mechanobiological
Model to Study Upstream Cell Migration Guided by Tensotaxis. Biomech. Model. Mechanobiol..

[198] Bueno J., Bazilevs Y., Juanes R., Gomez H. (2017). Droplet Motion
Driven by Tensotaxis. Extreme Mech. Lett..

[199] Yang F., Chen P., Jiang H., Xie T., Shao Y., Kim D. H., Li B., Sun Y. (2024). Directional
Cell Migration Guided by a Strain Gradient. Small.

[200] Xie J., Macewan M. R., Ray W. Z., Liu W., Siewe D. Y., Xia Y. (2010). Radially Aligned, Electrospun Nanofibers
as Dural Substitutes for
Wound Closure and Tissue Regeneration Applications. ACS Nano.

[201] Xie J., Li X., Lipner J., Manning C. N., Schwartz A. G., Thomopoulos S., Xia Y. (2010). “Aligned-to-Random”
Nanofiber Scaffolds for Mimicking the Structure of the Tendon-to-Bone
Insertion Site. Nanoscale.

[202] Porter S. L., Wadhams G. H., Armitage J. P. (2011). Signal
Processing
in Complex Chemotaxis Pathways. Nat. Rev. Microbiol..

[203] Xue J., Wu T., Qiu J., Xia Y. (2020). Spatiotemporally Controlling
the Release of Biological Effectors Enhances Their Effects on Cell
Migration and Neurite Outgrowth. Small Methods.

[204] Carter S. B. (1967). Haptotaxis and the Mechanism of Cell
Motility. Nature.

[205] Ricoult S. G., Kennedy T. E., Juncker D. (2015). Substrate-Bound
Protein
Gradients to Study Haptotaxis. Front. Bioeng.
Biotechnol..

[206] Zhao Z. X., Chen X. Y., Dowbaj A. M., Sljukic A., Bratlie K., Lin L. D., Fong E. L., Balachander G. M., Chen Z. W., Soragni A., Huch M., Zeng Y. A., Wang Q., Yu H. (2022). Organoids. Nat. Rev. Methods Primers.

[207] Tang X.-Y., Wu S., Wang D., Chu C., Hong Y., Tao M., Hu H., Xu M., Guo X., Liu Y. (2022). Human Organoids in Basic Research and Clinical Applications. Signal Transduction Targeted Ther..

[208] Gjorevski N., Sachs N., Manfrin A., Giger S., Bragina M. E., Ordóñez-Morán P., Clevers H., Lutolf M. P. (2016). Designer Matrices for Intestinal
Stem Cell and Organoid Culture. Nature.

[209] Afting C., Walther T., Drozdowski O. M., Schlagheck C., Schwarz U. S., Wittbrodt J., Göpfrich K. (2024). DNA Microbeads for Spatio-Temporally Controlled Morphogen
Release within Organoids. Nat. Nanotechnol..

[210] Dang G., Wei Y., Wan Q., Gu J., Wang K., Wan M., Wang C., Song J., Mu Z., Tay F. R., Niu L. (2024). Regulatory Mechanisms and Regeneration
Strategies of the Soft-Hard Tissue Interface in the Human Periodontium. BMEMat.

[211] Rossetti L., Kuntz L. A., Kunold E., Schock J., Muller K. W., Grabmayr H., Stolberg-Stolberg J., Pfeiffer F., Sieber S. A., Burgkart R., Bausch A. R. (2017). The Microstructure
and Micromechanics of the Tendon-Bone Insertion. Nat. Mater..

[212] Lu H. H., Thomopoulos S. (2013). Functional
Attachment of Soft Tissues
to Bone: Development, Healing, and Tissue Engineering. Annu. Rev. Biomed. Eng..

[213] Dormer N. H., Berkland C. J., Detamore M. S. (2010). Emerging
Techniques
in Stratified Designs and Continuous Gradients for Tissue Engineering
of Interfaces. Ann. Biomed. Eng..

[214] Hao M., Chen Y., Thomopoulos S., Xia Y. (2025). Biomimetic Scaffolds
with Dual Gradients of Biological Effectors for Tendon-to-Bone Repair. Adv. Healthcare Mater..

[215] Lipner J., Shen H., Cavinatto L., Liu W., Havlioglu N., Xia Y., Galatz L. M., Thomopoulos S. (2015). *In
Vivo* Evaluation of Adipose-Derived Stromal Cells Delivered
with a Nanofiber Scaffold for Tendon-to-Bone Repair. Tissue Eng. Part A.

[216] Yang R., Li G., Zhuang C., Yu P., Ye T., Zhang Y., Shang P., Huang J., Cai M., Wang L., Cui W., Deng L. (2021). Gradient Bimetallic
Ion-Based Hydrogels for Tissue Microstructure Reconstruction of Tendon-to-Bone
Insertion. Sci. Adv..

[217] Roth J. G., Huang M. S., Li T. L., Feig V. R., Jiang Y., Cui B., Greely H. T., Bao Z., Pasca S. P., Heilshorn S. C. (2021). Advancing Models of Neural Development
with Biomaterials. Nat. Rev. Neurosci..

[218] Pekkurnaz G., Wang X. (2022). Mitochondrial Heterogeneity
and Homeostasis
Through the Lens of a Neuron. Nat. Metab..

[219] Wälchli T., Bisschop J., Carmeliet P., Zadeh G., Monnier P. P., De Bock K., Radovanovic I. (2023). Shaping the
Brain Vasculature in Development and Disease in the Single-Cell Era. Nat. Rev. Neurosci..

[220] Radoszkiewicz K., Rybkowska P., Szymanska M., Krzesniak N. E., Sarnowska A. (2025). The Influence
of Biomimetic Conditions
on Neurogenic and Neuroprotective Properties of Dedifferentiated Fat
Cells. Stem Cells.

[221] Rosoff W. J., Urbach J. S., Esrick M. A., McAllister R. G., Richards L. J., Goodhill G. J. (2004). A New Chemotaxis Assay Shows the
Extreme Sensitivity of Axons to Molecular Gradients. Nat. Neurosci..

[222] Mortimer D., Pujic Z., Vaughan T., Thompson A. W., Feldner J., Vetter I., Goodhill G. J. (2010). Axon Guidance by
Growth-Rate Modulation. Proc. Natl. Acad. Sci.
U. S. A..

[223] Liu W., Thomopoulos S., Xia Y. (2012). Electrospun Nanofibers for Regenerative
Medicine. Adv. Healthcare Mater..

[224] Boufidis D., Garg R., Angelopoulos E., Cullen D. K., Vitale F. (2025). Bio-Inspired Electronics: Soft, Biohybrid,
and “Living” Neural Interfaces. Nat. Commun..

[225] Huang L., Gao J., Wang H., Xia B., Yang Y., Xu F., Zheng X., Huang J., Luo Z. (2020). Fabrication of 3D Scaffolds Displaying Biochemical Gradients along
Longitudinally Oriented Microchannels for Neural Tissue Engineering. ACS Appl. Mater. Interfaces.

[226] Rifes P., Isaksson M., Rathore G. S., Aldrin-Kirk P., Møller O. K., Barzaghi G., Lee J., Egerod K. L., Rausch D. M., Parmar M., Pers T. H., Laurell T., Kirkeby A. (2020). Modeling Neural Tube Development
by Differentiation
of Human Embryonic Stem Cells in a Microfluidic WNT Gradient. Nat. Biotechnol..

[227] Uzel S. G., Amadi O. C., Pearl T. M., Lee R. T., So P. T., Kamm R. D. (2016). Simultaneous or Sequential Orthogonal
Gradient Formation in a 3D Cell Culture Microfluidic Platform. Small.

[228] Vaduganathan M., Mensah G. A., Turco J. V., Fuster V., Roth G. A. (2022). The Global
Burden of Cardiovascular Diseases and Risk:
A Compass for Future Health. J. Am. Coll. Cardiol..

[229] Takada T., Sasaki D., Matsuura K., Miura K., Sakamoto S., Goto H., Ohya T., Iida T., Homma J., Shimizu T. (2022). Aligned
Human Induced
Pluripotent Stem Cell-Derived Cardiac Tissue Improves Contractile
Properties Through Promoting Unidirectional and Synchronous Cardiomyocyte
Contraction. Biomaterials.

[230] Wu Y., Wang L., Guo B., Ma P. X. (2017). Interwoven Aligned
Conductive Nanofiber Yarn/Hydrogel Composite Scaffolds for Engineered
3D Cardiac Anisotropy. ACS Nano.

[231] Liu S., Wang Z., Chen X., Han M., Xu J., Li T., Yu L., Qin M., Long M., Li M., Zhang H., Li Y., Wang L., Huang W., Wu Y. (2023). Multiscale Anisotropic
Scaffold Integrating 3D Printing and Electrospinning
Techniques as a Heart-on-a-Chip Platform for Evaluating Drug-Induced
Cardiotoxicity. Adv. Healthcare Mater..

[232] Cao Z., Clark A. T., Vite A., Corbin E. A. (2024). A Dynamic Gradient
Stiffness Material Platform to Manipulate Cardiac Fibroblasts’
Spatio-Temporal Behavior. Adv. Funct. Mater..

[233] Odedra D., Chiu L. L., Shoichet M., Radisic M. (2011). Endothelial
Cells Guided by Immobilized Gradients of Vascular Endothelial Growth
Factor on Porous Collagen Scaffolds. Acta Biomater..

[234] Qiu Y., Myers D. R., Lam W. A. (2019). The Biophysics
and Mechanics of Blood
from A Materials Perspective. Nat. Rev. Mater..

[235] Sarkar S., Schmitz-Rixen T., Hamilton G., Seifalian A. M. (2007). Achieving
the Ideal Properties for Vascular Bypass Grafts Using a Tissue Engineered
Approach: A Review. Med. Biol. Eng. Comput..

[236] Grant C. A., Twigg P. C. (2013). Pseudostatic and
Dynamic Nanomechanics
of the Tunica Adventitia in Elastic Arteries Using Atomic Force Microscopy. ACS Nano.

[237] Du F., Wang H., Zhao W., Li D., Kong D., Yang J., Zhang Y. (2012). Gradient Nanofibrous Chitosan/Poly
Varepsilon-Caprolactone Scaffolds as Extracellular Microenvironments
for Vascular Tissue Engineering. Biomaterials.

[238] Furman D., Auwerx J., Bulteau A. L., Church G., Couturaud V., Crabbe L., Davies K. J. A., Decottignies A., Gladyshev V. N., Kennedy B. K., Neretti N., Nizard C., Pays K., Robinton D., Sebastiano V., Watson R. E. B., Wang M. C., Woltjen K. (2025). Skin Health and Biological
Aging. Nat. Aging.

[239] Peña O. A., Martin P. (2024). Cellular and Molecular
Mechanisms
of Skin Wound Healing. Nat. Rev. Mol. Cell Biol..

[240] Wang S., Wu W.-Y., Yeo J. C. C., Soo X. Y. D., Thitsartarn W., Liu S., Tan B. H., Suwardi A., Li Z., Zhu Q., Loh X. J. (2023). Responsive
Hydrogel Dressings for
Intelligent Wound Management. BMEMat.

[241] Li Y., Li G., Chen Y., Zhao X., Wang Y., Liu J., Li Z. (2022). Gradient Modulus
Tissue Adhesive Composite for Dynamic
Wound Closure. Adv. Funct. Mater..

[242] Chen J., Fan Y., Dong G., Zhou H., Du R., Tang X., Ying Y., Li J. (2023). Designing Biomimetic
Scaffolds for Skin Tissue Engineering. Biomater.
Sci..

[243] Wang Y., Xu R., Luo G., Lei Q., Shu Q., Yao Z., Li H., Zhou J., Tan J., Yang S., Zhan R., He W., Wu J. (2016). Biomimetic
Fibroblast-Loaded Artificial Dermis with “Sandwich”
Structure and Designed Gradient Pore Sizes Promotes Wound Healing
by Favoring Granulation Tissue Formation and Wound Re-Epithelialization. Acta Biomater..

[244] Zhang Y., Wang C., Jiang W., Zuo W., Han G. (2017). Influence of Stage Cooling Method on Pore Architecture of Biomimetic
Alginate Scaffolds. Sci. Rep..

[245] Li R., Liu K., Huang X., Li D., Ding J., Liu B., Chen X. (2022). Bioactive Materials
Promote Wound Healing through Modulation
of Cell Behaviors. Adv. Sci..

[246] Hoang D. M., Pham P. T., Bach T. Q., Ngo A. T. L., Nguyen Q. T., Phan T. T. K., Nguyen G. H., Le P. T. T., Hoang V. T., Forsyth N. R., Heke M., Nguyen L. T. (2022). Stem Cell-Based
Therapy for Human Diseases. Signal Transduction
Targeted Ther..

[247] Du J., Yao Y., Wang M., Su R., Li X., Yu J., Ding B. (2022). Programmable Building
of Radially Gradient Nanofibrous
Patches Enables Deployment, Bursting Bearing Capability, and Stem
Cell Recruitment. Adv. Funct. Mater..

[248] Chatterjee K., Lin-Gibson S., Wallace W. E., Parekh S. H., Lee Y. J., Cicerone M. T., Young M. F., Simon C. G. (2010). The Effect of
3D Hydrogel Scaffold Modulus on Osteoblast
Differentiation and Mineralization Revealed by Combinatorial Screening. Biomaterials.

[249] Mo S. J., Lee J.-H., Kye H. G., Lee J. M., Kim E.-J., Geum D., Sun W., Chung B. G. (2020). A Microfluidic
Gradient Device for Drug Screening with Human iPSC-Derived Motoneurons. Analyst.

[250] Wang Y., Gao Y., Pan Y., Zhou D., Liu Y., Yin Y., Yang J., Wang Y., Song Y. (2023). Emerging Trends
in Organ-on-a-Chip Systems for Drug Screening. Acta Pharm. Sin. B.

[251] Kolahi Azar H., Gharibshahian M., Rostami M., Mansouri V., Sabouri L., Beheshtizadeh N., Rezaei N. (2024). The Progressive Trend
of Modeling and Drug Screening Systems of Breast Cancer Bone Metastasis. J. Biol. Eng..

[252] Zhu D., Trinh P., Li J., Grant G. A., Yang F. (2021). Gradient Hydrogels
for Screening Stiffness Effects on Patient-Derived Glioblastoma Xenograft
Cellfates in 3D. J. Biomed. Mater. Res., Part
A.

[253] Horowitz L., Rodriguez A., Dereli-Korkut Z., Lin R., Castro K., Mikheev A., Monnat R., Folch A., Rostomily R. (2020). Multiplexed Drug Testing of Tumor
Slices Using a Microfluidic Platform. npj Precis.
Oncol..

[254] Monfort A., Soriano-Navarro M., García-Verdugo J. M., Izeta A. (2013). Production of Human
Tissue-Engineered Skin Trilayer on a Plasma-Based
Hypodermis. J. Tissue Eng. Regener. Med..

[255] Huber B., Link A., Linke K., Gehrke S. A., Winnefeld M., Kluger P. J. (2016). Integration of Mature
Adipocytes
to Build-Up a Functional Three-Layered Full-Skin Equivalent. Tissue Eng., Part C.

[256] Bailey B. M., Nail L. N., Grunlan M. A. (2013). Continuous Gradient
Scaffolds for Rapid Screening of Cell-Material Interactions and Interfacial
Tissue Regeneration. Acta Biomater..

[257] Schuster B., Junkin M., Kashaf S. S., Romero-Calvo I., Kirby K., Matthews J., Weber C. R., Rzhetsky A., White K. P., Tay S. (2020). Automated Microfluidic
Platform for
Dynamic and Combinatorial Drug Screening of Tumor Organoids. Nat. Commun..

[258] Shen S., Zhang F., Zhang Y., Li Y., Niu Y., Pang L., Wang J. (2023). Construction of Multiple
Concentration
Gradients for Single-Cell Level Drug Screening. Microsyst. Nanoeng..

[259] Vega S. L., Kwon M. Y., Song K. H., Wang C., Mauck R. L., Han L., Burdick J. A. (2018). Combinatorial
Hydrogels
with Biochemical Gradients for Screening 3D Cellular Microenvironments. Nat. Commun..

[260] Liu H., Liu R., Chen K., Liu Y., Zhao Y., Cui X., Tian Y. (2023). Bioinspired Gradient
Structured Soft Actuators: From
Fabrication to Application. Chem. Eng. J..

[261] Chen Z., Wang H., Cao Y., Chen Y., Akkus O., Liu H., Cao C. (2023). Bio-Inspired
Anisotropic
Hydrogels and Their Applications in Soft Actuators and Robots. Matter.

[262] Giachini P., Gupta S., Wang W., Wood D., Yunusa M., Baharlou E., Sitti M., Menges A. (2020). Additive Manufacturing
of Cellulose-Based Materials with Continuous, Multidirectional Stiffness
Gradients. Sci. Adv..

[263] Jiao D., Zhu Q. L., Li C. Y., Zheng Q., Wu Z. L. (2022). Programmable Morphing Hydrogels for
Soft Actuators and Robots: From
Structure Designs to Active Functions. Acc.
Chem. Res..

[264] Liu K., Pan X., Chen L., Huang L., Ni Y., Liu J., Cao S., Wang H. (2018). Ultrasoft Self-Healing Nanoparticle-Hydrogel
Composites with Conductive and Magnetic Properties. ACS Sustainable Chem. Eng..

[265] Daya R., Xu C., Nguyen N.-Y. T., Liu H. H. (2022). Angiogenic
Hyaluronic Acid Hydrogels with Curcumin-Coated Magnetic Nanoparticles
for Tissue Repair. ACS Appl. Mater. Interfaces.

[266] Li T., Tsui G. C.-P., Wong C.-H., Tang C.-Y., Tang K., Tan Y. (2025). Stimulus-Responsive
Gradient Hydrogel Micro-Actuators Fabricated
by Two-Photon Polymerization-Based 4D Printing. Nanotechnol. Rev..

[267] Keplinger C., Sun J.-Y., Foo C. C., Rothemund P., Whitesides G. M., Suo Z. (2013). Stretchable, Transparent, Ionic Conductors. Science.

[268] Simińska-Stanny J., Nizioł M., Szymczyk-Ziółkowska P., Brozyna M., Junka A., Shavandi A., Podstawczyk D. (2022). 4D Printing of Patterned Multimaterial
Magnetic Hydrogel Actuators. Addit. Manuf..

[269] Luo R., Wu J., Dinh N. D., Chen C. H. (2015). Gradient Porous
Elastic Hydrogels with Shape-Memory Property and Anisotropic Responses
for Programmable Locomotion. Adv. Funct. Mater..

[270] Plesser H. E. (2018). Reproducibility vs. Replicability:
A Brief History
of a Confused Terminology. Front. Neuroinform..

[271] Sharifi S., Reuel N., Kallmyer N., Sun E., Landry M. P., Mahmoudi M. (2023). The Issue of Reliability and Repeatability
of Analytical Measurement in Industrial and Academic Nanomedicine. ACS Nano.

[272] Lee B. N., Pei E. J., Um J. (2019). An Overview of Information
Technology Standardization Activities Related to Additive Manufacturing. Prog. Addit. Manuf..

[273] Guzzi E. A., Tibbitt M. W. (2020). Additive Manufacturing of Precision
Biomaterials. Adv. Mater..

[274] Li W., Karnati S., Zhang Y. L., Liou F. (2018). Investigating and Eliminating
Powder Separation in Pre-Mixed Powder Supply for Laser Metal Deposition
Process. J. Mater. Process Technol..

[275] DePond P. J., Guss G., Ly S., Calta N. P., Deane D., Khairallah S., Matthews M. J. (2018). *In Situ* Measurements of Layer Roughness
During Laser Powder Bed Fusion Additive
Manufacturing Using Low Coherence Scanning Interferometry. Mater. Des..

[276] Papamatthaiou S., Menelaou P., El Achab
Oussallam B., Moschou D. (2025). Recent Advances in Bio-Microsystem
Integration and
Lab-on-PCB Technology. Microsyst. Nanoeng..

[277] Ahn S. H., Guo L. J. (2009). Large-Area Roll-to-Roll
and Roll-to-Plate
Nanoimprint Lithography: A Step toward High-Throughput Application
of Continuous Nanoimprinting. ACS Nano.

[278] Dendukuri D., Pregibon D. C., Collins J., Hatton T. A., Doyle P. S. (2006). Continuous-Flow Lithography for High-Throughput
Microparticle
Synthesis. Nat. Mater..

[279] Zhu C., Gemeda H. B., Duoss E. B., Spadaccini C. M. (2024). Toward
Multiscale, Multimaterial 3D Printing. Adv.
Mater..

[280] Paratore F., Bacheva V., Bercovici M., Kaigala G. V. (2022). Reconfigurable Microfluidics. Nat. Rev. Chem..

[281] Liu Y., Luo D., Wang T. (2016). Hierarchical Structures
of Bone and
Bioinspired Bone Tissue Engineering. Small.

[282] Wegst U. G. K., Bai H., Saiz E., Tomsia A. P., Ritchie R. O. (2015). Bioinspired Structural Materials. Nat. Mater..

[283] Wei J., Pan F., Ping H., Yang K., Wang Y., Wang Q., Fu Z. (2023). Bioinspired Additive Manufacturing
of Hierarchical Materials: From Biostructures to Functions. Research.

[284] DebRoy T., Mukherjee T., Wei H. L., Elmer J. W., Milewski J. O. (2021). Metallurgy, Mechanistic
Models and Machine Learning
in Metal Printing. Nat. Rev. Mater..

[285] Moosavi S. M., Jablonka K. M., Smit B. (2020). The Role of
Machine
Learning in the Understanding and Design of Materials. J. Am. Chem. Soc..

[286] Wijerathne B., Liao T., Ostrikov K., Sun Z. (2022). Bioinspired
Robust Mechanical Properties for Advanced Materials. Small Struct..

[287] Kokkinis D., Bouville F., Studart A. R. (2018). 3D Printing of Materials
with Tunable Failure *via* Bioinspired Mechanical Gradients. Adv. Mater..

[288] Suwardi A., Wang F., Xue K., Han M. Y., Teo P., Wang P., Wang S., Liu Y., Ye E., Li Z., Loh X. J. (2022). Machine Learning-Driven Biomaterials Evolution. Adv. Mater..

